# Artemether–lumefantrine with or without single-dose primaquine and sulfadoxine–pyrimethamine plus amodiaquine with or without single-dose tafenoquine to reduce *Plasmodium falciparum* transmission: a phase 2, single-blind, randomised clinical trial in Ouelessebougou, Mali

**DOI:** 10.1016/S2666-5247(24)00023-5

**Published:** 2024-07

**Authors:** Almahamoudou Mahamar, Merel J Smit, Koualy Sanogo, Youssouf Sinaba, Sidi M Niambele, Adama Sacko, Oumar M Dicko, Makonon Diallo, Seydina O Maguiraga, Yaya Sankaré, Sekouba Keita, Siaka Samake, Adama Dembele, Kjerstin Lanke, Rob ter Heine, John Bradley, Yahia Dicko, Sekou F Traore, Chris Drakeley, Alassane Dicko, Teun Bousema, Will Stone

**Affiliations:** a Malaria Research and Training Centre, Faculty of Pharmacy and Faculty of Medicine and Dentistry, University of Sciences Techniques and Technologies of Bamako, Bamako, Mali; b Department of Medical Microbiology and Radboud Center for Infectious Diseases, Radboud University Medical Center, Nijmegen, Netherlands; c Department of Pharmacy and Radboud Center for Infectious Diseases, Radboud University Medical Center, Nijmegen, Netherlands; d MRC International Statistics and Epidemiology Group, London School of Hygiene and Tropical Medicine, London, UK; e Department of Infection Biology, London School of Hygiene and Tropical Medicine, London, UK

## Abstract

**Background:**

Artemether–lumefantrine is widely used for uncomplicated *Plasmodium falciparum* malaria; sulfadoxine–pyrimethamine plus amodiaquine is used for seasonal malaria chemoprevention. We aimed to determine the efficacy of artemether–lumefantrine with and without primaquine and sulfadoxine–pyrimethamine plus amodiaquine with and without tafenoquine for reducing gametocyte carriage and transmission to mosquitoes.

**Methods:**

In this phase 2, single-blind, randomised clinical trial conducted in Ouelessebougou, Mali, asymptomatic individuals aged 10–50 years with *P falciparum* gametocytaemia were recruited from the community and randomly assigned (1:1:1:1) to receive either artemether–lumefantrine, artemether–lumefantrine with a single dose of 0·25 mg/kg primaquine, sulfadoxine–pyrimethamine plus amodiaquine, or sulfadoxine–pyrimethamine plus amodiaquine with a single dose of 1·66 mg/kg tafenoquine. All trial staff other than the pharmacist were masked to group allocation. Participants were not masked to group allocation. Randomisation was done with a computer-generated randomisation list and concealed with sealed, opaque envelopes. The primary outcome was the median within-person percent change in mosquito infection rate in infectious individuals from baseline to day 2 (artemether–lumefantrine groups) or day 7 (sulfadoxine–pyrimethamine plus amodiaquine groups) after treatment, assessed by direct membrane feeding assay. All participants who received any trial drug were included in the safety analysis. This study is registered with ClinicalTrials.gov, NCT05081089.

**Findings:**

Between Oct 13 and Dec 16, 2021, 1290 individuals were screened and 80 were enrolled and randomly assigned to one of the four treatment groups (20 per group). The median age of participants was 13 (IQR 11–20); 37 (46%) of 80 participants were female and 43 (54%) were male. In individuals who were infectious before treatment, the median percentage reduction in mosquito infection rate 2 days after treatment was 100·0% (IQR 100·0–100·0; n=19; p=0·0011) with artemether–lumefantrine and 100·0% (100·0–100·0; n=19; p=0·0001) with artemether–lumefantrine with primaquine. Only two individuals who were infectious at baseline infected mosquitoes on day 2 after artemether–lumefantrine and none at day 5. By contrast, the median percentage reduction in mosquito infection rate 7 days after treatment was 63·6% (IQR 0·0–100·0; n=20; p=0·013) with sulfadoxine–pyrimethamine plus amodiaquine and 100% (100·0–100·0; n=19; p<0·0001) with sulfadoxine–pyrimethamine plus amodiaquine with tafenoquine. No grade 3–4 or serious adverse events occurred.

**Interpretation:**

These data support the effectiveness of artemether–lumefantrine alone for preventing nearly all mosquito infections. By contrast, there was considerable post-treatment transmission after sulfadoxine–pyrimethamine plus amodiaquine; therefore, the addition of a transmission-blocking drug might be beneficial in maximising its community impact.

**Funding:**

Bill & Melinda Gates Foundation.

## Introduction

Artemisinin-based combination therapies (ACTs) retain excellent efficacy for treatment of uncomplicated malaria in Africa, despite concerning evidence for reduced sensitivity of parasites in east Africa to artemisinins.^1^^,^^2^ ACTs rapidly clear asexual parasites but have variable activity against mature gametocytes that are the parasite life stage that can be transmitted to mosquitoes. A pooled analysis of post-treatment microscopy data indicated that artemether–lumefantrine might be the most potent ACT in terms of gametocyte clearance whereas gametocyte persistence is markedly longer after the drug combination dihydroartemisinin–piperaquine.^3^ Importantly, post-treatment gametocyte carriage is an imperfect approximation of malaria transmission potential. Transmissible gametocytes might persist at submicroscopic densities after treatment and some antimalarial drugs might sterilise gametocytes before these are removed from the circulation.^4^ Although artemether–lumefantrine is the most widely used treatment regimen for uncomplicated *Plasmodium falciparum* infections globally,^5^ data on malaria transmission after artemether–lumefantrine treatment are conflicting. Five studies that performed mosquito feeding assays after artemether–lumefantrine treatment reached different conclusions. Three studies observed non-negligible transmission between 7 days and 14 days after treatment with artemether–lumefantrine^6^^,^^7^ or artemether–lumefantrine plus primaquine,^8^ whereas two others found near complete abrogation of transmission 1 week after initiation of artemether–lumefantrine treatment without primaquine.^9^^,^^10^ The only study that assessed transmission to mosquitoes before and after artemether–lumefantrine treatment saw only one (2%) of 49 individuals infect mosquitoes at day 7 after treatment initiation, with mosquito infection rates markedly reduced compared with pretreatment rates.^9^ Quantifying post-treatment transmission potential after the most commonly used antimalarial is important: if substantial transmission occurs after treatment with artemether–lumefantrine, the addition of a specific gametocytocide might need to be considered. WHO recommends that to reduce onward transmission of *P falciparum*, ACTs might be combined with a single low dose of primaquine (0·25 mg/kg),^11^ which has potent but short lived gametocytocidal activity. Although this recommendation is currently limited to areas of low transmission, it has received new attention in sub-Saharan Africa since the emergence of artemisinin resistance in Africa.^12^ As a *P falciparum* gametocytocide, one low dose of 0·25 mg/kg primaquine, when combined with ACTs, causes an immediate and effective reduction in the transmission of parasites^13^^,^^14^ and is safe for use without previous glucose-6-phosphate dehydrogenase (G6PD) status testing.^15^ Whether the addition of primaquine to artemether–lumefantrine is beneficial or whether artemether–lumefantrine already sufficiently prevents post-treatment transmission is currently unclear.

**Research in context**

**Evidence before this study**

We searched PubMed on June 13, 2023, with no restrictions on publication date or language, for studies assessing the post-treatment transmission of artemether–lumefantrine in combination with primaquine with search terms: “[Artemether-lumefantrine] OR [Coartem]” AND “[Primaquine] OR [Jasoprim] OR [Malirid] OR [Neo-Quipenyl] OR [Pimaquin] OR [Primachina] OR [Primacin] OR [Primaquina] OR [Remaquin]” AND “[Plasmodium falciparum]” AND “[Gametocytocidal] OR [Gametocytes]” AND “[Transmission]”. An additional search was done for studies assessing the post-treatment transmission of sulfadoxine–pyrimethamine plus amodiaquine in combination with tafenoquine with the following search terms: “[Sulfadoxine-pyrimethamine plus amodiaquine] OR [Sulfadoxine-pyrimethamine with amodiaquine] OR “[Fansidar] AND ([Camoquin] OR [Flavoquine])” AND “[Tafenoquine] OR [Krintafel] OR [Arakoda] OR [WR238605]” AND “[Gametocytocidal] OR [Gametocytes]” AND “[Transmission]”. Of the 19 results that fulfilled the initial search criteria, two studies conducted mosquito feeding assays following artemether–lumefantrine with primaquine treatment, yielding divergent outcomes. The remaining results encompassed the following: five studies that assessed safety or gametocyte carriage, or both, but did not include mosquito feeding assays, four studies that investigated different primaquine doses from the current study (0·1 mg/kg, 0·4 mg/kg, or 0·75 mg/kg, or a combination of these), and the remaining eight studies were not clinical trials. No results meeting the second search criteria were identified.

**Added value of this study**

Augmenting standard antimalarial treatments or mass administrations with a gametocytocidal drug might expedite efforts to achieve elimination and mitigate the spread of artemisinin-resistant *Plasmodium falciparum* strains. For specific treatment recommendations to be made, it is crucial we know how transmission potential is affected by distinct antimalarial therapies. Evaluating post-treatment transmission potential based solely on gametocyte carriage is inadequate because transmissible gametocytes might persist at submicroscopic densities following treatment, and certain antimalarial drugs can render gametocytes non-viable before their elimination from the circulation. Such assessments, therefore, require mosquito-feeding assays. Our study provides robust evidence that artemether–lumefantrine has potent transmission-blocking activity, even in the absence of primaquine. We also provide the first direct evidence of substantial transmission beyond a week after treatment with sulfadoxine–pyrimethamine plus amodiaquine. Notably, we provide the first data demonstrating that transmission after sulfadoxine–pyrimethamine plus amodiaquine can be annulled by administration of a single low dose of tafenoquine. Using direct assays of transmission and highly sensitive sex-specific gametocyte quantification, this investigation contributes to our understanding of the transmission-blocking activity of artemether-lumefantrine and strongly supports the incorporation of a gametocytocidal drug in combination with sulfadoxine–pyrimethamine plus amodiaquine.

**Implications of all the available evidence**

The gametocytocidal effects of artemether-lumefantrine observed in this study are consistent with the findings of earlier studies, suggesting that the inclusion of single low-dose primaquine might not be necessary when administering artemether–lumefantrine as antimalarial treatment in these age groups in areas where WHO recommends the addition of single low-dose primaquine. Growing concerns for the sustainability of artemether–lumefantrine as a first-line treatment might increase the utility of supplemental gametocytocides in future, either as part of standard treatments or in mass administrations. Our uniquely direct evidence of continued transmission following sulfadoxine–pyrimethamine plus amodiaquine is in agreement with previous observations of prolonged post-treatment gametocytaemia. These data strongly suggest that the community-wide benefits of seasonal malaria chemoprevention could be enhanced by the addition of gametocytocidal drug such as primaquine or tafenoquine. The community benefit of mass administration with 8-aminoquinolines for *P falciparum* transmission reduction is, to date, untested.

In addition, a study with tafenoquine, which is being developed as a long-lasting alternative to primaquine for preventing *Plasmodium vivax* relapse,^16^ demonstrated that one low dose of tafenoquine (1·66 mg/kg) in combination with dihydroartemisinin–piperaquine completely prevented infectivity within 7 days after initiation of treatment in Malian adults and children.^17^ Although this trial showed that tafenoquine can prevent transmission when co-administered with dihydroartemisinin–piperaquine, the effect appeared to be slower than primaquine.^18^ The combination of tafenoquine with non-ACTs to clear *P falciparum* gametocytes and prevent transmission has not been tested. Sulfadoxine–pyrimethamine plus amodiaquine, a non-artemisinin-based combined anti-malarial treatment, is the only antimalarial recommended for systematic mass administration in the form of seasonal malaria chemoprevention.^19^ As per WHO guidance, a non-ACT regimen is used for seasonal malaria chemoprevention due to the precaution that first-line or second-line malaria treatments should not be used for chemoprevention within the same country.^20^ Previous studies showed considerable post-treatment transmission potential following sulfadoxine–pyrimethamine plus amodiaquine administration, with infectivity and mosquito infection rate unaffected for the first 7 days after treatment and reductions in gametocytaemia only observed after 28 days.^7^^,^^14^ Increased gametocyte densities in the first 2 weeks following sulfadoxine–pyrimethamine plus amodiaquine will limit any effect of seasonal malaria chemoprevention on transmission.^7^ The combination of sulfadoxine–pyrimethamine plus amodiaquine with a single low dose of tafenoquine has never been tested.

In the current study, we aimed to assess the reduction of infectivity of *P falciparum* gametocytes following administration of the two widely used malaria treatments artemether–lumefantrine and sulfadoxine–pyrimethamine plus amodiaquine in combination with a single low dose of the gametocytocidal drugs primaquine and tafenoquine, respectively, in Malian children and adults.

## Methods

### Study design and participants

This four-group, single-blind, phase 2, randomised trial was conducted at the Ouelessebougou Clinical Research Unit of the Malaria Research and Training Centre (MRTC) of the University of Bamako in Mali. Ouelessebougou is a commune that includes the town of Ouelessebougou and 44 surrounding villages, with an estimated 50 000 inhabitants. Situated roughly 80 km to the south of Bamako, the capital of Mali, this area has a distinct seasonality in malaria transmission due to the rainy season from July to November. The prevalence of *P falciparum* malaria and gametocytes in children older than 5 years varies between 50% to 60% and 20% to 25%, respectively, during the transmission season. Before the commencement of screening, the study team met with community leaders, village health workers, and heads of households from each village to explain the study and obtain approval to conduct the study. Village health workers then used a door-to-door approach to inform households of the date when, and location where, consenting and screening would take place. Participants were included in the trial if they met the following criteria: positive for *P falciparum* gametocytes by microscopy (ie, ≥1 gametocytes recorded in a thick film against 500 white blood cells, equating to 16 gametocytes per μL with a standard conversion of 8000 white blood cells per μL blood); absence of other non-*P falciparum* species on blood film; haemoglobin density of at least 10 g/dL; G6PD normal (male >4 IU/g haemoglobin, female >6 IU/g haemoglobin); aged 10–50 years; bodyweight of 80 kg or less; no clinical signs of malaria, defined by fever (≥37·5°C); no signs of acute, severe, or chronic disease; and, consistent with the long half-life of tafenoquine, use of effective contraception for five half-lives (3 months) after the end of tafenoquine treatment. Exclusion criteria included pregnancy (tested at enrolment by urine or serum test, or both) or lactation, allergies to any of the study drugs, use of other medication (except for paracetamol or aspirin, or both), use of antimalarial drugs over the past week, signs of acute or chronic illness, history of psychiatric disorders, and blood transfusion in the past 90 days. A detailed list of inclusion and exclusion criteria is provided in appendix 4 (pp 29–30). Before screening and study enrolment, participants provided written informed consent (≥18 years) or assent with written parental consent (10–17 years).

Ethical approval was granted by the ethics committee of the Faculty of Medicine, Pharmacy, and Dentistry of the University of Science, Techniques, and Technologies of Bamako (Bamako, Mali; number 2021/189/CE/USTTB), and the research ethics committee of the London School of Hygiene and Tropical Medicine (London, UK; reference number 26257). The trial protocol is available in appendix 4 (pp 17–44). The trial is registered with ClinicalTrials.gov, NCT05081089.

### Randomisation and masking

Allocation to four treatment groups (artemether–lumefantrine, artemether–lumefantrine with primaquine [0·25 mg/kg], sulfadoxine–pyrimethamine plus amodiaquine, and sulfadoxine–pyrimethamine plus amodiaquine with tafenoquine [1·66 mg/kg]) was randomised in a 1:1:1:1 ratio. Enrolment continued until 80 participants were enrolled (20 individuals assigned to each treatment group). An independent MRTC statistician randomly generated the treatment assignment using Stata, version 16, which was linked to participant identification numbers. The statistician prepared sealed, opaque envelopes with the participant identification number on the outside and treatment assignment inside, which were sent to the MRTC study pharmacist. The study pharmacist provided treatment and was consequently not masked to treatment assignment; staff involved in assessing safety and laboratory outcomes were masked to group allocation. Participants were not masked to group allocation.

### Procedures

Artemether–lumefantrine treatment (Novartis, Basel, Switzerland) was administered over 3 days as per manufacturer instructions (appendix 4 p 2). A single dose of 0·25 mg/kg primaquine (ACE Pharmaceuticals, Zeewolde, Netherlands) was administered on day 0 in parallel with the first dose of artemether–lumefantrine, as described previously.^14^ Participants in the sulfadoxine–pyrimethamine plus amodiaquine groups were treated with standard doses of sulfadoxine–pyrimethamine plus amodiaquine (Guilin Pharmaceutical, Shanghai, China) as per manufacturer instructions (appendix 4 p 2). A single dose of tafenoquine (60° pharmaceuticals, Washington DC, USA) was administered on day 0 in parallel with the first doses of sulfadoxine–pyrimethamine and amodiaquine. Tafenoquine dosing was weight based to standardise efficacy and risk variance (appendix 4 p 3). The dose of 1·66 mg/kg was chosen on the basis of the good safety profile and efficacy in Malian adults and children aged at least 12 years.^17^ G6PD testing was conducted using both semi-quantitative (OSMMR-D G-6-PD test; R&D Diagnostics, Aghia Paraskevi, Greece) and quantitative testing (STANDARD G6PD Test; SD BIOSENSOR, Suwon, South Korea); inclusion in the study required normal enzyme function to be determined by both methods.

Participants received a full clinical and parasitological examination on days 2, 5, 7, 14, 21, and 28 after receiving the first dose of the study drugs (appendix 4 p 3). Giemsa-stained thick film microscopy was performed as described previously, with asexual stages counted against 200 white blood cells and gametocytes counted against 500 white blood cells.^14^ For molecular gametocyte quantification, total nucleic acids were extracted using a MagNAPure LC automated extractor (Total Nucleic Acid Isolation Kit-High Performance; Roche Applied Science, Indianapolis, IN, USA). Male and female gametocytes were quantified in a multiplex rRT-qPCR assay (appendix 4 p 4).^21^ Samples were classified as negative for a particular gametocyte sex if the qRT-PCR quantified density of gametocytes of that sex was less than 0·01 gametocytes per μL (ie, one gametocyte per 100 μL of blood sample). Haemoglobin density was measured using a haemoglobin analyser (HemoCue; AB Leo Diagnostics, Helsingborg, Sweden) or automatic haematology analyser (HumaCount 5D; Wiesbaden, Germany). Additional venous blood samples were taken for biochemical and infectivity assessments on days 0, 2, 5, 7, and 14 in all treatment groups. Aspartate transaminase, alanine transaminase, and blood creatine levels were determined using automatic biochemistry analyser Human 100 (Wiesbaden, Germany). For each assessment of infectivity, about 75 female *Anopheles gambiae* mosquitoes distributed into three cups, locally reared at the insectary, were allowed to feed for 15–20 min on venous blood samples (Lithium Heparin VACUETTE tube; Greiner Bio-One, Kremsmünster, Austria) through a prewarmed glass membrane feeder system (Coelen Glastechniek; Weldaad, Netherlands). Surviving mosquitoes were dissected on the seventh day after feeding; midguts were stained in 1% mercurochrome and examined for the presence and density of oocysts by expert microscopists.

### Outcomes

The primary outcome was the median within-person percent change in mosquito infection rate in infectious individuals from baseline to day 2 (artemether–lumefantrine groups) or from baseline to day 7 (sulfadoxine–pyrimethamine plus amodiaquine groups) after treatment. Secondary outcomes were mosquito infection metrics (oocyst density, mosquito infection rate, and infectivity to mosquitoes), gametocyte metrics (prevalence, density, circulation time, area under the curve [AUC] of density over time, and sex ratio [ie, proportion of gametocytes that were male]), and asexual and total parasite prevalence and density compared within treatment groups to baseline, at all feeding timepoints (days 2, 5, 7, 14, 21, and 28), and between treatment-matched groups. Primary and secondary analyses of mosquito infection rate and oocyst density metrics were performed on individuals infectious at baseline but are presented for all individuals in the appendix 4 (pp 5–6). Gametocyte infectivity was assessed as an exploratory outcome.

Safety assessments included haemoglobin and methaemoglobin density and median within-person percent change in density, and incidence of clinical and laboratory adverse events. Adverse events were graded by the study clinician for severity (mild, moderate, or severe) and relatedness to study medication (unrelated or unlikely, possibly, probably, or definitely related). A drop in haemoglobin concentration of 40% or more from baseline was categorised as a haematological severe adverse event. An external data safety and monitoring committee was assembled before the trial, and safety data were discussed after enrolment of 40 participants, and after the final follow-up visit of the last participant.

### Statistical analysis

Sample size estimation was based on infectivity for participants in previous trials in the same study setting using a mixed-effects logistic regression model that accounted for correlation between mosquito observations from the same participant.^13^^,^^14^^,^^17^^,^^18^ With an expected reduction in infectivity of 90% as previously detected for efficacious doses of primaquine and tafenoquine,^14^^,^^17^^,^^18^ we calculated 92% empirical power to detect more than 85% reduction in infectivity with a one-tailed test with a level of significance α of 0·05 when including 20 participants and dissecting 50 mosquitoes at each timepoint. When using an α of 0·025, empirical power was calculated at 90%. The sample size was not designed to compare the transmission-blocking effects between treatment groups; comparison of outcomes between groups was secondary and was only undertaken for treatment matching groups.

Clinical and entomological data were double entered into a Microsoft Access (version 365) database and analysed using Stata (version 16.0) and SAS (version 9.4). Statistical analyses of mosquito infection rate and oocyst density were analysed at timepoints after baseline only for those individuals who were infectious at baseline. The prevalence of gametocytes and infectious individuals were compared within and between treatment groups using one-sided Fisher’s exact tests. Haemoglobin concentrations were compared using paired *t* tests (*t* score) for within-group analyses and linear regression adjusted for baseline levels of each measure for between-group analyses (*t* score, coefficient with 95% CI). Percentage change from baseline was analysed using two-way *t* tests. The proportion of gametocytes that were male was analysed for all values with total gametocyte densities of 0·2 gametocytes per μL or more.^14^ Gametocyte circulation time was calculated to determine the mean number of days that a mature gametocyte circulates in the blood before clearance using a deterministic compartmental model that assumes a constant rate of clearance and has a random effect to account for repeated measures on individuals, as described previously.^22^ Difference in circulation time between groups and between gametocyte sexes was analysed using *t* tests (*t* score). AUC of gametocyte density per participant over time was calculated using the linear trapezoid method and was analysed by fitting linear regression models to the log_10_-adjusted AUC values, with adjustment for baseline gametocyte density (*t* score, coefficient with 95% CI). All other analyses of quantitative data were done using Wilcoxon sign rank tests (Z score) and Wilcoxon rank-sum tests (Z score). All comparisons were defined before study completion and analyses were not adjusted for multiple comparisons. Gametocyte infectivity was assessed using logistic regression models adjusted for gametocyte density, wherein the shape of the relationship between gametocyte density and mosquito infection rate was estimated using fractional polynomials. The outcome measure of this analysis was odds ratio (95% CI). For all analyses, the threshold for statistical significance was set at p<0·05.

### Role of the funding source

The funder of the study had no role in study design, data collection, data analysis, data interpretation, or writing of the report.

## Results

**Figure 1:**
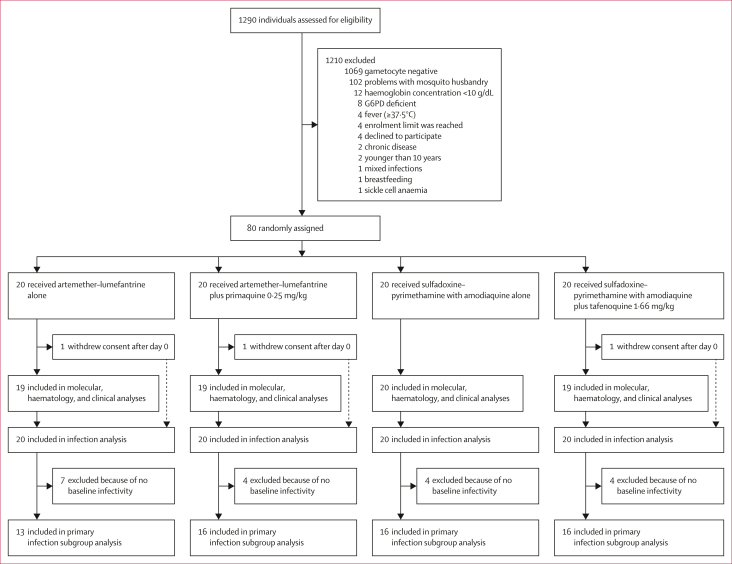
Trial profile

Between Oct 13 and Dec 16, 2021, 1290 individuals were assessed for eligibility and 80 were enrolled and randomly assigned to one of the four treatment groups (n=20 per group), of which 77 (96%) completed all follow-up visits (figure 1). Participant baseline characteristics were similar between the study groups (table 1). Overall, median age was 13 years (IQR 11–20); 37 (46%) of 80 participants were female and 43 (54%) were male.

**Table 1:** Baseline characteristics

	Artemether–lumefantrine (n=20)	Artemether–lumefantrine with primaquine (n=20)	Sulfadoxine–pyrimethamine plus amodiaquine (n=20)	Sulfadoxine–pyrimethamine plus amodiaquine with tafenoquine (n=20)
Age, years	14 (12–19)	12 (10–19)	14 (12–22)	13 (10–20)
Female	12 (60%)	7 (35%)	11 (55%)	7 (35%)
Male	8 (40%)	13 (65%)	9 (45%)	13 (65%)
Weight, kg	37·5 (30·0–49·6)	35·5 (27·3–48·3)	46·5 (31·0–58·0)	35·5 (25·0–55·5)
Haemoglobin, g/dL	11·5 (11·0–12·3)	12·4 (12·0–12·9)	12·4 (11·2–13·3)	12·8 (12·1–14·0)
Gametocyte prevalence	20 (100%)	20 (100%)	20 (100%)	20 (100%)
Gametocyte density, parasites per μL	41·3 (9·9–58·4)	30·4 (27·6–92·4)	36·4 (16·1–145·4)	24·5 (7·7–126·0)
Asexual parasite prevalence	12 (60%)	11 (55%)	12 (60%)	11 (55%)
Asexual parasite density, parasites per μL	400 (120–2340)	400 (120–1120)	440 (180–2060)	400 (80–2160)

Data are median (IQR) or n (%).

**Figure 2:**
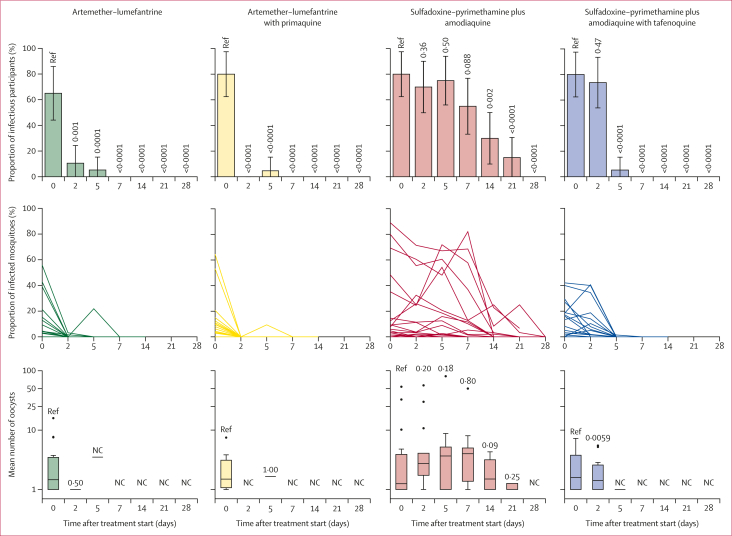
Participant infectivity and proportion of mosquitoes infected in direct membrane feeding assays For participant infectivity, p values are calculated from generalised linear models testing differences within treatment groups, with baseline as reference; error bars are 95% CI. The denominator for the proportion of infectious participants is the total number of participants still enrolled at a given timepoint, rather than the number tested at that timepoint for infectivity. For mosquito infection rate, each line represents one participant. For oocyst density, Wilcoxon signed-rank tests for differences in average oocyst density are shown. Box plots show the median (central line), IQR (box limits), upper and lower quartiles plus 1·5 × IQR (whiskers), and outliers for mean oocyst densities in infected mosquitoes within each participant. NC=not calculable.

Before treatment, 61 (76%) of 80 participants were infectious to mosquitoes, with a median of 9·7% (IQR 4·2–20·7) of mosquitoes becoming infected (table 2; appendix 4 p 5). At day 2, two (11%) of 19 participants in the artemether–lumefantrine group and zero (0%) of 19 participants in the artemether–lumefantrine with primaquine group infected mosquitoes (figure 2). In individuals who were infectious before treatment, the median percentage reduction in mosquito infection rate 2 days after treatment was 100·0% (IQR 100·0–100·0) for individuals treated with artemether–lumefantrine (n=19; p=0·0011) and 100·0% (IQR 100·0–100·0) with artemether–lumefantrine with primaquine (n=19; p=0·0001). Two participants (one in the sulfadoxine–pyrimethamine plus amodiaquine group and one in the sulfadoxine–pyrimethamine plus amodiaquine with tafenoquine group) were infectious on days 2 and 5, but not infectious on day 0. Furthermore, two participants (one participant in the artemether–lumefantrine group and one in the sulfadoxine–pyrimethamine plus amodiaquine group) were infectious on day 5, but not infectious on days 0 or 2. In addition, one participant in the artemether–lumefantrine with primaquine group was infectious at baseline and at day 5, but not at day 2. Post-treatment infectivity in these cases might stem from variations in drug susceptibility or exposure during distinct gametocyte development stages. At day 7, 11 (55%) of 20 participants in the sulfadoxine–pyrimethamine plus amodiaquine group and zero (0%) of the 19 participants in the sulfadoxine–pyrimethamine plus amodiaquine with tafenoquine group infected any number of mosquitoes. In individuals who were infectious before treatment, the median percentage reduction in mosquito infection rate 7 days after treatment was 63·6% (IQR 0·0–100·0) for individuals treated with sulfadoxine–pyrimethamine plus amodiaquine (n=11; p=0·013) and 100·0% (IQR 100·0–100·0) for individuals treated with sulfadoxine–pyrimethamine plus amodiaquine with tafenoquine (n=19; p<0·0001).

**Table 2:** Infectivity to mosquitoes before and after treatment

	Infectious individuals∗	Mosquito infection rate†	Reduction in mosquito infection rate‡	Within-group	Between-group
**Pretreatment**					
Artemether–lumefantrine	13/20 (65%)	9·4% (4·2 to 21·3)	··	··	··
Artemether–lumefantrine plus primaquine	16/20 (80%)	9·8% (5·3 to 13·8)	··	··	··
Sulfadoxine–pyrimethamine and amodiaquine	16/20 (80%)	9·3% (4·0 to 41·8)	··	··	··
Sulfadoxine–pyrimethamine and amodiaquine plus tafenoquine	16/20 (80%)	14·8% (3·2 to 23·0)	··	··	··
**Day 2**					
Artemether–lumefantrine	2/19 (11%)	0% (0 to 0)	100·0% (100·0 to 100·0)	0·0011	Ref
Artemether–lumefantrine plus primaquine	0/19 (0%)	0% (0 to 0)	100·0% (100·0 to 100·0)	0·0001	0·11
Sulfadoxine–pyrimethamine and amodiaquine	14/20 (70%)	11·4% (2·3 to 29·2)	26·0% (0·8 to 54·5)	0·11	Ref
Sulfadoxine–pyrimethamine and amodiaquine plus tafenoquine	14/19 (74%)	3·4% (1·5 to 16·4)	59·6% (9·3 to 84·5)	0·088	0·30
**Day 5**					
Artemether–lumefantrine	1/19 (5%)	0% (0 to 0)	100·0% (100·0 to 100·0)	0·0005	Ref
Artemether–lumefantrine plus primaquine	1/19 (5%)	0% (0 to 0)	100·0% (100·0 to 100·0)	0·0047	0·37
Sulfadoxine–pyrimethamine and amodiaquine	15/20 (75%)	14·2% (1·4 to 51·2)	24·5% (–49·3 to 71·1)	0·57	Ref
Sulfadoxine–pyrimethamine and amodiaquine plus tafenoquine	1/19 (5%)	0% (0 to 0)	100·0% (100·0 to 100·0)	0·0001	<0·0001
**Day 7**					
Artemether–lumefantrine	0/19 (0%)	0% (0 to 0)	100·0% (100·0 to 100·0)	0·0005	Ref
Artemether–lumefantrine plus primaquine	0/19 (0%)	0% (0 to 0)	100·0% (100·0 to 100·0)	0·0001	1·0
Sulfadoxine–pyrimethamine and amodiaquine	11/20 (55%)	7·8% (0·0 to 24·9)	63·6% (0·0 to 100·0)	0·013	Ref
Sulfadoxine–pyrimethamine and amodiaquine plus tafenoquine	0/19 (0%)	0% (0 to 0)	100·0% (100·0 to 100·0)	0·0001	0·0001

Data are n/N (%) or median (IQR), unless stated otherwise. Full details of mosquito feeding assay outcomes are in the appendix (pp 5–6).

∗ Individuals were classed as infectious if direct membrane feeding assays resulted in at least one mosquito with any number of oocysts. Mosquito infection measures (percentage infection and oocyst density) are shown for all participants who were infectious at baseline, and oocyst densities are from all infected mosquitoes.

† Median proportion of mosquitoes infected by each participant, where for each participant the mosquito infection rate was the number of mosquitoes infected as a proportion of all mosquitoes surviving to dissection.

‡ Median reduction (relative to baseline) in mosquito infection rate at the given timepoints. All values are for individuals who were infectious to mosquitoes before treatment (ie, infected any number of mosquitoes).

The median reduction in mosquito infection rate in the artemether–lumefantrine group was not significantly different from the artemether–lumefantrine with primaquine group at any timepoint. No mosquito infections were observed after day 5 in either artemether–lumefantrine treatment groups. In the sulfadoxine–pyrimethamine plus amodiaquine and sulfadoxine–pyrimethamine plus amodiaquine with tafenoquine groups, respectively, 14 (70%) of 20 and 14 (74%) of 19 individuals were infectious to mosquitoes at day 2, whereas 15 (75%) of 20 and one (5%) of 19 were infectious at day 5. Median reduction in mosquito infection rate in individuals infectious at baseline was significantly different between groups by day 5, when there was a 24·5% (IQR –49·3 to 71·1) reduction in the sulfadoxine–pyrimethamine plus amodiaquine group, and near total abrogation of infection with the addition of tafenoquine (100·0%, IQR 100·0 to 100·0; figure 2; table 2). In the sulfadoxine–pyrimethamine plus amodiaquine group, 11 (69%) of 16 individuals infectious to mosquitoes at baseline were still infectious a week later, and 3 weeks after treatment three (19%) of 16 remained infectious. The median oocyst density on day 2 in the sulfadoxine–pyrimethamine plus amodiaquine with tafenoquine group was significantly different from day 0 (p=0·0059). No other significant differences were found in median oocyst density within or between groups at any of the timepoints (figure 2; appendix 4 p 5). Mosquito infection measures for all participants (regardless of baseline infectivity) are shown in the appendix 4 (p 6).

**Figure 3:**
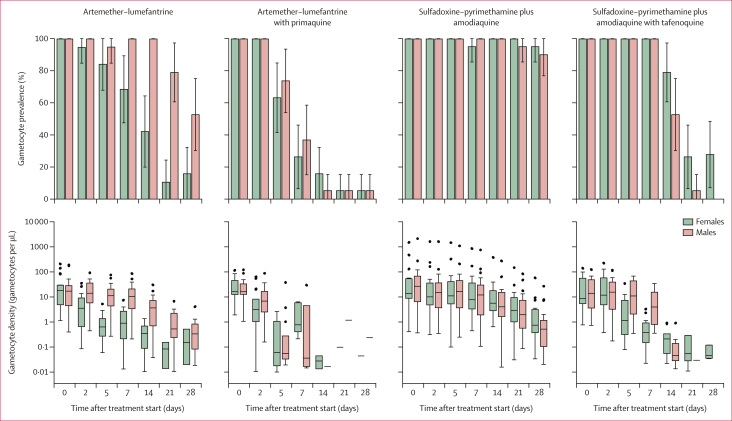
Male and female gametocyte density and prevalence Gametocyte prevalence estimates are shown with 95% CIs. Gametocyte density is shown for gametocyte-positive individuals only (ie, male or female density >0·01 per μL). Box plots show the median (central line), IQR (box limits), upper and lower quartiles plus 1·5 × IQR (whiskers), and outliers.

Gametocyte densities declined after initiation of treatment in all treatment groups, although the decrease was much less rapid in the sulfadoxine–pyrimethamine plus amodiaquine group than in any of the other groups (figure 3; appendix 4 pp 8, 10). All 20 (100%) participants treated with sulfadoxine–pyrimethamine plus amodiaquine alone remained gametocyte positive on the final day of observation (day 28), whereas 11 (58%) of 19 who received artemether–lumefantrine alone were still gametocyte positive at that same point. Total gametocyte circulation time was estimated at 5·3 days (95% CI 4·5–6·0) in the artemether–lumefantrine group and 2·9 days (2·4–3·3) in the artemether–lumefantrine with primaquine group (appendix 4 p 7); the same measure was estimated at 9·1 days (7·3–11·0) and 3·3 days (2·9–3·6) in the sulfadoxine–pyrimethamine plus amodiaquine and sulfadoxine–pyrimethamine plus amodiaquine with tafenoquine groups, respectively. Gametocyte sex ratios were initially similar in all treatment groups, but the ratio increased towards male in the artemether–lumefantrine group from day 2 after treatment (median proportion of male gametocytes 0·46 [IQR 0·37–0·51] on day 0 and 0·80 [0·60–0·92] on day 2; p=0·013]; appendix 4 pp 8, 10–11). In the sulfadoxine–pyrimethamine plus amodiaquine group, sex ratios remained largely unchanged but with an increasing bias toward females over the 28-day trial period. Treatment with primaquine and tafenoquine resulted in significantly male-biased ratios from day 5 (p=0·0023 and p<0·0001, respectively). The infectivity of persisting gametocytes was significantly lower in the sulfadoxine–pyrimethamine plus amodiaquine with tafenoquine group than in the sulfadoxine–pyrimethamine plus amodiaquine group (day 2 odds ratio [OR] 0·59 [95% CI 0·44–0·79], p<0·0001; day 5 OR 0·0077 [0·0011–0·056], p<0·0001), whereas in the artemether–lumefantrine groups too few gametocytes persisted to allow this comparison (appendix 4 p 11).

There were no haemolytic severe adverse events (ie, drop of >40% from baseline). Transient reductions in haemoglobin density were greater in the sulfadoxine–pyrimethamine groups than in the artemether–lumefantrine groups, with significant reductions in haemoglobin density observed at days 1 and 2 (during the period of treatment administration) in both sulfadoxine–pyrimethamine plus amodiaquine groups (mean change –5·0%); these resolved after day 5 (appendix 4 pp 12, 14). The maximum drop in haemoglobin between baseline and any study timepoint was 15·8% in the artemether–lumefantrine group, 15·9% in the artemether–lumefantrine with primaquine group, 24·3% in the sulfadoxine–pyrimethamine plus amodiaquine group and 23·5% in the sulfadoxine–pyrimethamine plus amodiaquine with tafenoquine group. The concentration in blood methaemoglobin was significantly higher in the artemether–lumefantrine with primaquine group compared with the artemether–lumefantrine group at day 1 (1·8 [0·5–3·0] *vs* 1·5 [0·6–2·3]; p=0·010) and in the sulfadoxine–pyrimethamine with amodiaquine plus tafenoquine group compared with the sulfadoxine–pyrimethamine with amodiaquine group on day 2 (1·8 [1·4–2·5] *vs* 1·5 [0·5–2·3]; p=0·029) and day 5 (1·9 [0·9–3·0] *vs* 1·6 [1·0–2·4]; p=0·020; appendix 4 pp 13–14).

Overall, 50 (63%) of the 80 participants experienced a total of 92 adverse events during follow-up, of which 61 were at least possibly related to the study drugs. 48 (79%) of the at least possibly related adverse events were categorised as mild and 13 (21%) as moderate. No grade 3 or serious adverse events occurred (table 3). The most common adverse events were headaches, abdominal pain, and nausea. No severe laboratory abnormalities occurred; all possibly drug-related laboratory abnormalities normalised on the subsequent visit (appendix 4 p 15).

**Table 3:** Frequency of adverse events

	All (n=80)	Artemether–lumefantrine (n=20)	Artemether–lumefantrine with primaquine (n=20)	Sulfadoxine–pyrimethamine plus amodiaquine (n=20)	Sulfadoxine–pyrimethamine plus amodiaquine with tafenoquine (n=20)
**All**	50 (62·5%)	6 (30%)	13 (65%)	16 (80%)	15 (75%)
p value	0·16∗	..	0·056†	..	1·00†
**Mild related adverse event**	29 (36%)	4 (20%)	9 (45%)	10 (50%)	6 (30%)
p value	0·37∗	..	0·18†	..	0·33†
**Moderate related adverse event**	9 (11%)	0	0	3 (15%)	6 (30%)
p value	0·012∗	..	NC	..	0·45†
**Severe related adverse event**	0	0	0	0	0
p value	NC	..	NC	..	nc

Data are n (%). If there were multiple episodes per participant, the highest grade is presented. Classification as related to treatment was defined as probably, possibly, or definitely related to treatment, as described in the methods. NC=not calculable.

∗ p values are from Fisher’s exact tests for differences in proportion of individuals with an adverse event between all groups.

† p values are from Fisher’s exact tests for differences in proportion of individuals with an adverse event between artemether–lumefantrine with primaquine group and the artemether–lumefantrine reference group and sulfadoxine–pyrimethamine plus amodiaquine with tafenoquine group and the sulfadoxine–pyrimethamine plus amodiaquine with tafenoquine reference group.

## Discussion

In this randomised trial, we determined the gametocytocidal and transmission-blocking activities of the most widely used first-line antimalarial, artemether–lumefantrine, with and without the WHO-recommended single low dose of 0·25 mg/kg of primaquine, and the preferred chemopreventative treatment, sulfadoxine–pyrimethamine plus amodiaquine, with and without one dose of 1·66 mg/kg tafenoquine. Artemether–lumefantrine alone blocked almost all transmission within 2 days of treatment initiation, whereas sulfadoxine–pyrimethamine plus amodiaquine had little effect on gametocyte density and prevalence, with significant reductions in infectivity to mosquitoes only observed after 14 days. Although the addition of tafenoquine did not prevent transmission at day 2 after treatment initiation, no infected mosquitoes were observed at the next mosquito feeding timepoint on day 5.

Calls for malaria eradication and concerns about the spread of drug resistance have increased interest in the effects of antimalarial drugs on gametocytes and their infectiousness.^23^ Since transmission cannot be reliably predicted from gametocyte densities and direct mosquito transmission outcomes are technically challenging, assessments of post-treatment infectiousness have been few and inconsistent. Previous studies demonstrated that the gametocytocidal effect of artemether–lumefantrine is superior to that of other ACTs, such as dihydroartemisinin–piperaquine and pyronaridine–artesunate,^24^^,^^25^ but were inconclusive on its ability to prevent transmission to mosquitoes early after treatment.^6^, ^7^, ^8^, ^9^, ^10^ In a selected population with high gametocyte densities and confirmed transmission potential before treatment, we demonstrate that artemether–lumefantrine exerts a strong gametocytocidal and transmission-blocking effect. Our finding of a median within-person reduction in mosquito infection rate of 100% 2 days after artemether–lumefantrine treatment corroborates findings from clinical trials in Kenya and The Gambia.^6^^,^^10^ Gametocyte densities also declined after artemether–lumefantrine, with drop of 44% (38·44 [IQR 9·15–56·47] to 21·61 [7·51–44·01] gametocytes per μL) between baseline and day 2. However, participants were still carrying theoretically transmissible densities of gametocytes at day 2,^26^ which suggests that the reduction in infectivity was due to sex ratio distortion or a sterilising effect, or both.^4^ In the current trial, we observed a 100% reduction in mosquito infection rate by day 5 for artemether–lumefantrine among individuals infectious to mosquitoes before treatment. However, there was an incomplete effect on gametocytes in two participants: one who was infectious on day 5 but not at baseline or on day 2, and another who was infectious at baseline and on day 5, but not on day 2. These instances of post-treatment infectivity might reflect differences in drug exposure of immature gametocytes that are sequestered in the bone marrow or spleen at the start of treatment but subsequently appear in circulation, or simply insufficient treatment of circulating mature gametocytes to prevent all transmission. It is therefore important that these observations are considered alongside our primary outcomes, and that further transmission studies are conducted to elucidate the gametocytocidal activity of artemether–lumefantrine against gametocytes at all stages of development, especially given the growing concerns of artemisinin resistance.

The potent transmission-blocking effect of artemether–lumefantrine contrasts with that of pyronaridine–artesunate and dihydroartemisinin–piperaquine, for which significant reductions in mosquito infection rate were only seen from 10 days and 7–14 days after treatment, respectively, when tested in the same facilities.^17^^,^^18^ For individuals treated with sulfadoxine–pyrimethamine plus amodiaquine, considerable and prolonged post-treatment transmission was observed. These data align with previous observations that show no change in transmission potential in the week following sulfadoxine–pyrimethamine plus amodiaquine treatment.^14^ Male and female gametocyte density declined initially and then increased slightly at day 5; these transient increases in gametocyte density following sulfadoxine–pyrimethamine plus amodiaquine have been observed previously.^7^ Here, ten (50%) of 20 individuals had more gametocytes at day 5 than at day 2 (in contrast to 5·3–15·8% in other treatment groups), which corresponded with increased infectiousness over the same time frame. Unlike in the artemether–lumefantrine groups and tafenoquine group, treatment with sulfadoxine–pyrimethamine plus amodiaquine did not cause any distortion in sex ratio. Although gametocyte prevalence in this group remained at 90% or more throughout the study, gametocyte densities declined in the majority of the participants to low, probably untransmissible levels by 21 days after treatment.^26^

As expected, the addition of one low dose of tafenoquine to sulfadoxine–pyrimethamine plus amodiaquine resulted in significant transmission-blocking activity among study participants. These results are consistent with previous studies in this setting, where the same low dose of tafenoquine (1·66 mg/kg) added to dihydroartemisinin–piperaquine led to complete transmission blockage at 7 days after treatment.^17^ Recent studies have shown suboptimal *P vivax* relapse rates after tafenoquine radical cure when co-administered with dihydroartemisnin–piperaquine.^27^ The reasons for this are unknown, but the combination appears effective for *P falciparum* transmission blockage, albeit with a delayed effect compared with primaquine.^17^ The current study, observing similar timing of the transmission-blocking effect when combined with a non-ACT, argues against putative artemisinin-specific antagonism or interaction as the cause of the delayed effect.^27^ In a controlled human malaria infection trial, the transmission-blocking efficacy of a single 50 mg dose of tafenoquine showed modest reduction of mosquito infection rate at day 4 of 35% and 81% by day 7.^28^ This dose is approximately half of that given in the current trial (1·66 mg/kg), which resulted in a median reduction in mosquito infection rate of 100% (IQR 100–100), while participant infectivity dropped from 14 (70%) to two (10%) of 20.^17^

A few limitations in our study warrant consideration. First, there are a large number of secondary analyses, and although effect sizes are large, caution should be taken when interpreting them due to issues of multiple testing. Second, the primary outcome of the current trial was *P falciparum* transmission, which required recruitment of high-density gametocyte carriers. With the assessment of infectivity before and after treatment, our findings are highly informative of the transmission-reducing activity of the tested drugs among highly infectious individuals. However, different study populations are required for assessments of community-level benefits of antimalarials combined with 8-aminoquinolines when given according to WHO guidelines (ie, at clinical presentation) or in mass treatment campaigns, including seasonal malaria chemoprevention. Third, our finding that a single low dose of primaquine appears to have little added benefit to transmission reduction when given with artemether–lumefantrine needs to be considered in light of possible use scenarios. Alternative ACTs with larger post-treatment transmission potential, such as dihydroartemisinin-piperaquine, are typically used in community treatment campaigns and the relevance of primaquine is likely to be larger. Moreover, gametocytes in *PfKelch13* mutant infections might preferentially survive artemisinin exposure and infect mosquitoes.^29^ The addition of a non-artemisinin gametocytocide (eg, primaquine or tafenoquine) to standard treatment might be a valuable tool to limit the spread of artemisinin partial resistance in Africa; the WHO malaria policy and advisory group suggested the broader adoption of single low-dose primaquine in countries where partial resistance has been detected.^12^ Finally, although tafenoquine is likely to have a comparatively prolonged transmission-blocking effect, its wider adoption is limited by concerns about potentially sustained haemolysis in G6PD-deficient individuals. Testing for G6PD deficiency is required before administration of tafenoquine for its current indications in treatment of *P vivax* (ie, 200 mg/day for 3 days or a single 300 mg dose).^30^ This study enrolled only individuals with normal G6PD enzyme function, to ensure comparability between groups. We observed no haematological, grade 3, or serious adverse events in any treatment group, and transient reductions in haemoglobin density that were not significantly different between groups with and without 8-aminoquinonlines. Our results reinforce the previous observations that single low doses of primaquine and tafenoquine are safe and well tolerated but longitudinal safety data for tafenoquine in G6PD-deficient individuals are needed.

In conclusion, our findings show that artemether–lumefantrine is able to prevent nearly all mosquito infections, even without primaquine, while maintaining a good safety profile. Furthermore, we observed considerable post-treatment transmission following the use of sulfadoxine–pyrimethamine plus amodiaquine. Hence, the addition of a transmission-blocking drug might be useful in maximising the community benefit of seasonal malaria chemoprevention.

## Data sharing

The data from this trial are accessible on the Clinical Epidemiology Database Resources website under study name NECTAR3.

## Declaration of interests

All authors declare no competing interests.
