# Supplementary material for: Artemether–lumefantrine with or without single-dose primaquine and sulfadoxine–pyrimethamine plus amodiaquine with or without single-dose tafenoquine to reduce Plasmodium falciparum transmission: a phase 2, single-blind, randomised clinical trial in Ouelessebougou, Mali
Source: Lancet Microbe. 2024 Jul;5(7):633–44. doi: 10.1016/S2666-5247(24)00023-5 (PMC11217006; doi:10.1016/S2666-5247(24)00023-5)
Supplement: Supplementary appendix 1 [file mmc1.pdf]

THE LANCET
Microbe

Supplementary appendix 1

This translation in French was submitted by the authors and we reproduce it as supplied. It has not been peer reviewed. *The Lancet*'s editorial processes have only been applied to the original in English, which should serve as reference for this manuscript.

Cette traduction en français a été proposée par les auteurs et nous l'avons reproduite telle quelle. Elle n'a pas été examinée par des pairs. Les processus éditoriaux du *Lancet* n'ont été appliqués qu'à l'original en anglais et c'est cette version qui doit servir de référence pour ce manuscrit.

Supplement to: Mahamar A, Smit MJ, Sanogo K, et al. Artemether–lumefantrine with or without single-dose primaquine and sulfadoxine–pyrimethamine plus amodiaquine with or without single-dose tafenoquine to reduce *Plasmodium falciparum* transmission: a phase 2, single-blind, randomised clinical trial in Ouelessebougou, Mali. *Lancet Microbe* 2024. https://doi.org/10.1016/S2666-5247(24)00023-5

Artéméther-luméfantrine combinée ou non à une dose unique de primaquine et sulfadoxine-pyriméthamine plus amodiaquine combinée ou non à une dose unique de tafénoquine pour réduire la transmission de *Plasmodium falciparum* : un essai clinique randomisé de phase 2, en simple aveugle, à Ouelessebougou, Mali.

Almahamoudou Mahamar*, Merel J Smit*, Koualy Sanogo, Youssouf Sinaba, Sidi M Niambele, Adama Sacko, Oumar M Dicko, Makonon Diallo, Seydina O Maguiraga, Yaya Sankaré, Sekouba Keita, Siaka Samake, Adama Dembele, Kjerstin Lanke, Rob ter Heine, John Bradley, Yahia Dicko, Sekou F Traoré, Chris Drakeley*, Alassane Dicko*, Teun Bousema*, Will Stone*

*Contribution égale

Malaria Research and Training Centre, Faculté de Pharmacie et Faculté de Médecine et d'Odonto-Stomatologie, Université des Sciences Techniques et Technologies de Bamako, Bamako, Mali (A Mahamar PhD, K Sanogo MD, Y Sinaba MD, S M Niambele PharmD, A Sacko MS, O M Dicko MD, M Diallo MD, S O Maguiraga MD, Y Sankaré MD, S Keita MS, S Samake Pharm D, A Dembele MS, Y Dicko MD, S F Traore PhD, Prof A Dicko MD); **Department of Medical Microbiology and Radboud Center for Infectious Diseases** (M J Smit MD, K Lanke PhD, Prof T Bousema PhD) et **Department of Pharmacy and Radboud Center for Infectious Diseases** (R ter Heine PhD), **Radboud University Medical Center, University of Nijmegen, Nijmegen, Netherlands; MRC International Statistics and Epidemiology Group** (J Bradley PhD) et **Department of Infection Biology**, **London School of Hygiene and Tropical Medicine, London, UK** (Prof C Drakeley PhD, W Stone PhD).

Correspondance à :
Dr Almahamoudou Mahamar, Malaria Research and Training Centre, Faculté de Pharmacie et Faculté de Médecine et d'Odonto-Stomatologie, Université des Sciences Techniques et Technologies de Bamako, Bamako, Mali
Almahamoudou Mahamar : almahamar@icermali.org

Résumé

Contexte : L'artéméther-luméfantrine est largement utilisé dans le traitement du paludisme simple à *Plasmodium falciparum* ; la sulfadoxine-pyriméthamine plus l'amodiaquine sont utilisées pour la chimioprévention du paludisme saisonnier. Notre objectif était de déterminer l'efficacité de l'artéméther-luméfantrine avec ou sans primaquine et de la sulfadoxine-pyriméthamine plus amodiaquine avec ou sans tafénoquine pour réduire le portage des gamétocytes et leur transmission aux moustiques.

Méthodes : Dans cet essai clinique randomisé en simple aveugle de phase 2 mené à Ouelessebougou, Mali, des personnes âgées de 10 à 50 ans porteurs asymptomatiques de gamétocytes de *P. falciparum* à l'examen microscopique ont été recrutées dans la communauté et

ont été randomisées (1:1:1:1) pour recevoir soit l'artéméther-luméfantrine, l'artéméther-luméfantrine avec une dose unique de 0,25 mg/kg de primaquine, la sulfadoxine-pyriméthamine plus amodiaquine, ou la sulfadoxine-pyriméthamine plus amodiaquine avec une dose unique de 1,66 mg/kg de tafénoquine. Tout le personnel de l'étude à l'exception du pharmacien était masqué pour la répartition des groupes. Les participants n'étaient pas aveugles pour la répartition des groupes. La randomisation a été effectuée à l'aide d'une liste de randomisation générée par ordinateur et dissimulée dans des enveloppes scellées et opaques. Le critère de jugement principal, évalué dans la population par protocole, était la variation médiane en pourcentage du taux d'infection par les moustiques entre le prétraitement et le jour 2 (groupes artéméther-luméfantrine) ou le jour 7 (groupes sulfadoxine-pyriméthamine plus amodiaquine) après le traitement, évalué par un test de gorgeage directe à travers une membrane. Tous les participants ayant reçu un médicament à l'essai ont été inclus dans l'analyse de sécurité. Cette étude est enregistrée auprès de ClinicalTrials.gov, NCT05081089.

Résultats : Entre le 13 octobre et le 16 décembre 2021, 1 290 personnes ont été dépistées et 80 ont été incluses et randomisées dans l'un des quatre groupes de traitement (20 par groupe). L'âge médian des participants était de 13 ans (IQR 11-20) ; 37 (46 %) des 80 participants étaient des femmes et 43 (54 %) étaient des hommes. Chez les personnes qui avaient infectées des moustiques avant le traitement, le pourcentage médian de réduction du taux d'infection pour les moustiques 2 jours après le traitement était de 100,0 % (IQR 100,0–100,0 ; n = 19 ; p = 0,0011) avec l'artéméther-luméfantrine et 100,0 % (100,0–100,0 ; n = 19 ; p = 0,0001) avec artéméther-luméfantrine et primaquine. Seuls deux personnes ayant infecté au prétraitement ont infecté des moustiques le deuxième jour après l'a de l'administration artéméther-luméfantrine et aucune au jour 5. En revanche, le pourcentage médian de réduction du taux d'infection pour les moustiques 7 jours après le traitement était de 63,6 % (IQR 0,0–100). ·0 ; n = 20 ; p = 0,013) avec sulfadoxine-pyriméthamine plus amodiaquine et 100 % (100,0–100,0 ; n = 19 ; p<0,0001) avec sulfadoxine-pyriméthamine plus amodiaquine avec tafénoquine. Aucun événement indésirable de grade 3 ou 4 ou grave n'est survenu.

Interprétation : Ces données soutiennent l'efficacité de l'artéméther-luméfantrine seul à prévenir presque toutes infections des moustiques. En revanche, il y a eu une transmission post-traitement considérable après l'administration de la sulfadoxine-pyriméthamine plus amodiaquine ; par conséquent, l'ajout d'un médicament bloquant la transmission pourrait être bénéfique pour maximiser son impact communautaire.

Financement : Fondation Bill & Melinda Gates.
