# Supplementary material for: Artemether–lumefantrine with or without single-dose primaquine and sulfadoxine–pyrimethamine plus amodiaquine with or without single-dose tafenoquine to reduce Plasmodium falciparum transmission: a phase 2, single-blind, randomised clinical trial in Ouelessebougou, Mali
Source: Lancet Microbe. 2024 Jul;5(7):633–44. doi: 10.1016/S2666-5247(24)00023-5 (PMC11217006; doi:10.1016/S2666-5247(24)00023-5)
Supplement: Supplementary appendix 2 [file mmc2.pdf]

THE LANCET
Microbe

Supplementary appendix 2

This translation in Spanish was submitted by the authors and we reproduce it as supplied. It has not been peer reviewed. *The Lancet*'s editorial processes have only been applied to the original in English, which should serve as reference for this manuscript.

Los autores nos proporcionaron esta traducción al español y la reproducimos tal como nos fue entregada. No la hemos revisado. Los procesos editoriales de *The Lancet* se han aplicado únicamente al original en inglés, que debe servir de referencia para este manuscrito.

Supplement to: Mahamar A, Smit MJ, Sanogo K, et al. Artemether–lumefantrine with or without single-dose primaquine and sulfadoxine–pyrimethamine plus amodiaquine with or without single-dose tafenoquine to reduce *Plasmodium falciparum* transmission: a phase 2, single-blind, randomised clinical trial in Ouelessebougou, Mali. *Lancet Microbe* 2024. https://doi.org/10.1016/S2666-5247(24)00023-5

Arteméter-lumefantrina con o sin primaquina en dosis única y sulfadoxina-pirimetamina más amodiaquina con o sin tafenoquina en dosis única para reducir la transmisión de Plasmodium falciparum: ensayo clínico aleatorizado, simple ciego, de fase 2 en Ouelessebougou (Malí).

Almahamoudou Mahamar*, Merel J Smit*, Koualy Sanogo, Youssouf Sinaba, Sidi M Niambele, Adama Sacko, Oumar M Dicko, Makonon Diallo, Seydina O Maguiraga, Yaya Sankaré, Sekouba Keita, Siaka Samake, Adama Dembele, Kjerstin Lanke, Rob ter Heine, John Bradley, Yahia Dicko, Sekou F Traore, Chris Drakeley*, Alassane Dicko*, Teun Bousema*, Will Stone*.
* Igual contribución

Malaria Research and Training Centre, Faculty of Pharmacy and Faculty of Medicine and Dentistry, University of Sciences Techniques and Technologies of Bamako, Bamako, Mali (A Mahamar PhD, K Sanogo MD, Y Sinaba MD, S M Niambele PharmD, A Sacko MS, O M Dicko MD, M Diallo MD, S O Maguiraga MD, Y Sankaré MD, S Keita MS, S Samake Pharm D, A Dembele MS, Y Dicko MD, S F Traore PhD, Prof A Dicko MD); Departamento de Microbiología Médica y Centro Radboud de Enfermedades Infecciosas (M J Smit MD, K Lanke PhD, Prof T Bousema PhD) y Departamento de Farmacia y Centro Radboud de Enfermedades Infecciosas (R ter Heine PhD), Centro Médico de la Universidad Radboud, Universidad de Nimega, Nimega, Países Bajos; MRC International Statistics and Epidemiology Group (J Bradley PhD) y Department of Infection Biology (Prof C Drakeley PhD, W Stone PhD), London School of Hygiene and Tropical Medicine, Londres, Reino Unido.

Correspondencia a:
Dr Almahamoudou Mahamar, Malaria Research and Training Centre, Faculty of Pharmacy and Faculty of Medicine and Dentistry, University of Sciences Techniques and Technologies of Bamako, Bamako, Mali

Almahamoudou Mahamar: almahamar@icermali.org

Resumen

Antecedentes El arteméter-lumefantrina se utiliza ampliamente para el paludismo no complicado *por Plasmodium falciparum*; la sulfadoxina-pirimetamina más amodiaquina se utiliza para la quimioprevención estacional del paludismo. El objetivo era determinar la eficacia de arteméter-lumefantrina con y sin primaquina y de sulfadoxina-pirimetamina más amodiaquina con y sin tafenoquina para reducir la carga de gametocitos y la transmisión a los mosquitos.

Métodos En este ensayo clínico de fase 2, simple ciego y aleatorizado, realizado en Ouelessebougou (Mali), se reclutó en la comunidad a individuos asintomáticos de 10 a 50 años con gametocitemia *por P falciparum* y se les asignó aleatoriamente (1:1:11) a recibir arteméter-lumefantrina, arteméter-lumefantrina con una dosis única de 0-25 mg/kg de primaquina, sulfadoxina-pirimetamina más amodiaquina, o sulfadoxina-pirimetamina más amodiaquina con una dosis única de 1-66 mg/kg de tafenoquina. La asignación de grupos estaba enmascarada para todo el personal del

ensayo, salvo el farmacéutico. No se ocultó a los participantes la asignación a los grupos. La aleatorización se realizó con una lista de aleatorización generada por ordenador y se ocultó con sobres opacos sellados. El resultado primario fue la mediana del cambio porcentual en la tasa de infección por mosquitos en individuos infecciosos desde el inicio hasta el día 2 (grupos de arteméter-lumefantrina) o el día 7 (grupos de sulfadoxina-pirimetamina más amodiaquina) después del tratamiento, evaluado mediante ensayo de alimentación directa por membrana. Todos los participantes que recibieron algún fármaco del ensayo fueron incluidos en el análisis de seguridad. Este estudio está registrado en ClinicalTrials.gov, NCT05081089.

Resultados Entre el 13 de octubre y el 16 de diciembre de 2021, se examinó a 1.290 individuos y se inscribió a 80, que fueron asignados aleatoriamente a uno de los cuatro grupos de tratamiento (20 por grupo). La edad media de los participantes fue de 13 años (IQR 11-20); 37 (46%) de los 80 participantes eran mujeres y 43 (54%) eran hombres**.** En los individuos que eran infecciosos antes del tratamiento, la mediana del porcentaje de reducción de la tasa de infección por mosquitos 2 días después del tratamiento fue del 100% (IQR 100-0-100-0; n=19; p=0-0011) con arteméter-lumefantrina y del 100% (100-0-100-0; n=19; p=0-0001) con arteméter-lumefantrina con primaquina. Sólo dos individuos que eran infecciosos al inicio infectaron a los mosquitos en el día 2 tras arteméter-lumefantrina y ninguno en el día 5. Por el contrario, la reducción porcentual media de la tasa de infección por mosquitos 7 días después del tratamiento fue del 63-6% (IQR 0-0-100-0; n=20; p=0-013) con sulfadoxina-pirimetamina más amodiaquina y del 100% (100-0-100-0; n=19; p<0-0001) con sulfadoxina-pirimetamina más amodiaquina con tafenoquina. No se produjeron acontecimientos adversos graves o de grado 3-4.

Interpretación Estos datos respaldan la eficacia de arteméter-lumefantrina sola para prevenir casi todas las infecciones por mosquitos. Por el contrario, hubo una transmisión considerable tras el tratamiento con sulfadoxina-pirimetamina más amodiaquina; por lo tanto, la adición de un fármaco bloqueador de la transmisión podría ser beneficioso para maximizar su impacto comunitario.

Financiación Fundación Bill y Melinda Gates.
