# Supplementary material for: Artemether–lumefantrine with or without single-dose primaquine and sulfadoxine–pyrimethamine plus amodiaquine with or without single-dose tafenoquine to reduce Plasmodium falciparum transmission: a phase 2, single-blind, randomised clinical trial in Ouelessebougou, Mali
Source: Lancet Microbe. 2024 Jul;5(7):633–44. doi: 10.1016/S2666-5247(24)00023-5 (PMC11217006; doi:10.1016/S2666-5247(24)00023-5)
Supplement: Supplementary appendix 3 [file mmc3.pdf]

THE LANCET
Microbe

Supplementary appendix 3

This translation in Kiswahili was submitted by the authors and we reproduce it as supplied. It has not been peer reviewed. *The Lancet*'s editorial processes have only been applied to the original in English, which should serve as reference for this manuscript.

Tafsiri hii katika Kiswahili iliwasilishwa na waandishi na tunatengeneza tena kama hutolewa. Haijapitiwa. Mchakato wa hariri wa Lancet umetumika tu kwa asili kwa Kiingereza, ambayo inapaswa kutumika kama kumbukumbu kwa muswada hii.

Supplement to: Mahamar A, Smit MJ, Sanogo K, et al. Artemether–lumefantrine with or without single-dose primaquine and sulfadoxine–pyrimethamine plus amodiaquine with or without single-dose tafenoquine to reduce *Plasmodium falciparum* transmission: a phase 2, single-blind, randomised clinical trial in Ouelessebougou, Mali. *Lancet Microbe* 2024. https://doi.org/10.1016/S2666-5247(24)00023-5

Artemether–lumefantrine ikiwa au bila kuwa na dozi moja ya primaquine na sulfadoxine–pyrimethamine pamoja na amodiaquine ikiwa au bila kuwa na dozi moja ya tafenoquine kupunguza maambukizi ya *Plasmodium falciparum*: Awamu ya 2, uliofumbwa upande mmoja**, jaribio la kisayansi la bahati nasibu huko Ouelessebougou, Mali**

Almahamoudou Mahamar, Merel J Smit*, Koualy Sanogo, Youssouf Sinaba, Sidi M Niambele, Adama Sacko, Oumar M Dicko, Makonon Diallo, Seydina O Maguiraga, Yaya Sankaré, Sekouba Keita, Siaka Samake, Adama Dembele, Kjerstin Lanke, Rob ter Heine, John Bradley, Yahia Dicko, Sekou F Traore, Chris Drakeley*, Alassane Dicko*, Teun Bousema*, Will Stone**

* Wote wamechangia kwa usawa

Kituo cha Utafiti na Mafunzo ya Malaria , Kitivo cha Famasia na Kitivo cha Dawa na Meno , Chuo Kikuu cha Sayansi na Teknolojia cha Bamako, Bamako, Mali (A Mahamar PhD, K Sanogo MD, Y Sinaba MD, S M Niambele PharmD, A Sacko MS, O M Dicko MD, M Diallo MD, S O Maguiraga MD, Y Sankaré MD, S Keita MS, S Samake Pharm D, A Dembele MS, Y Dicko MD, S F Traore PhD, Prof A Dicko MD); **Idara ya Matibabu ya microbiolojia na Radbound Kituo cha Magonjwa ya Kuambukiza** (M J Smit MD, K Lanke PhD, Prof T Bousema PhD**) na Idara ya Famasi na Radboundand kituo cha Magonjwa ya Kuambukiza (**R ter Heine PhD), **Chuo Kikuu cha Radboud kituo cha Matibabu, Chuo kikuu cha Nijmegen, Nijmegen, Netherlands; Umoja wa Kitaifa wa Takwimu na Epidemiolojia MRC** (J Bradley PhD) **na Idara ya Maambukizi ya Biolojia** (Prof C Drakeley PhD, W Stone PhD), **London School of Hygiene and Tropical Medicine, London, UK**

Mawasiliano ni kwa:
Dr Almahamoudou Mahamar, Kituo cha Utafiti na Mafunzo ya Malaria , Kitivo cha Famasia na Kitivo cha Dawa na Meno , Chuo Kikuu cha Sayansi na Teknolojia cha Bamako, Bamako, Mali
Almahamoudou Mahamar: almahamar@icermali.org

Muhtasari

Utangulizi Artemether–lumefantrine inatumika kwa wingi sana kwa *Plasmodium falciparum* malaria ambayo bado haijawa sugu; sulfadoxine–pyrimethamine pamoja na amodiaquine inatumika kuzuia malaria ya msimu

Tulilenga kuainisha ufanisi wa artemether–lumefantrine ikiwa au bila kuwa na primaquine na sulfadoxine–pyrimethamine pamoja na amodiaquine ikiwa au bila kuwa tafenoquine kwa ajili ya kupunguza ubebaji wa seli ya gametocyte na kusambaza kwa mbu.

Njia zilizotumika : Katika awamu hii ya 2, uliofumbwa upande mmoja, jaribio la kisayansi la bahati nasibu lililofanyika huko Ouelessebougou, Mali, watu wasio na dalili wenye umri kati ya miaka 10-50 pamoja na *P. falciparum* gametocytaemia walichaguliwa kutoka kwenye jamii na walipewa bila mpangilio (1:1:1:1) kupokea ama artemether–lumefantrine, artemether–lumefantrine pamoja na dozi moja ya 0.25 mg/kg ya primaquine, sulfadoxine–pyrimethamine pamoja na amodiaquine, au

sulfadoxine–pyrimethamine pamoja na amodiaquine na dozi moja tafenoquine yenye ujazo wa 1·66 mg/kg.
Wafanyakazi wote waliokuwa wakifanya majaribio isipokuwa tu wafamasia walifumbwa kwa ajili ya ugawaji wa makundi. Washiriki hawakufumbwa kwenye ugawaji wa makundi.Uchaguzi wa bahati nasibu ulifanywa kwa kompyuta ambapo ilitengenezwa orodha kwa bahati nasibu na kufichwa kwenye bahasha zilizofungwa kikamilifu. Matokeo ya awali yalikuwa ni ya wastani miongoni mwa asilimia ya watu waliobadilika kwa kuathiriwa na mbu na kiwango cha kuathirika kwa mtu mmoja mmoja kutoka kwenye utafiti wa awali kwenda siku ya 2 (Makundi ya artemether–lumefantrine) au siku ya 7 (makundi ya sulfadoxine–pyrimethamine pamoja na amodiaquine) baada ya matibabu walipimwa kwa kipimo cha moja kwa moja cha membrane feeding . Washiriki wote waliopokea jaribio lolote la dawa walijumuishwa kikamilifu kwenye uchambuzi wa usalama. Utafiti huu umesajiliwa na ClinicalTrials.gov, NCT05081089.

Matokeo Kati ya mwezi wa kumi tarehe 13 na mwezi wa kumi na mbili tarehe 16, 2021, watu 1290 walichujwa na 80 kati yao walichaguliwa na kwa bahati nasibu walipewa moja kati makundi manne ya matibabu yaliyoainishwa. (20 kwa kila kundi). Wastani wa umri wa washiriki ulikuwa 13(IQR 11-20);37 (46%) ya washiriki 80 walikuwa wanawake na 43 (54%) walikuwa wanaume. Kwa watu waliokuwa na maambukizi kabla ya matibabu, wastani wa asilimia za kupungua maambukizi kwa mbu kwa siku 2 baada ya matibabu ilikuwa ni 100.0% (IQR 100.0-100.0; n=19; p=0.0011) ikiwa na artemether–lumefantrine na 100·0% (100·0–100·0; n=19; p=0·0001) ikiwa na artemether–lumefantrine na primaquine.
Ni watu wawili tu ambao walikuwa wameambukizwa wakati wa utafiti wa awali kwa mbu walioambukizwa kwa siku ya 2 baada ya artemether–lumefantrine na hakuwa na yeyote kwa siku ya 5. Kwa utofauti, kiasi cha wastani wa asilimia za kupungua kwa mbu walioathirika siku ya 7 baada ya matibabu kilikuwa 63·6% (IQR 0·0–100·0; n=20; p=0·013) ikiwa na sulfadoxine–pyrimethamine pamoja na amodiaquine na 100% (100·0–100·0; n=19; p<0·0001) ikiwa na sulfadoxine–pyrimethamine na amodiaquine pamoja na tafenoquine. Hakukuwa na alama 3-4 au tukio lolote la hatari lililotokea.

Tafasiri. Takwimu hizi zinaunga mkono ufanisi wa artemether–lumefantrine peke yake kwa kuzuia maambukizi karibia (au kwa asilimia kubwa) kwa mbu wote. Kwa kutofautisha, kulikuwa kuna maambukizi makubwa baada ya matibabu ya sulfadoxine–pyrimethamine pamoja na amodiaquine; Kwa hiyo, nyongeza ya dawa ya kuzuia maambukizi inaweza kuwa na manufaa na kuongeza faida zake kwa jamii

Mfadhili Taasisi ya Bill & Melinda Gates.
