# Supplementary material for: Artemether–lumefantrine with or without single-dose primaquine and sulfadoxine–pyrimethamine plus amodiaquine with or without single-dose tafenoquine to reduce Plasmodium falciparum transmission: a phase 2, single-blind, randomised clinical trial in Ouelessebougou, Mali
Source: Lancet Microbe. 2024 Jul;5(7):633–44. doi: 10.1016/S2666-5247(24)00023-5 (PMC11217006; doi:10.1016/S2666-5247(24)00023-5)
Supplement: Supplementary appendix 4 [file mmc4.pdf]

THE LANCET
Microbe

Supplementary appendix 4

This appendix formed part of the original submission and has been peer reviewed.
We post it as supplied by the authors.

Supplement to: Mahamar A, Smit MJ, Sanogo K, et al. Artemether–lumefantrine with or without single-dose primaquine and sulfadoxine–pyrimethamine plus amodiaquine with or without single-dose tafenoquine to reduce *Plasmodium falciparum* transmission: a phase 2, single-blind, randomised clinical trial in Ouelessebougou, Mali. *Lancet Microbe* 2024. https://doi.org/10.1016/S2666-5247(24)00023-5

Appendix

Supplementary information 1. Antimalarial treatment dosing..2
Supplementary figure 1. Schematic representation of sample collection and analysis pipeline3
Supplementary table 1. Primer sequences and qPCR conditions for PfMGET CCp4 assay..........4
Supplementary table 2. Infectivity to mosquitoes 5
Supplementary table 3. Infectivity to mosquitoes (all individuals)..6
Supplementary table 4. Gametocyte circulation time and area under the curve........................7
Supplementary table 5. Total gametocyte density, prevalence and sex ratio.............................8
Supplementary table 6. Asexuals density and prevalence 9
Supplementary table 7. Female (CCP4) and male (PfMGET) gametocyte density and prevalence 10
Supplementary figure 2. Proportion of gametocytes that were male..11
Supplementary table 8. Gametocyte infectivity 11
Supplementary table 9. Haemoglobin density and change 12
Supplementary table 10. Methaemoglobin concentration 13
Supplementary figure 3. Haemoglobin and methaemoglobin..14
Supplementary table 11. Biochemistry 15
References 16

Supplementary information 1. Antimalarial treatment dosing

A. Artemether-lumefantrine (AL)

Participants in the AL or AL-PQ arm were treated with standard doses of AL from day 0-2. AL treatment tablets containing 20/120 mg artemether/lumefantrine or 80/480 mg artemether/lumefantrine (Coartem, Novartis, Basel, Switzerland) were administered according to weight as per manufacturer guidelines shown below:

**Body weight (kg)**	**20/120 mg artemether/lumefantrine tablet**			**80/480 mg artemether/lumefantrine tablet**		
	**Day 0**	**Day 1**	**Day 2**	**Day 0**	**Day 1**	**Day 2**
**5 to < 15 kg**	2x 1 tablet	2x 1 tablet	2x 1 tablet	-	-	-
**15 to < 25 kg**	2x 2 tablets	2x 2 tablets	2x 2 tablets	-	-	-
**25 to < 35 kg**	2x 3 tablets	2x 3 tablets	2x 3 tablets	-	-	-
**≥ 35 kg**	-	-	-	2x 1 tablet	2x 1 tablet	2x 1 tablet

B. Primaquine (PQ)

Participants in the AL-PQ arm were given a single low dose of 0·25 mg/kg primaquine (ACE Pharmaceuticals, Zeewolde, The Netherlands) as is currently recommended by the World Health Organization. The single dose of PQ was given on day 0 in parallel with the first dose of AL, administered in an aqueous solution, according to a standard operating procedure (SOP) provided by Sanofi as previously done at the study site when PQ was combined with DP or SPAQ.[1,2]

C. Sulfadoxine-pyrimethamine plus amodiaquine (SPAQ)

Participants in the SPAQ and SPAQ-TQ arm were treated with standard doses of SPAQ (Guilin Pharmaceutical, Shanghai, China). SP tablets containing 500 mg sulfadoxine and 25 mg pyrimethamine and AQ tablets containing 150 mg amodiaquine were administered according to weight as per manufacturer guidelines shown below:

**Body weight**	**500/50 mg sulfadoxine/pyrimethamine tablet**		
	**Day 0**	**Day 1**	**Day 2**
**11 to 20 kg**	1x 1 tablet	1x 1 tablet	1x 1 tablet
**21 to 30 kg**	1x 1·5 tablets	1x 1·5 tablets	1x 1·5 tablets
**31 to 45 kg**	1x 2 tablets	1x 2 tablets	1x 2 tablets
**> 45 kg**	1x 3 tablets	1x 3 tablets	1x 3 tablets

**Body weight**	**150 mg amodiaquine tablet**		
	**Day 0**	**Day 1**	**Day 2**
**15 to 18 kg**	1x 1·5 tablets	1x 1 tablet	1x 1 tablet
**19 to 24 kg**	1x 1·5 tablets	1x 1·5 tablets	1x 1·5 tablets
**25 to 35 kg**	1x 2·5 tablets	1x 2·5 tablets	1x 2 tablets
**36 to 50 kg**	1x 3 tablets	1x 3 tablets	1x 3 tablets
**> 50 kg**	1x 4 tablets	1x 4 tablets	1x 3 tablets

D. Tafenoquine (TQ)

Participants in the SPAQ-TQ arm received a single dose of 1.66 mg/kg tafenoquine (60degrees Pharma, Washington, US) on day 0, following the first dose of SPAQ. A dose of 1.66 mg/kg TQ is equivalent to a 100 mg single dose in a 60 kg adult. The maximum dose chosen for the current study was based on the reported safety profile of TQ doses ≤300 mg, which is similar to that of standard PQ dosing (15 mg daily for 14 days) in adult G6PD heterozygous adult individuals. [3] 100mg tafenoquine tablets were available for this study, and were prepared into a 1 mg/mL solution in water for weight-based dosing in 5 kg bands as follows:

**Weight min**	**Weight max**	**TQ 1 mg/mL total (mL)**	**Water (mL)**	**Masking solution (mL)**
30	35	54·0	136·1	10
35·01	40	62·3	127·7	10
40·01	45	70·6	119·4	10
45·01	50	78·9	111·1	10
50·01	55	87·2	102·8	10
55·01	60	95·5	94·5	10
60·01	65	103·8	86·2	10
65·01	70	112·1	77·9	10
70·01	75	120·4	69·6	10
75·01	80	128·7	61·3	10

Supplementary figure 1. Schematic representation of sample collection and analysis pipeline

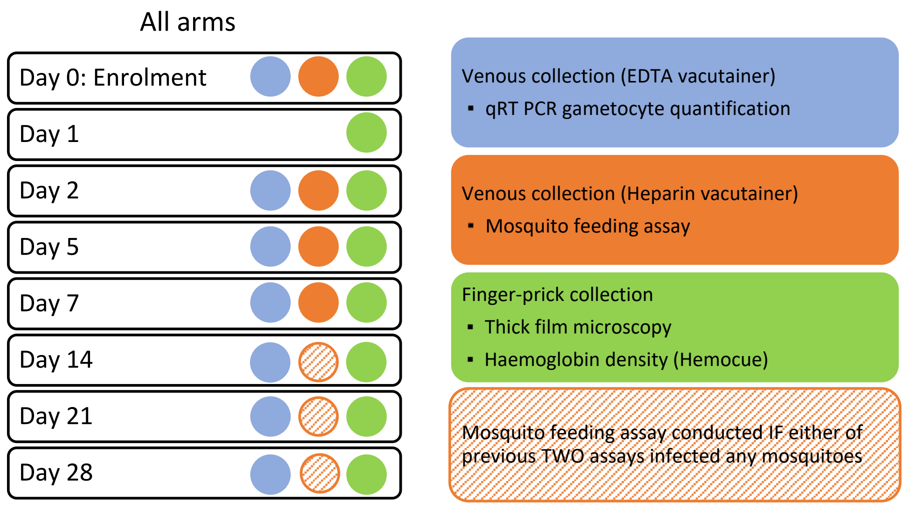

Supplementary table 1. Primer sequences and qPCR conditions for PfMGET CCp4 assay

PfMGET Primer/Probe Sequences

**Primers**	**Sequence**
Primer-FW (5'-3')	CGGTCCAAATATAAAAATCCTG
Primer-RV (5'-3')	TGTG TAACG TATG ATTCATTTTC
Probe (5'-3')	FAM-CAGCTCCAG CATTAAAACAC-BHQ1

CCp4 Primer/Probe Sequences

**Primers**	**Sequence**
Primer-FW (5'-3')	CACATGAATATGAGAATAAAATTG
Primer-RV (5'-3')	TAGGCGAACATGTGGAAAG
Probe (5'-3')	TexasRed-AGCAACAACGGTATGTGCCTTAAAACG-BHQ2

Male and female gametocyte quantification was performed as described previously, using a multiplex RT-qPCR assay.[4] Assays were run using commercial RT-qPCR mixes (Luna® Universal Probe One-Step RT-qPCR Kit, New England Biolabs, Ipswich, MA, USA). FW = Forward primer. RV = Reverse primer.

Supplementary table 2. Infectivity to mosquitoes

Day of follow-up	Treatment arm	Infectious individuals* n/N (%)	P-value§	P-value¶	Mosquito infection rate** Median % (IQR)	P-value§	P-value¶	Oocyst density*** Median (IQR)	P-value§	P-value¶
Day 0	Overall	61/80 (76%)	..	..	9·7 (4·2 to 20·7)	..	..	1·50 (1·00–3·46)	..	..
	AL	13/20 (65%)	*Reference*	*Reference*	9·4% (4·2 to 21·3)	*Reference*	*Reference*	1·46 (1·00–3·47)	*Reference*	*Reference*
	AL+PQ (0·25 mg/kg)	16/20 (80%)	*Reference*	0·24	9·8% (5·3 to 13·8)	*Reference*	0·88	1·50 (1·07–3·13)	*Reference*	0·93
	SPAQ	16/20 (80%)	*Reference*	*Reference*	9·3% (4·0 to 41·8)	*Reference*	*Reference*	1·25 (1·00–3·89)	*Reference*	*Reference*
	SPAQ+TQ (1·66 mg/kg)	16/20 (80%)	*Reference*	0·65	14·8% (3·2 to 23·0)	*Reference*	0·84	1·59 (1·00–3·78)	*Reference*	0·55
Day 2	AL	2/19 (11%)	0·001	*Reference*	0% (0 to 0)	0·0022	*Reference*	1·00 (1·00–1·00)	0.50	*Reference*
	AL+PQ (0·25 mg/kg)	0/19 (0%)	<0·0001	0·24	0% (0 to 0)	0·0007	0·11	*nc*	*nc*	*nc*
	SPAQ	14/20 (70%)	0·36	*Reference*	11·4% (2·3 to 29·2)	0·066	*Reference*	3·00 (1·73–4·08)	0·20	*Reference*
	SPAQ+TQ (1·66 mg/kg)	14/19 (74%)	0·47	0·54	3·4% (1·5 to 16·4)	0·056	0·33	1·25 (1·00–2·58)	0·0059	0·061
Day 5	AL	1/19 (5%)	0·0001	*Reference*	0% (0 to 0)	0·0022	*Reference*	*nc*	*nc*	*Reference*
	AL+PQ (0·25 mg/kg)	1/19 (5%)	<0·0001	0·76	0% (0 to 0)	0·0015	0·37	1·67 (1·67–1·67)	1·00	*nc*
	SPAQ	15/20 (75%)	0·50	*Reference*	14·2% (1·4 to 51·2)	0·72	*Reference*	4·00 (1·77–5·19)	0·18	*Reference*
	SPAQ+TQ (1·66 mg/kg)	1/19 (5%)	<0·0001	<0·0001	0% (0 to 0)	0·0004	<0·0001	*nc*	*nc*	*nc*
Day 7	AL	0/19 (0%)	<0·0001	*Reference*	0% (0 to 0)	0·0022	*Reference*	*nc*	*nc*	*Reference*
	AL+PQ (0·25 mg/kg)	0/19 (0%)	<0·0001	*nc*	0% (0 to 0)	0·0007	*nc*	*nc*	*nc*	*nc*
	SPAQ	11/20 (55%)	0·088	*Reference*	7·8% (0 to 24·9)	0·13	*Reference*	4·00 (1·38–5·00)	0·80	*Reference*
	SPAQ+TQ (1·66mg/kg)	0/19 (0%)	<0·0001	0·0001	0% (0 to 0)	0·0004	0·0001	*nc*	*nc*	*nc*
Day 14	AL	0/19 (0%)	<0·0001	*Reference*	..	*nc*	*Reference*	*nc*	*nc*	*Reference*
	AL+PQ (0·25 mg/kg)	0/19 (0%)	<0·0001	*nc*	0% (0 to 0)	0·32	*nc*	*nc*	*nc*	*nc*
	SPAQ	6/20 (30%)	0·002	*Reference*	0% (0 to 3·2)	0·0015	*Reference*	1·50 (1·00-3·17)	0·09	*Reference*
	SPAQ+TQ (1·66 mg/kg)	0/19 (0%)	<0·0001	0·012	..	*nc*	*nc*	*nc*	*nc*	*nc*
Day 21	AL	0/19 (0%)	<0·0001	*Reference*	..	*nc*	*Reference*	*nc*	*nc*	*Reference*
	AL+PQ (0·25 mg/kg)	0/19 (0%)	<0·0001	*nc*	..	*nc*	*nc*	*nc*	*nc*	*nc*
	SPAQ	3/20 (15%)	<0·0001	*Reference*	0% (0 to 3·3)	0·0033	*Reference*	1·00 (1·00-1·27)	0·25	*Reference*
	SPAQ+TQ (1·66 mg/kg)	0/19 (0%)	<0·0001	0·125	..	*nc*	*nc*	*nc*	*nc*	*nc*
Day 28	AL	0/19 (0%)	<0·0001	*Reference*	..	*nc*	*Reference*	*nc*	*nc*	*Reference*
	AL+PQ (0·25 mg/kg)	0/19 (0%)	<0·0001	*nc*	..	*nc*	*nc*	*nc*	*nc*	*nc*
	SPAQ	0/20 (0%)	<0·0001	*Reference*	0% (0 to 0)	0·043	*Reference*	*nc*	*nc*	*Reference*
	SPAQ+TQ (1·66mg/kg)	0/19 (0%)	<0·0001	nc	..	*nc*	*nc*	*nc*	*nc*	*nc*

*Individuals were classed as infectious if direct membrane feeding assays resulted in at least one mosquito with any number of oocysts. Mosquito infection measures (percent infection and oocyst density) are presented for all participants who were infectious at baseline, and oocyst densities are from all infected mosquitoes. **Mosquito infection rate = median percentage of mosquitoes infected by each participant, where the rate for each participant is number of mosquitoes infected as a percentage of all mosquitoes surviving to dissection. ***The average oocyst density for each participant was calculated as the mean number of oocysts in infected mosquitoes (i.e., with at least one oocyst). The value presented in the table is the median of all individuals' average oocyst intensities (a composite figure of all oocysts/all infected mosquitoes is not statistically valid). *nc* = not calculable, no positive observations. .. = not tested. P-value§ = within group comparison. P-value¶ = between group comparison (AL+PQ with AL reference group and SPAQ+TQ with SPAQ reference group). AL = artemether-lumefantrine; AL+PQ = artemether-lumefantrine with primaquine; SPAQ = sulfadoxine-pyrimethamine plus amodiaquine; SPAQ+TQ = sulfadoxine-pyrimethamine plus amodiaquine with tafenoquine.

Supplementary table 3. Infectivity to mosquitoes (all individuals)

Day of follow-up	Treatment arm	Mosquito infection rate* Median % (IQR)	P-value§	P-value¶	Oocyst density** Median (IQR)	P-value§	P-value¶	Median reduction in mosquito infection rate ‡ (IQR)	P-value§	P-value ¶
Day 0	Overall	5.5% (1.4 to 15.7)	..	..	0.08 (0.01-0.42)	..	..	..	..	..
	AL	3.7% (0 to 14.1)	*Reference*	*Reference*	0.04 (0-0.32)	*Reference*	*Reference*	..	..	..
	AL+PQ (0·25 mg/kg)	7.6% (3.0 to 11.8)	*Reference*	0.36	0.1 (0.03-0.3)	*Reference*	0.2989	..	..	..
	SPAQ	6.8% (1.4 to 24.9)	*Reference*	*Reference*	0.07 (0.01-0.65)	*Reference*	*Reference*	..	..	..
	SPAQ+TQ (1·66 mg/kg)	6.6% (1.5 to 18.7)	*Reference*	0.88	0.08 (0.01-0.62)	*Reference*	1.0	..	..	..
Day 2	AL	0% (0 to 0)	0.0008	*Reference*	0 (0-0)	0.0008	*Reference*	100% (0 to 100)	0.0005	*Reference*
	AL+PQ (0·25 mg/kg)	0% (0 to 0)	0.0003	0.15	0 (0-0)	0.0003	0.1518	100% (100 to 100)	0.0001	0.17
	SPAQ	3.6% (0 to 25.4)	0.066	*Reference*	0.09 (0-0.79)	0.3481	*Reference*	16.3% (0 to 44·3)	0.070	*Reference*
	SPAQ+TQ (1·66 mg/kg)	1.9% (0 to 14.0)	0.095	0.59	0.02 (0-0.48)	0.0507	0.5506	51.5% (0 to 80.6)	0.054	0.34
Day 5	AL	0% (0 to 0)	0.0050	*Reference*	0 (0-0)	0.005	*Reference*	100% (0 to 100)	0.0005	*Reference*
	AL+PQ (0·25 mg/kg)	0% (0 to 0)	0.0009	0.97	0 (0-0)	0.0006	0.9699	100% (0 to 100)	0.0010	0.73
	SPAQ	5.8% (0.7 to 34.4)	0.84	*Reference*	0.07 (0.01-1.87)	0.3599	*Reference*	7.1 (-30.4-48.6)	0.50	*Reference*
	SPAQ+TQ (1·66 mg/kg)	0% (0 to 0)	0.0003	<0.0001	0 (0-0)	0.0003	<0.0001	100% (100 to 100)	<0·0001	<0·0001
Day 7	AL	0% (0 to 0)	0.0008	*Reference*	0 (0-0)	0.0008	*Reference*	100% (0 to 100)	0.0005	*Reference*
	AL+PQ (0·25 mg/kg)	0% (0 to 0)	0.0003	*nc*	0 (0-0)	0.0003	*nc*	100% (100 to 100)	0·0001	0.48
	SPAQ	1.7% (0 to 12.8)	0.091	*Reference*	0.04 (0-0.45)	0.039	*Reference*	32·6% (0 to 93.5)	0.015	*Reference*
	SPAQ+TQ (1·66mg/kg)	0% (0 to 0)	0.0002	0.0002	0 (0-0)	0.0002	0.0002	100% (100 to 100)	<0·0001	0·0007
Day 14	AL	0% (0 to 0)	*nc*	*Reference*	0 (0-0)	*nc*	*Reference*	*nc**	*nc**	*Reference*
	AL+PQ (0·25 mg/kg)	0% (0 to 0)	0.32	*nc*	0 (0-0)	0.3173	*nc*	*nc**	*nc**	*nc**
	SPAQ	0% (0 to 3.2)	0.0009	*Reference*	0 (0-0.06)	0.0009	*Reference*	96.0% (65.1 to 100)	0.0008	*Reference*
	SPAQ+TQ (1·66 mg/kg)	0% (0 to 0)	*nc*	0.45	0 (0-0)	*nc*	0.4546	*nc**	*nc**	*nc**
Day 21	AL	0% (0 to 0)	*nc*	*Reference*	nc	*nc*	*Reference*	*nc**	*nc**	*Reference*
	AL+PQ (0·25 mg/kg)	0% (0 to 0)	*nc*	*nc*	nc	*nc*	*nc*	*nc**	*nc**	*nc**
	SPAQ	0% (0 to 3.3)	0.0033	*Reference*	0 (0-0.03)	0.0033	*Reference*	100 (97.1 to 100)	0.0022	*Reference*
	SPAQ+TQ (1·66 mg/kg)	0% (0 to 0)	*nc*	*nc*	nc	*nc*	*nc*	*nc**	*nc**	*nc**
Day 28	AL	0% (0 to 0)	*nc*	*Reference*	nc	*nc*	*Reference*	*nc**	*nc**	*Reference*
	AL+PQ (0·25 mg/kg)	0% (0 to 0)	*nc*	*nc*	nc	*nc*	*nc*	*nc**	*nc**	*nc**
	SPAQ	0% (0 to 0)	0.043	*Reference*	0 (0-0)	0.0431	*Reference*	100 (100-100)	0.025	*Reference*
	SPAQ+TQ (1·66mg/kg)	0% (0 to 0)	*nc*	*nc*	nc	*nc*	*nc*	*nc**	*nc**	*nc**

Mosquito infection measures (percent infection, percent reduction in mosquito infection rate and oocyst density) are presented for all participants regardless of baseline infectivity. *Mosquito infection rate = median percentage of mosquitoes infected by each participant, where the rate for each participant is number of mosquitoes infected as a percentage of all mosquitoes surviving to dissection. **The average oocyst density for each participant was calculated as the mean number of oocysts in infected mosquitoes (i.e., with at least one oocyst). The value presented in the table is the median of all individuals' average oocyst intensities (a composite figure of all oocysts/all infected mosquitoes is not statistically valid). **‡** Median within-person (relative to baseline) reduction in mosquito infection rate as in table 2 but including individuals not infectious at baseline (where zero infectivity at baseline and zero infectivity at follow-up results in 0% reduction). *nc* = not calculable, no positive observations. *nc** = not calculable, one or zero individuals remained infectious and participated in mosquito feeding. ..= not tested. P-value§ = within group comparison. P-value¶ = between group comparison (AL+PQ with AL reference group and SPAQ+TQ with SPAQ reference group). AL = artemether-lumefantrine; AL+PQ = artemether-lumefantrine with primaquine; SPAQ = sulfadoxine-pyrimethamine plus amodiaquine; SPAQ+TQ = sulfadoxine-pyrimethamine plus amodiaquine with tafenoquine.

Supplementary table 4. Gametocyte circulation time and area under the curve

	Treatment group	Total gametocytes (CCP4 & PfMGET)	P-value*	Female gametocytes (CCP4)	P-value*	Male gametocytes (PfMGET)	P-value*	P-value♂♀
**Circulation time Days (95% CI)**	AL	5·3 (4·5-6·0)	*Reference*	3·8 (3·1-4·5)	*Reference*	6·1 (5·1-7·1)	*Reference*	<0·0001
	AL+PQ (0·25 mg/kg)	2·9 (2·4-3·3)	<0·0001	2·7 (2·2-3·3)	0·017	3·1 (2·4-3·8)	<0·0001	0·0014
	SPAQ	9·1 (7·3-11·0)	*Reference*	11·0 (8·1-13·9)	*Reference*	7·7 (6·4-9·0)	*Reference*	0·0087
	SPAQ+TQ (1·66mg/kg)	3·3 (2·9-3·6)	<0·0001	3·7 (3·2-4·2)	<0·0001	2·7 (2·3-3·2)	<0·0001	0·0004
**AUC; Median (IQR) gametocytes per µL/day**	AL	11·6 (3·8–17·5)	*Reference*	1·2 (0·5–3·9)	*Reference*	9·3 (3·0–16·4)	*Reference*	<0·0001
	AL+PQ (0·25 mg/kg)	4·2 (3·2–8·9)	0·0012	1·0 (0·6–3·4)	0·46	2·6 (1·9–5·2)	0·0005	<0·0001
	SPAQ	17·8 (6·6–61·2)	*Reference*	8·7 (3·8–29·9)	*Reference*	10·7 (2·3–29·7)	*Reference*	0·86
	SPAQ+TQ (1·66mg/kg)	6·5 (2·0–27·0)	0·0091	2·1 (0·9–8·3)	<0·0001	4·8 (1·0–14·1)	0·41	<0·0001

Gametocyte circulation time was calculated using a deterministic compartmental model,[5] and is presented as the model estimate (mean days) with 95% CI. Area under the curve (AUC) of gametocyte density per participant over time was calculated using the linear trapezoid method,[6] and is presented as the median and IQR of individual AUC values by treatment arm. P-values are for differences in the t-statistic between AL+PQ and SPAQ+TQ treatment groups and the AL and SPAQ reference group, respectively (*), and for between sexes within treatment groups (♂♀).
AL = artemether-lumefantrine; AL+PQ = artemether-lumefantrine with primaquine; SPAQ = sulfadoxine-pyrimethamine plus amodiaquine; SPAQ+TQ = sulfadoxine-pyrimethamine plus amodiaquine with tafenoquine.

Supplementary table 5. Total gametocyte density, prevalence and sex ratio

		Total gametocytes (CCP4 & PfMGET)					
**Day of follow-up**	**Treatment arm**	**Median gametocytes/µL (IQR)**	**P-value¶**	**Prevalence n/N (%)**	**P-value¶**	**Proportion male Median (IQR)**	**P-value¶**
Day 0	*Overall*	30·44 (16·26–95·35)	..	78/80 (98%)	..	0·49 (0·40–0·56)	..
	AL	38·44 (9·15-56·47)	*Reference*	20/20 (100%)	*Reference*	0·46 (0·37–0·51)	*Reference*
	AL+PQ (0·25 mg/kg)	31·46 (27·86-77·43)	0·15	20/20 (100%)	*nc*	0·50 (0·37–0·59)	0·29
	SPAQ	53·03 (16·50-147·06)	*Reference*	18/20 (90%)	*Reference*	0·53 (0·45–0·66)	*Reference*
	SPAQ+TQ (1·66 mg/kg)	23·16 (9·01-110·65)	0·37	20/20 (100%)	0·244	0·48 (0·43–0·54)	0·20
Day 2	AL	21·61 (7·51–44·01)	*Reference*	19/19 (100%)	*Reference*	0·80 (0·60–0·92)	*Reference*
	AL+PQ (0·25 mg/kg)	9·97 (3·24–19·62)	0·24	19/19 (100%)	*nc*	0·65 (0·47–0·79)	0·057
	SPAQ	30·35 (12·49–81·57)	*Reference*	20/20 (100%)	*Reference*	0·49 (0·36–0·58)	*Reference*
	SPAQ+TQ (1·66 mg/kg)	27·27 (9·61–103·67)	0·83	19/19 (100%)	*nc*	0·48 (0·34–0·57)	0·79
Day 5	AL	11·15 (4·66–21·14)	*Reference*	18/19 (95%)	*Reference*	0·96 (0·87–0·98)	*Reference*
	AL+PQ (0·25 mg/kg)	0·05 (0·02–0·37)	0·0001	16/19 (84%)	0·30	0·67 (0·27–0·89)	0·0023
	SPAQ	31·20 (11·43–111·59)	*Reference*	20/20 (100%)	*Reference*	0·49 (0·38–0·57)	*Reference*
	SPAQ+TQ (1·66 mg/kg)	12·86 (2·89–56·40)	0·063	19/19 (100%)	*nc*	0·77 (0·65–0·94)	<0·0001
Day 7	AL	12·63 (3·40–20·88)	*Reference*	19/19 (100%)	*Reference*	0·98 (0·87–1·00)	*Reference*
	AL+PQ (0·25 mg/kg)	0 (0–0·21)	0·025	8/19 (42%)	0·0001	0·40 (0·08–0·43)	0·0003
	SPAQ	22·65 (6·81–86·89)	*Reference*	20/20 (100%)	*Reference*	0·47 (0·31–0·62)	*Reference*
	SPAQ+TQ (1·66 mg/kg)	4·76 (1·02–15·78)	0·010	18/19 (95%)	0·49	0·91 (0·85–0·96)	<0·0001
Day 14	AL	3·69 (0·70–7·20)	*Reference*	19/19 (100%)	*Reference*	1·00 (0·97–1·00)	*Reference*
	AL+PQ (0·25 mg/kg)	0 (0–0)	0·030	4/19 (21%)	<0·0001	*nc*	*nc*
	SPAQ	11·30 (5·75–38·27)	*Reference*	20/20 (100%)	*Reference*	0·40 (0·29–0·54)	*Reference*
	SPAQ+TQ (1·66 mg/kg)	0·08 (0·03–0·39)	0·0004	16/19 (84%)	0·11	0·24 (0·04–0·43)	0·099
Day 21	AL	0·40 (0·01–1·56)	*Reference*	15/19 (79%)	*Reference*	1·00 (1·00–1·00)	*Reference*
	AL+PQ (0·25 mg/kg)	0(0–0)	0·87	1/19 (5%)	<0·0001	0·92 (0·92–0·92)	0·14
	SPAQ	4·77 (2·06–17·66)	*Reference*	20/20 (100%)	*Reference*	0·40 (0·20–0·57)	*Reference*
	SPAQ+TQ (1·66 mg/kg)	0 (0–0·03)	0·004	6/19 (32%)	<0·0001	0 (0–0)	0·0095
Day 28	AL	0·02 (0–0·72)	*Reference*	11/19 (58%)	*Reference*	1·00 (0·79–1·00)	*Reference*
	AL+PQ (0·25 mg/kg)	0 (0–0)	0·75	2/19 (11%)	0·003	1·00 (1·00–1·00)	1·0
	SPAQ	1·50 (0·50–3·92)	*Reference*	20/20 (100%)	*Reference*	0·24 (0·11–0·48)	*Reference*
	SPAQ+TQ (1·66 mg/kg)	0 (0–0·03)	0·12	5/19 (26%)	<0·0001	*nc*	*nc*

P-values are for differences between AL+PQ and the AL reference group and SPAQ+TQ and the SPAQ reference group. Density was compared using regression analyses of log10 transformed density values, with adjustment for baseline densities. Prevalence was compared with one sided Fishers exact tests. For males and females, proportion male is given for participants/time-points with total gametocyte densities of 0·2/µL and over, as described previously (1). For the calculation of gametocyte prevalence, samples were classified as negative for a particular gametocyte sex if the estimated density of in gametocytes of that sex was less than 0·01/µL (i.e. one gametocyte per 100 µL of blood sample). P-value¶ = between group comparison (AL+PQ with AL reference group and SPAQ+TQ with SPAQ reference group). *Nc* = not calculable, no observations/no observations over the threshold density for analysis. .. = not tested. AL = artemether-lumefantrine; AL+PQ = artemether-lumefantrine with primaquine; SPAQ = sulfadoxine-pyrimethamine plus amodiaquine; SPAQ+TQ = sulfadoxine-pyrimethamine plus amodiaquine with tafenoquine.

Supplementary table 6. Asexuals density and prevalence

		Asexual parasites			
Day of follow-up	Treatment arm	Median asexuals/µL (IQR)	P-value¶	Prevalence n/N (%)	P-value¶
Day 0	*Overall*	132·0 (0·0–520·0)	..	54/80 (67·5%)	..
	AL	120·0 (0·0–590·0)	*Reference*	13/20 (65%)	*Reference*
	AL+PQ (0·25 mg/kg)	180·0 (0·0–420·0)	0.75	13/20 (65%)	0·63
	SPAQ	180·0 (0·0–660·0)	*Reference*	14/20 (70%)	*Reference*
	SPAQ+TQ (1·66 mg/kg)	120·0 (0·0–430·0)	0.86	14/20 (70%)	0·63
Day 2	AL	0·0 (0·0–0·0)	*Reference*	1/19 (5%)	*Reference*
	AL+PQ (0·25 mg/kg)	0·0 (0·0–0·0)	0.84	1/19 (5%)	0·76
	SPAQ	0·0 (0·0–0·0)	*Reference*	2/20 (10%)	*Reference*
	SPAQ+TQ (1·66 mg/kg)	0·0 (0·0–0·0)	0.78	1/19 (5%)	0·52
Day 5	AL	0·0 (0·0–0·0)	*Reference*	0/19 (0%)	*Reference*
	AL+PQ (0·25 mg/kg)	0·0 (0·0–0·0)	0.41	1/19 (5%)	1·00
	SPAQ	0·0 (0·0–0·0)	*Reference*	1/20 (5%)	*Reference*
	SPAQ+TQ (1·66 mg/kg)	0·0 (0·0–0·0)	0.42	0/19 (0%)	0·51
Day 7	AL	0·0 (0·0–0·0)	*Reference*	0/19 (0%)	*Reference*
	AL+PQ (0·25 mg/kg)	0·0 (0·0–0·0)	0.57	0/19 (0%)	*nc*
	SPAQ	0·0 (0·0–0·0)	*Reference*	0/20 (0%)	*Reference*
	SPAQ+TQ (1·66 mg/kg)	0·0 (0·0–0·0)	0.57	0/19 (0%)	*nc*
Day 14	AL	0·0 (0·0–0·0)	*Reference*	0/19 (0%)	*Reference*
	AL+PQ (0·25 mg/kg)	0·0 (0·0–0·0)	0.57	0/19 (0%)	*nc*
	SPAQ	0·0 (0·0–0·0)	*Reference*	0/20 (0%)	*Reference*
	SPAQ+TQ (1·66 mg/kg)	0·0 (0·0–0·0)	0.57	0/19 (0%)	*nc*
Day 21	AL	0·0 (0·0–0·0)	*Reference*	0/19 (0%)	*Reference*
	AL+PQ (0·25 mg/kg)	0·0 (0·0–0·0)	0.57	0/19 (0%)	*nc*
	SPAQ	0·0 (0·0–0·0)	*Reference*	0/20 (0%)	*Reference*
	SPAQ+TQ (1·66 mg/kg)	0·0 (0·0–0·0)	0.57	0/19 (0%)	*nc*
Day 28	AL	0·0 (0·0–0·0)	*Reference*	1/19 (5%)	*Reference*
	AL+PQ (0·25 mg/kg)	0·0 (0·0–0·0)	0.57	0/19 (0%)	0·5
	SPAQ	0·0 (0·0–0·0)	*Reference*	0/20 (0%)	*Reference*
	SPAQ+TQ (1·66 mg/kg)	0·0 (0·0–0·0)	0.57	0/19 (0%)	*nc*

P-values are for differences between AL+PQ and the AL reference group and SPAQ+TQ and the SPAQ reference group. Density was compared using regression analyses of log10 transformed density values, with adjustment for baseline densities. Prevalence was compared with one sided Fishers exact tests. P-value¶ = between group comparison (AL+PQ with AL reference group and SPAQ+TQ with SPAQ reference group). *nc* = not calculable, no observations/no observations over the threshold density for analysis. .. = not tested. AL = artemether-lumefantrine; AL+PQ = artemether-lumefantrine with primaquine; SPAQ = sulfadoxine-pyrimethamine plus amodiaquine; SPAQ+TQ = sulfadoxine-pyrimethamine plus amodiaquine with tafenoquine.

Supplementary table 7. Female (CCP4) and male (PfMGET) gametocyte density and prevalence

		**Female gametocytes (CCP4)**				**Male gametocytes (PfMGET)**			
**Day of follow-up**	**Treatment arm**	**Median/µL (IQR)**	**P-value**	**Prevalence n/N (%)**	**P-value**	**Median/µL (IQR)**	**P-value**	**Prevalence n/N (%)**	**P-value**
Day 0	*Overall*	15·19 (7·32–45·62)	..	78/80 (98%)	..	15·69 (5·46–45·85)	..	78/80 (98%)	..
	AL	18·18 (4·89–29·44)	*Reference*	20/20 (100%)	*Reference*	16·97 (4·47–28·31)	*Reference*	20/20 (100%)	*Reference*
	AL+PQ (0·25 mg/kg)	16·22 (12·52–45·29)	0·19	20/20 (100%)	*nc*	16·22 (12·66–33·14)	0·17	20/20 (100%)	*nc*
	SPAQ	16·75 (10·65–67·75)	*Reference*	18/20 (90%)	*Reference*	33·27 (7·75–86·13)	*Reference*	18/20 (90%)	*Reference*
	SPAQ+TQ (1·66 mg/kg)	8·61 (5·42–55·57)	0·68	20/20 (100%)	*nc*	13·90 (3·62–48·48)	0·22	20/20 (100%)	*nc*
Day 2	AL	2·77 (0·55–9·07)	*Reference*	18/19 (95%)	*Reference*	14·01 (5·82–35·79)	*Reference*	19/19 (100%)	*Reference*
	AL+PQ (0·25 mg/kg)	3·15 (1·03–8·18)	0·60	19/19 (100%)	0.50	6·82 (2·52–16·50)	0·065	19/19 (100%)	*nc*
	SPAQ	12·16 (5·72–44·93)	*Reference*	20/20 (100%)	*Reference*	18·26 (4·37–45·51)	*Reference*	20/20 (100%)	*Reference*
	SPAQ+TQ (1·66 mg/kg)	11·75 (4·85–57·78)	0·65	19/19 (100%)	*nc*	15·52 (3·24–39·11)	0·79	19/19 (100%)	*nc*
Day 5	AL	0·45 (0·08–1·27)	*Reference*	16/19 (84%)	*Reference*	9·68 (3·67–20·43)	*Reference*	18/19 (95%)	*Reference*
	AL+PQ (0·25 mg/kg)	0·02 (0–0·24)	0·60	12/19 (63%)	0.14	0·04 (0–0·28)	<0·0001	14/19 (74%)	0·090
	SPAQ	13·40 (6·29–51·42)	*Reference*	20/20 (100%)	*Reference*	20·21 (4·34–56·84)	*Reference*	20/20 (100%)	*Reference*
	SPAQ+TQ (1·66 mg/kg)	1·15 (0·32–7·29)	0·0001	19/19 (100%)	*nc*	10·97 (2·08–43·71)	0·39	19/19 (100%)	*nc*
Day 7	AL	0·21 (0–1·04)	*Reference*	13/19 (68%)	*Reference*	10·51 (3·39–20·88)	*Reference*	19/19 (100%)	*Reference*
	AL+PQ (0·25 mg/kg)	0 (0–0·21)	0·44	5/19 (26%)	0.011	0 (0–0·03)	0·027	7/19 (37%)	<0·0001
	SPAQ	9·09 (3·78–37·22)	*Reference*	19/20 (95%)	*Reference*	14·62 (2·21–37·16)	*Reference*	20/20 (100%)	*Reference*
	SPAQ+TQ (1·66 mg/kg)	0·38 (0·15–0·91)	<0·0001	18/19 (95%)	0.53	3·97 (0·80–15·47)	0·14	18/19 (95%)	*nc*
Day 14	AL	0 (0–0·32)	*Reference*	8/19 (42%)	*Reference*	3·65 (0·70–7·20)	*Reference*	19/19 (100%)	*Reference*
	AL+PQ (0·25 mg/kg)	0 (0–0)	0·60	3/19 (16%)	0.076	0 (0–0)	0·31	1/19 (0·1%)	<0·0001
	SPAQ	6·68 (3·30–21·88)	*Reference*	20/20 (100%)	*Reference*	4·78 (1·89–18·54)	*Reference*	20/20 (100%)	*Reference*
	SPAQ+TQ (1·66 mg/kg)	0·08 (0·02–0·26)	<0·0001	15/19 (79%)	0.047	0·01 (0–0·06)	0·0003	10/19 (53%)	0·0004
Day 21	AL	0 (0–0)	*Reference*	2/19 (0·1%)	*Reference*	0·40 (0·01–1·56)	*Reference*	15/19 (79%)	*Reference*
	AL+PQ (0·25 mg/kg)	0 (0–0)	*nc*	1/19 (0·1%)	0.50	0 (0–0)	0·77	1/19 (0·1%)	<0·0001
	SPAQ	3·41 (1·15–11·43)	*Reference*	20/20 (100%)	*Reference*	2·18 (0·47–6·89)	*Reference*	19/20 (95%)	*Reference*
	SPAQ+TQ (1·66 mg/kg)	0 (0–0·01)	0·020	5/19 (26%)	<0.0001	0 (0–0)	0·38	1/19 (0·1%)	<0·0001
Day 28	AL	0 (0–0)	*Reference*	3/19 (16%)	*Reference*	0·019 (0–0·42)	*Reference*	10/19 (53%)	*Reference*
	AL+PQ (0·25 mg/kg)	0 (0–0)	*nc*	1/19 (0·1%)	0.30	0 (0–0)	0·78	1/19 (0·1%)	0·002
	SPAQ	0·81 (0·35–3·16)	*Reference*	19/20 (95%)	*Reference*	0·47 (0·07–1·16)	*Reference*	18/20 (90%)	*Reference*
	SPAQ+TQ (1·66 mg/kg)	0 (0–0·03)	0·20	5/19 (26%)	<0.0001	0 (0–0)	*nc*	0/19 (0%)	<0·0001

P-values are for differences between AL+PQ and SPAQ+TQ groups and the reference AL and SPAQ group, respectively. Density was compared using regression analyses of log10 transformed density values, with adjustment for baseline densities. Prevalence was compared with one sided Fishers exact tests. For the calculation of gametocyte prevalence, samples were classified as negative for a particular gametocyte sex if the estimated density of in gametocytes of that sex was less than 0·01 gametocytes per µL (i.e. one gametocyte per 100 µL of blood sample). - = not tested. *ref* = reference group.
AL = artemether-lumefantrine; AL+PQ = artemether-lumefantrine with primaquine; SPAQ = sulfadoxine-pyrimethamine plus amodiaquine; SPAQ+TQ = sulfadoxine-pyrimethamine plus amodiaquine with tafenoquine.

Supplementary figure 2. Proportion of gametocytes that were male

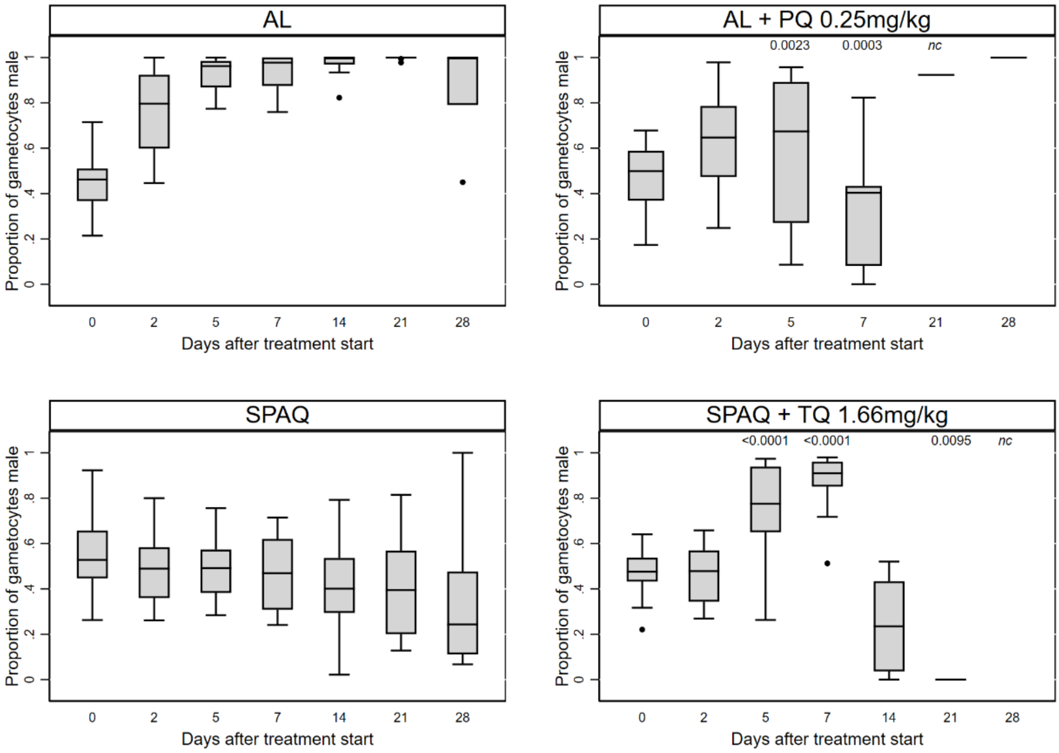

The proportion of gametocytes that were male was calculated for all values with total gametocyte densities of 0·2/µL and over, as described previously.[1] P-values (<0·05) for differences between treatment groups AL+PQ and SPAQ+TQ and the reference groups AL and SPAQ, respectively, were calculated using Wilcoxon rank sum tests.

Supplementary table 8. Gametocyte infectivity

Day of follow-up	Treatment arm	Odds ratio (95% CI)	P-value
Day 0	AL	1	*Reference*
	AL+PQ (0·25 mg/kg)	1·16 (0·88-1·51)	*0·289*
	SPAQ	1	*Reference*
	SPAQ+TQ (1·66 mg/kg)	0·95 (0·72-1·25)	*0·733*
Day 2	AL	1	*Reference*
	AL+PQ (0·25 mg/kg)	*nc*	*nc*
	SPAQ	1	*Reference*
	SPAQ+TQ (1·66 mg/kg)	0·59 (0·44-0·79)	<0·0001
Day 5	AL	1	*Reference*
	AL+PQ (0·25 mg/kg)	*nc*	*nc*
	SPAQ	1	*Reference*
	SPAQ+TQ (1·66 mg/kg)	0·0077 (0·001-0·05)	<0·0001
Day 7	AL	1	*Reference*
	AL+PQ (0·25 mg/kg)	*nc*	*nc*
	SPAQ	1	*Reference*
	SPAQ+TQ (1·66 mg/kg)	*nc*	*nc*

Odds ratios are for the change in mosquito infection rate in the AL+PQ arm compared to the reference (AL) arm and the SPAQ+TQ arm compared to the reference (SPAQ) arm with adjustment for total gametocyte densities. *nc* = not calculable, no observations (too few infected mosquitoes for convergence). AL = artemether-lumefantrine; AL+PQ = artemether-lumefantrine with primaquine; SPAQ = sulfadoxine-pyrimethamine plus amodiaquine; SPAQ+TQ = sulfadoxine-pyrimethamine plus amodiaquine with tafenoquine.

Supplementary table 9. Haemoglobin density and change

Day of follow-up	Treatment arm	Haemoglobin Mean g/dL (range)	P-value§	P-value¶	Percent change from day 0		P-value§	P-value¶
					Mean (lower/ upper 95% CI)	Range		
Day 0	*Overall*	12·3 (10·1–15·7)	..	..	..	..	..	..
	AL	11·5 (10·1–13·2)	*Reference*	*Reference*	..	..	..	..
	AL+PQ (0·25 mg/kg)	12·7 (10·7–15·7)	*Reference*	0·033	..	..	..	..
	SPAQ	12·4 (10·2–14·6)	*Reference*	*Reference*	..	..	..	..
	SPAQ+TQ (1·66 mg/kg)	12·8 (10·4–15·2)	*Reference*	0·14	..	..	..	..
Day 1	AL	11·4 (10·0–13·4)	0·60	*Reference*	-0·7 (-4·4/3·1)	-13·6/19·8	0·75	*Reference*
	AL+PQ (0·25 mg/kg)	12·3 (10·4–15·8)	0·051	0·77	-2·8 (-5·3/-0·2)	-13·0/5·7	0·054	0·38
	SPAQ	11·6 (9·9–14·0)	0·0012	*Reference*	-6·0 (-8·3/-3·6)	-16·9/1·6	0·0001	*Reference*
	SPAQ+TQ (1·66 mg/kg)	11·9 (10·1–13·9)	0·003	0·99	-6·4 (-10·0/-2·8)	-23·5/6·7	0·003	0·87
Day 2	AL	11·3 (9·9–13·1)	0·18	*Reference*	-1·8 (-4·7/1·0)	-12·9/15·8	0·24	*Reference*
	AL+PQ (0·25 mg/kg)	12·5 (11·2–15·8)	0·27	0·13	-1·2 (-3·6/1·2)	-10·4/14·3	0·35	0·76
	SPAQ	11·7 (10·1–13·6)	<0·0001	*Reference*	-5·0 (-6·8/-3·2)	-13·5/3·5	<0·0001	*Reference*
	SPAQ+TQ (1·66 mg/kg)	12·1 (10·2–14·3)	0·001	0·71	-5·0 (-7·5/-2·5)	-15·5/4·4	0·001	0·99
Day 5	AL	11·4 (10·1–12·5)	0·39	*Reference*	-1·1 (-4·3/2·1)	-15·2/18·8	0·53	*Reference*
	AL+PQ (0·25 mg/kg)	12·5 (11·0–14·9)	0·50	0·11	-0·7 (-3·1/1·7)	-11·2/14·3	0·57	0·87
	SPAQ	12·1 (10·5–14·6)	0·034	*Reference*	-1·9 (-3·5/-0·3)	-7·3/4·3	0·35	*Reference*
	SPAQ+TQ (1·66 mg/kg)	12·4 (10·6–14·2)	0·033	0·65	-3·0 (-5·6/-0·3)	-12·9/8·7	0·048	0·51
Day 7	AL	11·6 (9·9–12·9)	0·83	*Reference*	0·7 (-2·6/4·1)	-12·9/21·8	0·69	*Reference*
	AL+PQ (0·25 mg/kg)	12·3 (10·3–14·9)	0·004	0·37	-3·2 (-5·0/-1·3)	-10·4/5·0	0·004	0·060
	SPAQ	12·3 (10·6–14·6)	0·81	*Reference*	-0·2 (-3·9/3·5)	-24·3/11·4	0·93	*Reference*
	SPAQ+TQ (1·66 mg/kg)	12·6 (10·8–14·7)	0·25	0·84	-1·7 (-4·9/1·5)	-13·6/15·8	0·31	0·55
Day 14	AL	12·1 (11·1–14·4)	0·015	*Reference*	5·5 (1·8/9·3)	-8·3/25·7	0·012	*Reference*
	AL+PQ (0·25 mg/kg)	12·6 (11·0–15·0)	0·89	0·30	-0·1 (-2·3/2·1)	-9·8/5·7	0·93	0·019
	SPAQ	12·6 (11·1–15·0)	0·12	*Reference*	2·3 (-0·3/4·8)	-11·9/16·2	0·11	*Reference*
	SPAQ+TQ (1·66 mg/kg)	12·5 (11·0–14·8)	0·037	0·023	-2·0 (-3·8/-0·1)	-8·8/5·8	0·060	0·017
Day 21	AL	12·2 (11·0–13·6)	0·024	*Reference*	6·0 (1·8/10·2)	-15·4/23·8	0·015	*Reference*
	AL+PQ (0·25 mg/kg)	12·7 (11·1–15·0)	0·75	0·51	1·1 (-2·6/4·8)	-15·9/13·4	0·58	0·11
	SPAQ	12·6 (11·1–14·6)	0·046	*Reference*	2·7 (0·3/5·0)	-2·3/21·0	0·044	*Reference*
	SPAQ+TQ (1·66 mg/kg)	13·1 (11·4–15·8)	0·18	0·68	2·4 (-0·6/5·4)	-13·6/13·5	0·14	0·89
Day 28	AL	12·0 (10·8–13·3)	0·077	*Reference*	4·3 (0·3/8·4)	-11·4/25·7	0·058	*Reference*
	AL+PQ (0·25 mg/kg)	13·1 (11·5–15·4)	0·008	0·12	3·9 (1·4/6·4)	-6·4/10·3	0·007	0·87
	SPAQ	12·5 (10·0–14·5)	0·66	*Reference*	1·2 (-2·4/4·8)	-11·5/20·6	0·54	*Reference*
	SPAQ+TQ (1·66 mg/kg)	12·9 (11·2–15·2)	0·70	0·66	0·9 (-2·2/4·0)	-16·2/11·6	0·58	0·91

Haemoglobin density and percent change in haemoglobin density (relative to baseline) were compared within treatment arms (p-value§) using paired t-tests (with day 0 as reference for percent change) and between treatment arms (p-value¶) using linear regression (for density, adjusted for baseline Hb density at timepoints after baseline) or two-way t-tests (for percent reduction). AL = artemether-lumefantrine; AL+PQ = artemether-lumefantrine with primaquine; SPAQ = sulfadoxine-pyrimethamine plus amodiaquine; SPAQ+TQ = sulfadoxine-pyrimethamine plus amodiaquine with tafenoquine.

Supplementary table 10. Methaemoglobin concentration

Day of follow-up	Treatment arm	Mean Met Hb % (range)	P-value§	P-value¶
Day 0	*Overall*	1·6 (0·5–2·9)	..	..
	AL	1·6 (0·5–2·5)	*Reference*	*Reference*
	AL+PQ (0·25 mg/kg)	1·6 (0·7–2·9)	*Reference*	0·86
	SPAQ	1·7 (0·8–2·5)	*Reference*	*Reference*
	SPAQ+TQ (1·66 mg/kg)	1·7 (0·7–2·7)	*Reference*	0·76
Day 1	AL	1·5 (0·6–2·3)	0·11	*Reference*
	AL+PQ (0·25 mg/kg)	1·8 (0·5–3·0)	0·040	0·010
	SPAQ	1·6 (0·8–2·4)	0·18	*Reference*
	SPAQ+TQ (1·66 mg/kg)	1·8 (0·8–2·7)	0·43	0·17
Day 2	AL	1·6 (0·7–2·6)	0·42	*Reference*
	AL+PQ (0·25 mg/kg)	1·7 (0·6–3·1)	0·096	0·076
	SPAQ	1·5 (0·5–2·3)	0·11	*Reference*
	SPAQ+TQ (1·66 mg/kg)	1·8 (1·4–2·5)	0·31	0·029
Day 5	AL	1·6 (1·1–2·7)	0·59	*Reference*
	AL+PQ (0·25 mg/kg)	1·8 (1·0–4·3)	0·079	0·092
	SPAQ	1·6 (1·0–2·4)	0·23	*Reference*
	SPAQ+TQ (1·66 mg/kg)	1·9 (0·9–3·0)	0·16	0·020
Day 7	AL	1·5 (0·6–2·2)	0·31	*Reference*
	AL+PQ (0·25 mg/kg)	1·6 (0·8–3·8)	0·19	0·13
	SPAQ	1·6 (0·9–2·3)	0·14	*Reference*
	SPAQ+TQ (1·66 mg/kg)	1·8 (0·8–2·8)	0·46	0·083
Day 14	AL	1·4 (0·5–2·1)	0·078	*Reference*
	AL+PQ (0·25 mg/kg)	1·5 (0·5–2·8)	0·83	0·21
	SPAQ	1·5 (0·8–2·6)	0·039	*Reference*
	SPAQ+TQ (1·66 mg/kg)	1·6 (0·8–2·9)	0·86	0·28
Day 21	AL	1·4 (0·6–2·2)	0·17	*Reference*
	AL+PQ (0·25 mg/kg)	1·4 (0·7–2·7)	0·090	0·79
	SPAQ	1·3 (0·5–1·9)	0·0001	*Reference*
	SPAQ+TQ (1·66 mg/kg)	1·5 (0·5–2·0)	0·10	0·070
Day 28	AL	1·5 (0·8–2·8)	0·47	*Reference*
	AL+PQ (0·25 mg/kg)	1·3 (0·5–2·6)	0·15	0·36
	SPAQ	1·4 (0·7–2·6)	0·014	*Reference*
	SPAQ+TQ (1·66 mg/kg)	1·4 (0·7–2·2)	0·11	0·78

Methaemoglobin concentration (Met Hb %) was compared within treatment arms (p-value§) using paired t-tests and between treatment arms (p-value¶) using linear regression (for Met Hb %, adjusted for baseline Met Hb %). AL = artemether-lumefantrine; AL+PQ = artemether-lumefantrine with primaquine; SPAQ = sulfadoxine-pyrimethamine plus amodiaquine; SPAQ+TQ = sulfadoxine-pyrimethamine plus amodiaquine with tafenoquine.

Supplementary figure 3. Haemoglobin and methaemoglobin

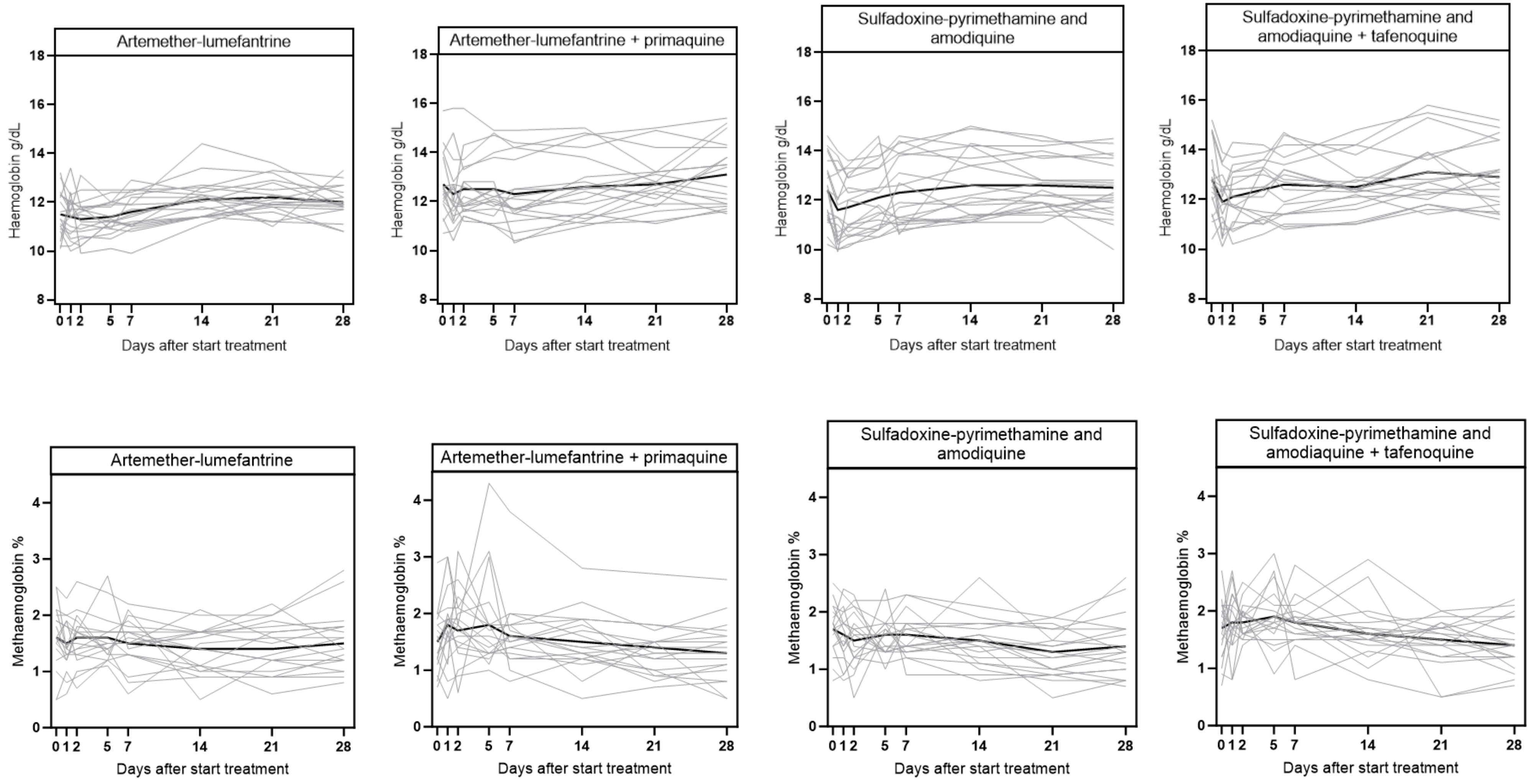

Absolute haemoglobin density and methaemoglobin concentration is given in grams per dL (y-axis, from 8-18 g/dL) and concentration (y-axis, from 0-4·5%), respectively, and is indicated for each participant individually with grey lines. The single black line shows the mean absolute haemoglobin density. P-values are presented in Supplementary table 9 and 10.

Supplementary table 11. Biochemistry

Day of follow-up	Treatment arm	Mean ALT U/L (range)	P-value[§]	P-value[¶]	Mean AST U/L (range)	P-value[§]	P-value[¶]	Mean creatinine mg/dL (range)	P-value[§]	P-value[¶]
Day 0	*Overall*	19·2 (3–105)	..	..	25·1 (4–91)	..	..	0·66 (0·10–1·15)	..	..
	AL	14·5 (3–28)	*Reference*	*Reference*	23·2 (4–55)	*Reference*	*Reference*	0·66 (0·42–1·11)	*Reference*	*Reference*
	AL+PQ (0·25 mg/kg)	22·9 (12–64)	*Reference*	0·015	26·8 (6–53)	*Reference*	0·34	0·66 (0·14–1·15)	*Reference*	0·95
	SPAQ	15·2 (8–29)	*Reference*	*Reference*	21·7 (4–44)	*Reference*	*Reference*	0·62 (0·10–1·14)	*Reference*	*Reference*
	SPAQ+TQ (1·66 mg/kg)	24·4 (5–105)	*Reference*	0·065	28·7 (9–91)	*Reference*	0·12	0·69 (0·24–1·14)	*Reference*	0·43
Day 2	*Overall*	21·4 (6–145)	..	..	25·6 (3–78)	..	..	0·67 (0·13–1·36)	..	..
	AL	15·8 (8–24)	0·36	*Reference*	26·5 (17–63)	0·17	*Reference*	0·55 (0·28–1·04)	0·032	*Reference*
	AL+PQ (0·25 mg/kg)	22·5 (10–56)	0·81	0·060	26·5 (4–57)	0·94	0·64	0·58 (0·17–1·04)	0·19	0·63
	SPAQ	19·2 (6–39)	0·013	*Reference*	23·3 (3–38)	0·49	*Reference*	0·76 (0·28–1·36)	0·055	*Reference*
	SPAQ+TQ (1·66 mg/kg)	28·3 (11–145)	0·23	0·32	26·3 (5–78)	0·35	0·65	0·78 (0·13–1·33)	0·14	0·78
Day 5	*Overall*	21·1 (6–91)	..	..	25·4 (4–71)	..	..	0·59 (0·13–1·38)	..	..
	AL	17·8 (6–48)	0·25	*Reference*	23·6 (12–46)	0·90	*Reference*	0·52 (0·13–1·00)	0·0027	*Reference*
	AL+PQ (0·25 mg/kg)	21·3 (6–49)	0·58	0.44	28·7 (7–54)	0·39	0·21	0·54 (0·21–1·18)	0·0091	0·69
	SPAQ	19·1 (7–48)	0·062	*Reference*	22·9 (4–39)	0·54	*Reference*	0·68 (0·14–1·38)	0·49	*Reference*
	SPAQ+TQ (1·66 mg/kg)	26·3 (8–91)	0·66	0·92	26·5 (7–71)	0·45	0·82	0·63 (0·13–1·17)	0·56	0·59
Day 7	*Overall*	20·1 (2–83)	..	..	26·0 (4–73)	..	..	0·67 (0·18–1·41)	..	..
	AL	15·2 (2–25)	0·71	*Reference*	24·8 (6–49)	0·53	*Reference*	0·56 (0·18–1·41)	0·033	*Reference*
	AL+PQ (0·25 mg/kg)	21·5 (3–40)	0·62	0·031	25·7 (4–55)	0·82	0·86	0·64 (0·24–1·05)	0·51	0·31
	SPAQ	20·2 (8–42)	0·008	*Reference*	23·9 (6–44)	0·31	*Reference*	0·78 (0·26–1·38)	0·019	*Reference*
	SPAQ+TQ (1·66 mg/kg)	23·5 (10–83)	0·53	0·13	29·9 (13–73)	0·58	0·64	0·68 (0·30–1·27)	0·76	0·30
Day 14	*Overall*	20·5 (7–50)	..	..	24·6 (8–56)	..	..	0·61 (0·17–1·45)	..	..
	AL	19·1 (9–49)	0·12	*Reference*	25·3 (8–56)	0·46	*Reference*	0·61 (0·17–1·38)	0·54	*Reference*
	AL+PQ (0·25 mg/kg)	21·7 (14–35)	0·62	0·38	26·5 (11–47)	0·90	0·92	0·58 (0·21–1·08)	0·048	0·81
	SPAQ	19·1 (8–49)	0·036	*Reference*	21·8 (9–38)	0·98	*Reference*	0·66 (0·28–1·45)	0·42	*Reference*
	SPAQ+TQ (1·66 mg/kg)	22·1 (7–50)	0·48	0·70	24·9 (10–45)	0·31	0·57	0·60 (0·17–0·97)	0·22	0·47

Alanine aminotransferase (ALT), aspartate aminotransferase (AST) and creatinine were compared within treatment arms (p-value[§]) using paired t-tests (with day 0 as reference) and between treatment arms (p-value[¶]) using linear regression (adjusted for baseline levels).
AL = artemether-lumefantrine; AL+PQ = artemether-lumefantrine with primaquine; SPAQ = sulfadoxine-pyrimethamine plus amodiaquine; SPAQ+TQ = sulfadoxine-pyrimethamine plus amodiaquine with tafenoquine.

References

1. Dicko A, Roh ME, Diawara H, et al. Efficacy and safety of primaquine and methylene blue for prevention of Plasmodium falciparum transmission in Mali: a phase 2, single-blind, randomised controlled trial. *Lancet Infect Dis* 2018; **18**(6): 627-39.
2. Dicko A, Brown JM, Diawara H, et al. Primaquine to reduce transmission of Plasmodium falciparum malaria in Mali: a single-blind, dose-ranging, adaptive randomised phase 2 trial. *Lancet Infect Dis* 2016; **16**(6): 674-84.
3. Rueangweerayut R, Bancone G, Harrell EJ, et al. Hemolytic Potential of Tafenoquine in Female Volunteers Heterozygous for Glucose-6-Phosphate Dehydrogenase (G6PD) Deficiency (G6PD Mahidol Variant) versus G6PD-Normal Volunteers. *Am J Trop Med Hyg* 2017; **97**(3): 702-11.
4. Meerstein-Kessel L, Andolina C, Carrio E, et al. A multiplex assay for the sensitive detection and quantification of male and female Plasmodium falciparum gametocytes. *Malar J* 2018; **17**(1): 441.
5. Bousema T, Okell L, Shekalaghe S, et al. Revisiting the circulation time of Plasmodium falciparum gametocytes: molecular detection methods to estimate the duration of gametocyte carriage and the effect of gametocytocidal drugs. *Malar J* 2010; **9**: 136.
6. Mendez F, Munoz A, Plowe CV. Use of area under the curve to characterize transmission potential after antimalarial treatment. *Am J Trop Med Hyg* 2006; **75**(4): 640-4.

NECTAR 3: A four-arm trial comparing artemether-lumefantrine with or without single-dose primaquine and sulphadoxine-pyrimethamine/amodiaquine with or without single-dose tafenoquine to reduce *P. falciparum* transmission in Mali

Version 2.0: 22 October 2021

Principal Investigators

Prof Alassane Dicko

Faculty of Pharmacy and Faculty of Medicine and Dentistry, University of Sciences Techniques and Technologies of Bamako, Mali

Prof. Dr. Chris Drakeley

London School of Hygiene & Tropical Medicine, United Kingdom

Co-investigators: Mali

Dr. Halimatou Diawara

Dr. Almahamoudou Mahamar

Dr. Harouna Soumare

Dr. Djibrilla Issiaka

Prof. Cheick Traore

Malaria Research and Training Centre, Faculty of Pharmacy and Faculty of Medicine and Dentistry, University of Sciences Techniques and Technologies of Bamako, Mali

Co-investigators: International

Prof. Teun Bousema

Radboud University of Nijmegen, The Netherlands

Dr. William Stone

London School of Hygiene & Tropical Medicine, United Kingdom

London School of Hygiene & Tropical Medicine is the main research sponsor for this study. For further information regarding the sponsorship conditions, please contact the Research Governance and Integrity Office:

London School of Hygiene & Tropical Medicine

Keppel Street

London WC1E 7HT

Tel: +44 207 927 2626

Email: RGIO@lshtm.ac.uk

Planned start date: *July 2021*

Planned end date: *July 2022*

Table of contents

Rationale 3
  Drug safety: Tafenoquine 5
  Statement of the problem 6
Objectives 7
  Primary specific objective 7
  Secondary specific objectives 7
Study site 7
Study design 8
Study population 9
Study drugs 9
  Dihydroartemisinin-Piperaquine (DP) 9
  Tafenoquine 10
Outcome measures 11
Sample size calculation 13
Inclusion Criteria 13
Exclusion criteria 14
Consent procedure 14
Study procedures 15
Randomization procedure 16
Blinding 16
Safety evaluation 16
  Adverse Event Data Collection 18
  Assessment of causality 19
  Assessment of outcome 19
  Data and Safety Monitoring Committee (DSMC) 20
  Treatment failure criteria 20
Laboratory procedures 20
  Blood film 20
  Mosquito infectivity assay 21
  Hemoglobin concentrations 21
  Complete blood count 21
  Biochemistry 21
Data management and analysis 21
Publication/Dissemination 23
Ethical issues 23

Subject information and consent 24
Confidentiality 24
Risks 24
Benefits 25
Compensation 25
Use and storage of study samples 25
Sponsor information........ 19
References 26
Appendix........ 23

Study synopsis

Artemisinin combination therapies (ACT) are the global first line for treatment of uncomplicated *P. falciparum* malaria. To contain the spread of artemisinin partially resistant *Plasmodium* strains and expedite efforts to achieve malaria elimination, the WHO recommends that ACT be combined with a single low dose of primaquine (SLD PQ) which has potent but short lived gametocytocidal activity. This drug combination clears the recipient's pathogenic parasites (ACT) and effectively annuls their infectivity to mosquitoes within 48 hours (PQ). Although artemether-lumefantrine is the most widely used ACT, most studies assessing PQ efficacy have been with dihydroartemisinin-piperaquine (DP); the added benefit of PQ when used in combination with AL, which has superior gametocytocidal activity to DP, is unknown. Tafenoquine (TQ) belongs to the same drug family as PQ (8-aminoquinolines) but has a longer duration of activity; its ability to block *P. falciparum* transmission have only recently been assessed and indicates potent but delayed activity. There are indications from unpublished trials that TQ may be better administered with non-ACTs, due to metabolic inhibition by artemisinin derivatives.

In this study, we aim to determine the transmission reducing efficacy of SLD PQ with Artemether Lumefantrine and SLD TQ with sulphadoxine-pyrimethamine and amodiaquine (SPAQ). To achieve this, we will conduct a four arm, single-blinded, randomized clinical trial. Individuals who meet inclusion criteria will be randomised in a 1:1:1:1 ratio to: AL, AL-PQ (0.25mg/kg), SPAQ, SPAQ-TQ (1.66mg/kg).

For participation in the trial, individuals between 10-50 years will be recruited from villages around Ouelessebougou, near Bamako in Mali. After obtaining the informed consent and assent (if applicable), participants will undergo a screening including clinical evaluation and for the presence of microscopically detectable *P. falciparum* infection. Individuals with ≥16 gametocytes/µL will be recruited and go through additional screening including haemoglobin measurement, G6PD deficiency, CBC, assessment of renal and liver functions, and pregnancy tests. Recruitment will continue until 80 infected G6PD normal individuals with microscopically detectable *P. falciparum* gametocytes (≥16/µL) are recruited and randomised.

Each participant will be followed for 28 days. Participants will receive a full clinical and parasitological examination on days 2, 5, 7, 14, 21, 28 after receiving the first dose of the study drugs. Blood samples will be taken at all visits for parasitology (microscopy, and whole blood for qRT-PCR) with additional samples for haematology and biochemistry analysis at days 0, 2, 5, 7 and 14. A clinical and haematological examination will also be performed on day 1, at which point no other samples will be collected.

Infectivity to locally reared mosquitoes will be assessed with membrane feeding assays at baseline, on the final day of treatment (day 2) and at days 5 and 7 for all participants. Feeds will then be performed at day 14 for any individuals who where infectious to mosquitoes at days 5 or 7; at day 21 for any individuals infectious at day 7 or 14; and at day 28 for any individuals infectious at day 14 or 21.

The primary transmission reduction outcome (percent reduction in mosquito infection rate) will be analysed within each arm compared to baseline, and secondary outcomes (i.e., prevalence of infectivity to any mosquitoes, oocyst density, gametocyte prevalence and density) will be analysed within and between specific (treatment matched) comparator arms.

Rationale

Efforts to reduce the global incidence of malaria are failing; there were an estimated 228 million cases of malaria worldwide in 2018, up from 214 million in 2015 (1). With the threat that this stagnation may be compounded by resistance to first-line drugs there is a clear need for interventions focused on reducing malaria parasite transmission (2).

Artemisinin combination therapies (ACTs) clear *P. falciparum* asexual parasites but lack activity against mature gametocytes (3). Gametocytes are the only *Plasmodium* life stage that can be transmitted to mosquitoes, so their prevalence and density define the malaria infectious reservoir. ACTs have different effects on post-treatment gametocyte carriage. A pooled analysis of microscopy data indicated that artemether-lumefantrine (AL) may be the most potent ACT in terms of gametocyte clearance whilst gametocyte persistence is markedly longer after the drug combination dihydroartemisinin-piperaquine (DP) (3). Importantly, post-treatment gametocyte carriage is a poor approximation of malaria transmission potential. Transmissible gametocytes may persist at submicroscopic densities after treatment (4) and some antimalarial drugs may sterilize gametocytes before these are removed from the circulation (5). Mosquito feeding experiments are needed to reliably determine post-treatment transmission potential and indicate considerable transmission post DP (6, 7). We recently demonstrated that the novel ACT pyronaridine-artesunate (PA; Pyramax®) has incomplete activity against gametocytes and allows similar levels of post-treatment transmission to DP (with 20-40% of individuals infecting mosquitoes at day 14, and some remaining infectious for up to 28 days) (8). Although AL accounts for more than 75% of all ACTs used worldwide, there is currently no reliable data on malaria transmission post-AL. The three studies that performed mosquito feeding assays post-AL reached very different conclusions. One suggested 14% of malaria patients may still have infectious gametocytes 7 days after AL (9), two others suggested negligible transmission after AL (3, 10). This is an important uncertainty: if there is substantial transmission after treatment with AL, the addition of a specific gametocytocide may be considered. In areas consolidating malaria elimination or aiming to contain the spread of artemisinin-resistance, the WHO recommends that ACTs be combined with a single-low dose of primaquine (SLD PQ/0.25mg/kg) (11).

PQ is an 8-aminoquinoline used principally to prevent *P. vivax* relapse, due to its activity against dormant hypnozoites. Complications of PQ include its toxicity in individuals with intermediate to low levels of the glucose-6 phosphate dehydrogenase (G6PD) enzyme, (which protects against the damaging haemolytic effects of oxidative compounds), and its short half-life; radical clearance requires repeat dosing (15mg daily for 2 weeks). As a *P. falciparum* gametocytocide, a single low dose (SLD) of 0.25mg/kg PQ causes an immediate and effective reduction in the transmission of parasites (6, 7) and is safe for use without prior G6PD status testing (12-14). We previously demonstrated that addition of single low doses PQ to DP and

PA results in a marked reduction in the duration of gametocyte carriage and transmission (6, 7, 15). It is currently unclear whether the addition of PQ to AL is beneficial or whether AL by itself already sufficiently prevents post-treatment transmission.

In addition, we recently demonstrated that single-dose tafenoquine at 1.66mg/kg (TQ) is safe and accelerates *P. falciparum* gametocyte clearance when combined with DP (Mahamar, Smit et al. 2021 *In preparation*). TQ has been proposed as long-lasting and safer alternative to PQ for preventing *P. vivax* relapse (16, 17) and, as a single dose up to 300mg, is safe in G6PD normal individuals (18-20). Our study confirmed that DP+TQ (1.66mg/kg) achieved total annulment of gametocyte infectivity within 7 days of treatment in Malian adults (Mahamar, Smit et al. 2021 *In preparation*). Though our first trial showed that TQ had significant gametocytocidal activity at doses of 1.66mg/kg (equivalent to 100mg total dose in adults), there are recent observations that DP may inhibit TQ activity against *P. vivax* hypnozoites; Indonesian soldiers given DP with or without 300mg TQ showed similar rates of relapse (21). Our data thus may not have reflected the full potential of TQs activity due to its sub-optimal partner drug. GlaxoSmithKline/CDC responded to the Indonesian data by changing their recommendation for *P. vivax* radical cure to treatment with the single 300mg dose of TQ and chloroquine only (22). Combination of TQ with non-ACTs for a *P. falciparum* gametocytocidal indication has not been tested.

Sulfadoxine-pyrimethamine (SP) with amodiaquine (AQ) is a non artemisinin based combined anti-malarial treatment (SPAQ) that is highly effective against *P. falciparum*. Currently SPAQ is the only antimalarial recommended for systematic mass administration in the form of seasonal malaria chemoprophylaxis (23). Prior studies show considerable post-treatment transmission potential following SPAQ: infectivity and mosquito infection rate may be unaffected for the first 7 days post SPAQ (6) with reductions in gametocytaemia only observed by 28 days (9). Increased gametocyte densities in the first 2 weeks following SPAQ (9) may limit the effect of SMC on transmission and the contribution to secondary infection, prompting assessments of SPAQ combined with SLD PQ (6). SP-AQ with SLD TQ has not yet been tested.

We propose to conduct a phase 2, single-blind, randomised control trial to test the gametocytocidal and transmission-blocking efficacy of single dose PQ in combination with AL and single dose TQ in combination SPAQ in Malian children and adults. During the conduct of this study, we will collect information on the carbon footprint of different study components to inform and support sustainable research practices.

Drug safety: Primaquine

Primaquine is known to cause dose-dependent haemolysis among individuals who are deficient in glucose-6-phosphate dehydrogenase (G6PD), an enzyme involved in glucose metabolism prevalent in malarious geographies (24). Haemolysis in G6PD-deficient (G6PDd) individuals is caused by oxidative stress induced by primaquine within red blood cells (RBCs); the extent of haemolysis depends on several factors including the dose of primaquine, the type of G6PD deficiency, the functional G6PD assessment at the time of dosing, the concurrent use of other oxidant drugs, and whether the person is hemizygous (homozygous) or heterozygous for G6PD-deficiency (24). In 2010 the WHO recommended the addition of a single low dose of primaquine (0.25 mg/kg) to standard ACT treatment, without mandatory G6PD testing to block *P. falciparum* transmission in areas threatened by artemisinin resistance or approaching malaria elimination (25, 26). The safety of single low dose PQ has subsequently been confirmed in clinical trials where PQ was combined with either DP or AL (12, 13, 15); two of these studies were specifically designed to assess safety in G6PD deficient individuals and found no clinically relevant reductions in haemoglobin concentrations (12, 13). PQ use is now

widespread throughout the world, and many countries have adopted the 0.25 mg/kg WHO recommendation for single low dose PQ without prior G6PD testing (27).

Drug safety: Tafenoquine

Peak plasma concentrations of TQ are reached 12 to 15 hours following oral administration. TQ has an average half-life of 16.5 days (range: 10.8 days to 27.3 days) in healthy adult subjects. The metabolism is slow with unchanged tafenoquine, the only notable drug-related component in human plasma after a single oral dose of tafenoquine (28). A single dose of 300mg TQ is safe and effective for radical treatment of *vivax* malaria in G6PD normal individuals (18, 19), and has equal haemolytic effects to a 14*15mg dose regimen of PQ in G6PD deficient females (29). Haemolytic effects in the of the 300mg single dose in this trial were minimal in G6PD normal individuals, and restricted to the first 14 days post treatment in G6PD deficient individuals (29). A single 50mg dose of TQ in combination with chloroquine (CQ) resulted in no clinically significant declines in haemoglobin compared with CQ alone (TQ+CQ: 1.5-2.5g/dL decrease in 13% [7/55]; CQ alone: 1.5-2.5g/dL decrease in 28% [15/55]) (19). 100mg single doses showed only small reductions in maximum haemoglobin decrease in G6PD deficient (heterozygous) female adults (29). We recently observed no clinically meaningful changes in haemoglobin concentration following administration of 1.66mg/kg (equivalent to 100mg in adults) as a single dose in Malian males and non-pregnant females over 12 years of age (Mahamar, Smit et al. 2021 *In preparation*). A full review of tafenoquine product and safety information, including our recent findings in the proposed study population, is presented in Appendix 1.

Though the risk of haemolytic adverse events is low, we will nonetheless take significant safety precautions including the performance of clinical examinations at all sampling time points, the offer of care free of charge to all participants throughout the duration of follow-up, and the monitoring of participant hemoglobin levels on each day of follow-up. Exclusion criteria for recruitment will include taking drugs which may be metabolized by cytochrome enzyme CYP2D6, blood transfusion in the last 90 days, known hypersensitivity to AL, PQ, SP, AQ or TQ, signs or symptoms of hepatic injury (such as nausea and/or abdominal pain Associated with jaundice) or known severe liver disease (i.e. decompensated cirrhosis, Child Pugh stage B or C), and signs or symptoms or known renal impairment. The minimum haemoglobin concentration at enrolment is ≥10 g/dL.

Statement of the problem

Artemisinin combination therapies (ACTs) rapidly clear asexual stage *P. falciparum* parasites, which account for most of the parasite's total biomass, but have no significant activity against transmissible gametocytes.

ACTs have variable effects on gametocyte clearance and transmission. For AL, the most widely used ACT, there is no robust data on its transmission-reducing effects. It is currently unknown whether adding single low dose PQ to AL accelerates its transmission-reducing potential. PQ can prevent transmission after several ACTs by rapidly sterilising and killing gametocytes. PQ's short half-life limits its use to the sterilisation of current infections only. TQ has been approved as an alternative to PQ for the treatment of *P. vivax* infections, was recently demonstrated to reduce transmission after ACT treatment and has far greater stability in the blood compared to PQ. Recent studies indicate that ACT may inhibit TQ activity, but TQ has not been combined with non-ACT for a *P. falciparum* gametocytocidal indication. SPAQ is used in SMC throughout the Sahel region: SPAQ with SLD PQ has been tested, but SPAQ with

TQ has not. We propose to conduct a phase 2, single-blind, randomised clinical trial, with the primary endpoint being the reduction in infectivity relative to baseline 2 or 7 days after treatment with i) AL alone; ii) AL with single-dose primaquine (0.25 mg/kg); iii) SPAQ alone; and iv) SPAQ with TQ (1.66 mg/kg). Infectivity and parasite metrics will be compared within arms, compared to baseline values, and between arms, to establish relative efficacy. Since we hypothesize faster gametocyte clearance in the AL arms compared to SPAQ arms, we will assess reductions in infectivity as primary endpoints on day 2 (AL, AL-PQ) and day 7 (SPAQ, SPAQ-TQ).

Objectives

Primary specific objective

- Assess the reduction of infectivity of gametocytes following administration of AL alone or with single dose PQ and SPAQ alone or with single-dose TQ in children and adults <u>without</u> G6PD deficiency at day 2 (AL, AL-PQ) and 7 (SPAQ, SPAQ-TQ) post-treatment compared to pre-treatment (day 0).

Secondary specific objectives

- Assess differences in other mosquito infectivity parameters (mosquito infection rate, change in infection rate, infectivity to mosquitoes, and oocyst density) following treatment with AL, AL-PQ, SPAQ & SPAQ-TQ in children and adults <u>without</u> G6PD deficiency at all feeding timepoints compared to pre-treatment (day 0) and between treatment matched arms (AL vs AL-PQ, SPAQ vs SPAQ-TQ).
- Assess differences in gametocyte parameters (prevalence, density, circulation time, area-under the curve, sex-ratio) following treatment with AL, AL-PQ, SPAQ & SPAQ-TQ in children and adults <u>without</u> G6PD deficiency compared to pre-treatment (day 0) and between treatment matched arms (AL vs AL-PQ, SPAQ vs SPAQ-TQ).
- Assess differences in safety parameters (AE frequency, Hb density, median drop in Hb) following treatment with AL, AL-PQ, SPAQ & SPAQ-TQ in children and adults <u>without</u> G6PD deficiency compared to pre-treatment (day 0) and between treatment matched arms (AL vs AL-PQ, SPAQ vs SPAQ-TQ).
- Assess differences in biochemical parameters (ALT, AST, CBC, Creatine, Methaemoglobin) following treatment with AL, AL-PQ, SPAQ & SPAQ-TQ in children and adults <u>without</u> G6PD deficiency compared to pre-treatment (day 0) and between treatment matched arms (AL vs AL-PQ, SPAQ vs SPAQ-TQ).

Exploratory objectives

- Assess parasite genomic and transcriptomic variation at baseline and at select post-treatment timepoints.
- Assess human genomic variation (i.e., HBB type) and association with parasite measures.
- Assess the impact of plasma biomarkers on malaria transmission efficiency.
- Estimate the carbon footprint of different components of the trial.

Study site

The Malaria Research and Training Centre (MRTC) in Bamako, Mali will use the field site of Ouelessebougou for recruitment. The MRTC has the unique position of being one of very few sites in Africa that can study malaria transmission endpoints. Since 2013 the MRTC successfully carried out similar trials at this site with great success, the University of California San Francisco, the London School of Hygiene and Tropical Medicine and the Radboud University Nijmegen, the Netherlands. In the 2013-2014 study, the MRTC demonstrated the efficacy of SLD PQ of doses of 0.125mg/kg or greater in combination with dihydroartemisinin-piperaquine for blocking *P. falciparum* transmission safely in G6PD normal Malian males from the age of 5-50 years (16, 17). MRTC has already established all the assays required for the study described in this protocol.

The MRTC is experienced in conducting Good Clinical Practice (GCP) compliant clinical trials including phase 1, 2, 3 and 4 of malaria vaccine and drug studies. Ouelessebougou village has been conducting ongoing clinical research since 2008 and is endemic for malaria with marked seasonality and a high burden of malaria in both children and adult populations. In recent years the prevalence of *P. falciparum* malaria in children under 5 years of age has ranged between 14% and 54% during the transmission season. The frequency of G6PD deficiency is in the range of 10 to 15%.

Study design

This is a four arm, single-blinded, randomized clinical trial. The study will be conducted according to the following steps for each individual:

Individuals aged 10-50 years will be invited to participate. They will be asked to provide informed consent and assent (for those under 18 years of age). After obtaining the informed consent and assent (if applicable), participants will undergo a screening including clinical evaluation and for the presence of microscopically detectable *P. falciparum* infection. Individuals with ≥16 gametocytes/µL will be recruited and go through additional screening including haemoglobin measurement, G6PD deficiency, CBC, assessment of renal and liver functions, and pregnancy tests. Recruitment will continue until 80 infected individuals with microscopically detectable *P. falciparum* gametocytes (≥16/µL) are recruited and randomised.

Individuals who met the inclusion and exclusion criteria will be randomised in a 1:1:1:1 ratio into four groups (arms); AL, AL-PQ (0.25mg/kg), SPAQ, SPAQ-TQ (1.66mg/kg). Randomisation will be performed by the study pharmacist. All other staff will be blinded to the treatment allocation.

Table 1. Treatment arms

Arm	ACT*	PQ (mg)	TQ (mg)	Sample size
1	AL	-	-	20
2	AL	0.25mg/kg	-	20
3	SPAQ	-	-	20
4	SPAQ	-	1.66mg/kg	20

Each participant will be followed for 28 days. Participants will receive a full clinical and parasitological examination on days 2, 7, 14, 21, 28 after receiving the first dose of the study drugs. Blood samples will be taken at all visits for parasitology (microscopy, and whole blood

for qRT-PCR) and serology with additional samples for haematology and biochemistry analysis at days 0, 2 and 14. A clinical and haematological examination will also be performed on day 1, at which point no other samples will be collected.

Infectivity to locally reared mosquitoes will be assessed with membrane feeding assays at baseline, on the final day of treatment (day 2) and at days 5 and 7 for all participants. Feeds will then be performed at day 14 for any individuals who where infectious to mosquitoes at days 5 or 7; at day 21 for any individuals infectious at day 7 or 14; and at day 28 for any individuals infectious at day 14 or 21.

In addition to this passive monitoring, participants will be prompted to contact the study coordinators if they feel sick at any point during follow up. A full clinical examination will be performed and treatment will be provided according to national treatment guidelines.

Study population

The study population will be derived from individuals aged 10-50 years with asymptomatic *P. falciparum* malaria infection who agree to be screened for malaria infection.

For the randomised trial, we will aim to recruit 80 individuals. Eligible individuals will have microscopically detectable *P. falciparum* gametocyte densities of ≥16 gametocytes/µL.

Study drugs

Artemether-lumefantrine (AL)

Participants in the AL or AL+PQ arm will be treated with standard doses of AL (Coartem, Novartis, Basel, Switzerland). Tablets containing 20/80 mg artemether and 120/480 mg lumefantrine will be administered per manufacturer guidelines.

Table 2. *AL dosing*

**Bodyweight (kg)**	**20/120 mg tablet**			**80/480 mg tablet**		
	D1	D2	D3	D1	D2	D3
5 to < 15 kg	1 disp tab x 2	1 disp tab x 2	1 disp tab x 2	–	–	–
15 to < 25 kg	2 disp tab x 2	2 disp tab x 2	2 disp tab x 2	–	–	–
25 to < 35 kg	3 tab x 2	3 tab x 2	3 tab x 2	–	–	–
≥ 35 kg	4 tab x 2	4 tab x 2	4 tab x 2	1 tab x 2	1 tab x 2	1 tab x 2

Primaquine (PQ)

Participants in the AL-PQ arm will receive PQ (ACE Pharmaceuticals, Zeewolde, The Netherlands)) at a single low dose of 0.25mg/kg as is currently recommended by the World Health Organization. The single dose of PQ will be given on day 0 together with AL, administered in an aqueous solution, according to a standard operating procedure (SOP) provided by Sanofi as previously done at the study site when PQ was combined with DP or SPAQ (6, 7).

Sulphadoxine-pyrimethamine with amodiaquine (SPAQ)

Participants in the SPAQ or SPAQ-TQ arm will be treated with standard doses of SPAQ (Guilin Pharmaceutical (Shanghai) Co., Ltd. Rm. 701, 780 Cailun Rd., Zhangjiang Hi Tech Park, Shanghai, 201203 China). Amodiaquine tablets contain 150mg amodiaquine (as hydrochloride). Sulfadoxine/pyrimethamine tablets contain 500mg sulfadoxine and 25mg pyrimethamine. Both medicines were manufactured by Guilin Pharmaceutical Co, Shanghai, China. Tablets will be administered per manufacturer guidelines shown below:

***Table 3. A.** SP dosing **B.** AQ dosing*

A.

**Bodyweight (kg)**	**SP: 500/25mg tablet**		
	D1	D2	D3
11 to 20 kg	1	1	1
21 to 30 kg	1.5	1.5	1.5
31 to 45 kg	2	2	2
> 45 kg	3	3	3

B.

**Bodyweight (kg)**	**AQ: 150mg tablet**		
	D1	D2	D3
15 to 18 kg	1.5	1	1
19 to 24 kg	1.5	1.5	1.5
25 to 35 kg	2.5	2.5	2
36 to 50 kg	3	3	3
> 50 kg	4	4	3

Tafenoquine

A single dose of TQ (60degrees Pharma, Washington, US) will be given on day 0 immediately following the first dose of SPAQ at 1.66mg/kg. TQ dosing will be weight based in order to standardise efficacy and risk variance. The maximum dose chosen for the current study is based on equivalent safety profile of TQ doses ≤300mg and to standard PQ dosing (15mg daily for 14 days) in adult G6PD heterozygous adult individuals (29). In the latter study, subjects were healthy females aged 18-45 years.

100mg Tafenoquine tablets are available for this study, and will be prepared into a 1mg/mL solution in water for weight-based dosing in 5 kg bands as follows:

***Table 4.** TQ dosing*

Weight min	Weight max	TQ 1mg/mL total (mL)	Water (mL)	Masking solution (mL)
30	35	53.95	136.05	10
35.01	40	62.2583	127.7417	10
40.01	45	70.5583	119.4417	10
45.01	50	78.8583	111.1417	10
50.01	55	87.1583	102.8417	10
55.01	60	95.4583	94.5417	10
60.01	65	103.7583	86.2417	10
65.01	70	112.0583	77.9417	10
70.01	75	120.3583	69.6417	10
75.01	80	128.6583	61.3417	10

Intervention

Individuals who meet the inclusion criteria in the current study will be randomised in a 1:1:1:1 ratio into four groups (arms); AL, AL-PQ, SPAQ & SPAQ-TQ. Randomisation will be performed by the study pharmacist. All staff other than the study pharmacist and clinician will be blinded to the treatment allocation. Participants will be told prior to enrollment that they can ask at any time which treatment arm they are in: The clinician or pharmacist will discuss this with them.

Outcome measures

Mosquito infectivity will be assessed at three levels: the mean number of oocysts in a sample of mosquitoes (i.e., oocyst intensity), the proportion of mosquitoes in a given assay infected with any number of oocysts (i.e., mosquito infection rate), and the infectivity of the study participant to any number of mosquitoes (i.e. infectivity to mosquitoes). All analyses of mosquito infection measures will be conducted separately for all enrolled individuals, and all individuals infectious at baseline – primary and secondary analyses will be performed on individuals infectious at baseline.

Our study will be powered to determine the transmission blocking efficacy of the trial treatments within each arm by comparison of pre- and post-treatment mosquito infection measures. The study is not designed to compare the transmission-blocking effects between treatment arms; comparison of outcomes between arms will be secondary and will only be undertaken for treatment matching arms (AL vs AL-PQ, SPAQ vs SPAQ-TQ).

Primary outcome measure:

- Mean within person percent change (presented as percent reduction) in mosquito infection rate in infectious individuals from baseline (day 0, pre-treatment) to day 2 post treatment in the AL and AL-PQ arms, and day 7 post-treatment in the SPAQ and SPAQ-TQ.

Secondary outcome measures:

- Mean within person percent change (presented as percent reduction) in mosquito infection rate from baseline to all feeding time-points, with comparison within and between arms.
- Mean mosquito infection rate at all feeding time-points, with comparison within treatment arms compared to baseline, and between arms.
- Infectivity to mosquitoes at all feeding time-points, with comparison within treatment arms compared to baseline, and between arms.
- Mean oocyst intensity (in all/all infected mosquitoes) at all feeding time-points, with comparison within treatment arms compared to baseline, and between arms.
- Male and female gametocyte prevalence at all time-points, determined by microscopy or molecular assays, with comparison within treatment arms compared to baseline, and between arms.
- Male and female gametocyte density at all time-points, determined by microscopy or molecular assays, with comparison within treatment arms compared to baseline, and between arms.
- Male and female gametocyte sex ratio (proportion male) at all time-points, determined by microscopy or molecular assays, with comparison within treatment arms compared to baseline, and between arms.
- Gametocyte circulation time (cumulative), determined by microscopy or molecular assays, compared between treatment arms.
- Gametocyte area under the curve (cumulative), determined by microscopy or molecular assays, compared between treatment arms.

- Asexual and total parasite prevalence at all time-points, determined by microscopy or molecular assays, with comparison within treatment arms compared to baseline, and between arms.
- Asexual and total parasite density at all time-points, determined by microscopy or molecular assays, with comparison within treatment arms compared to baseline, and between arms.
- Haemoglobin density at all time-points, with comparison within treatment arms compared to baseline, and between arms.
- Median within person percent change (presented as percent reduction) in haemoglobin density from baseline to all time-points, with comparison within and between arms.
- Methaemoglobin density at all time-points, with comparison within treatment arms compared to baseline, and between arms.
- Median within person percent change (presented as percent reduction) in methaemoglobin density from baseline to all time-points, with comparison within and between arms.
- The frequency and prevalence of adverse events (all AE's, treatment related AE's, and haematological AE's) observed up to and including day 2, 7, and 14 post-treatment, and at all timepoints.

Exploratory outcomes

- Parasite genotype and transcriptional analysis at baseline and at post-treatment timepoints.
- Plasma biomarkers (antibodies and parasite protein) at baseline and at post-treatment timepoints.
- Human genotype analysis at baseline (G6PD, CYP2D6, HBB)
- ALT/AST/Creatine density at all time-points with comparison within treatment arms compared to baseline, and between arms.

Throughout the study, we will keep track of the impact of the activities in terms of electricity consumption and environmental footprint.

Table 5. Outcome measures

Outcome measure	Day 0	Day 1	Day 2	Day 5	Day 7	Day 14	Day 21	Day 28
Mosquito infectivity	*		*	*	*	(*)	(*)	(*)
Asexual parasite prevalence & density	*		*	*	*	*	*	*
Gametocyte prevalence, density, sex ratio, genotype	*		*	*	*	*	*	*
Human genotyping	*							
Biochemistry (ALT/ AST Creatinine)	*		*	*		*		
CBC	*		*	*		*		
Haemoglobin/Methaemoglobin	*	*	*	*	*	*	*	*
Safety assessment: signs of haemolysis	*	*	*	*	*	*	*	*
Safety assessment: Adverse Events	*	*	*	*	*	*	*	*
Plasma biomarkers	*		*	*	*	*	*	*

Infectivity will be assessed at day 14 only for any individuals who where infectious to mosquitoes at days 5 or 7; at day 21 these outcome measures are assessed for any individuals infectious at day 7 or 14; and at day 28 for any individuals infectious at day 14 or 21.

Sample size calculation

For sample size considerations, we estimated the infectivity for participants in previous trials in the same study setting (6-8) using a mixed effects logistic regression model that accounted for correlation between mosquito observations from the same participant. When including 115 participants, including those non-infectious at baseline, pre-treatment infectivity of an average participant was 8% (i.e. 8% infected mosquitoes at baseline) with a standard deviation of an assumed normally distributed random intercept of 1.8. We then used this mixed-effects logistic regression model, that included random effects to allow within-person calculations of reductions in infectivity after treatment, to simulate data and calculate empirical power to detect reductions in infectivity after treatment. With an expected reduction in infectivity of 90% (as previously detected for efficacious doses of PQ and TQ (6-8), we calculated 92% empirical power to detect > 85% reduction in infectivity with a one-tailed test with an α=0.05 level of significance when including 20 participants and dissecting 50 mosquitos at each time point. When using an α=0.025, empirical power was calculated at 90%.

Inclusion Criteria

- Age ≥ 10 years and ≤ 50 years
- G6PD-normal defined by Carestart rapid diagnostic test or the OSMMR2000 G6PD qualitative test
- Absence of symptomatic falciparum malaria, defined by fever on enrolment
- Presence of *P. falciparum* gametocytes on thick blood film at a density >16 gametocytes/µL (i.e. ≥ gametocytes recorded in the thick film against 500 white blood cells)

- Absence of other non-*P. falciparum* species on blood film
- Hemoglobin ≥ 10 g/dL
- Individuals weighing < = 80 kg
- No evidence of acute severe or chronic disease
- Written, informed consent

Exclusion criteria

- Women who are pregnant or lactating (tested at baseline). Urine and/or serum pregnancy testing (β-hCG) will be used.
- Detection of a non-*P. falciparum* species by microscopy
- Previous reaction to study drugs / known allergy to study drugs
- Signs of severe malaria, including hyperparasitemia (defined as asexual parasitemia > 100,000 parasites / µL)
- Signs of acute or chronic illness, including hepatitis
- The use of other medication (except for paracetamol and/or aspirin)
- Use of antimalarial drugs over the past 7 days (as reported by the participant)
- The use of other medication (except for paracetamol and/or aspirin)
- Clinically significant illness (intercurrent illness e.g., pneumonia, pre-existing condition e.g., renal disease, malignancy or conditions that may affect absorption of study medication e.g., severe diarrhea or any signs of malnutrition as defined clinically)
- Signs of hepatic injury (such as nausea and/or abdominal pain associated with jaundice) or known severe liver disease (i.e., decompensated cirrhosis, Child Pugh stage B or C)
- Signs, symptoms or known renal impairment
- Clinically significant abnormal laboratory values as determined by history, physical examination or routine blood chemistries and hematology values (laboratory guideline values for exclusion are hemoglobin < 10 g/dL, platelets < 50,000/µl, White Blood Cell count (WBC) < 2000/µl, serum creatinine >2.0mg/dL, or ALT or AST more than 3 times the upper limit of normal for age.
- Blood transfusion in the last 90 days.
- Consistent with the long half-life of tafenoquine, effective contraception should be continued for 5 half-lives (3 months) after the end of treatment.
- History of psychiatric disorders

Consent procedure

Consenting procedures will vary based on age of the potential participant. Participants aged 18 years and above will provide informed consent. For participants under 18 years of age we will seek parental consent. In addition to parental consent, assent will be sought for children aged 10-17 years. The Ethics committee in Mali does not require that participants under the age of

10 provide assent. The written, informed consent procedure will be conducted in French or in a local language understood by subject as described in the information and consent form. If the participant is unable to read or write, a fingerprint will be used as an official signature.

Study procedures

Participants who agree to participate and provide written, informed consent will be assessed for the presence of sexual blood stage parasite with a thick blood film. Individuals with ≥16 gametocytes/µL will go through additional screening for eligibility including haemoglobin measurement, G6PD deficiency test using G6PD test (Carestart™ 3 G6PD, Access Bio, USA or OSMMR-2000 G6PD test, R&D Diagnostics Ltd ®, Greece), CBC, assessment of the renal and liver functions and pregnancy tests. Recruitment will continue until 80 infected individuals with microscopically detectable *P. falciparum* gametocytes (≥16/µL) are recruited and randomised. Individuals with symptomatic malaria will not be enrolled in the study and will be treated with artemether + lumefantrine, the standard treatment for uncomplicated malaria as per the Ministry of Health policy in Mali. Individuals screened found with other acute diseases will also be treated according to the standard of care in Mali. Those with chronic disease will be referred to the district hospital for further evaluation and management.

Eligible patients will then be randomized (see below under randomization procedure) to one of the treatment groups, and drugs will be administered. In case of vomiting within 30 minutes after drug intake, the drugs will be re-administered once.

The participants will be compensated for any travel costs and for work loss income. Participants will be followed for 28 days as described in Table 3 (below). Single venous draws or finger pricks will be used.

Any individuals determined to have an adverse event (haemolytic or otherwise) will be treated in accordance with national guidelines and best practice, and will be withdrawn from the study at the discretion of the study clinician.

Table 6. Sampling framework for study participants

Required vacutainer	Time of sampling (d = day)	Required volume (mL)	Day 0	Day 1	Day 2	Day 5	Day 7	Day 14	Day 21	Day 28
	Type of sampling$^{\$}$		V	C	V	V	V	V	V/C	V/C
Heparin (2mL)	Mosquito infectivity	2	*		*	*	*	**	**	**
EDTA (1.5-3mL)	Blood in RNA protect for RNA analysis/parasite genotyping	0.1-0.6	*		*	*	*	*	*	*
	Blood smear for asexual parasite and gametocyte density	0.1	*		*	*	*	*	*	*
	Filter paper blood spots	0.05	*		*	*	*	*	*	*
	Haemoglobin	0.05	*	*	*	*	*	*	*	*
	G6PD activity	0.5	*							
	CBC	0.5	*		*	*	*	*		

	Human genotyping	1	*							
SST (1mL)	Biochemistry	0.75	*		*	*	*	*		
	**Maximum total Volume of blood sampled (mL)**	**24.9-33.3**	**6**	**0.3**	**4.5**	**4.5**	**4.5**	**4.5**	**0.3/4.5**	**0.3/4.5**

$ V denotes venous blood sampling and C denotes capillary sampling from a finger prick.

*** Mosquito feeding will only occur from day 14 onwards if the participant in question infected mosquitoes at either of the previous two timepoints. Blood draw may involve either venipuncture or capillary sampling (not both). Max. blood volume varies for this reason.*

Randomization procedure

Eligible participants will be assigned the next sequentially numbered study ID number based on a pre-printed Study ID List created by a study investigator. Randomization will be computer generated using a 1:1:1:1 ratio. The randomisation codes will be provided in opaque sealed envelope to the study pharmacist. The study pharmacist in Mali will open the corresponding sealed, opaque envelope and provide the intervention to study participants. Randomisation will be performed in blocks of 12.

Blinding

This is a single blind randomised controlled trial. The treating physician and staff involved with assessing all laboratory outcomes of the study are blinded, but no placebo will be used. The study pharmacist will be unblinded and responsible for randomisation and treatment administration. Entomology staff involved in the mosquito feeding assays will be blinded for the parasitology results.

Safety evaluation

The major safety endpoint is haemolysis. For this reason, hemoglobin will be measured before treatment, at days 1, 2, 5, 7, 14, 21, and 28. Previous studies have shown that highest hemoglobin fall related to drug induced haemolysis can be detected within 7 days after treatment. In addition at 24 hours, and subsequent days of follow up, a questionnaire assessing adverse events (AE) will be carried out. All participants will have access to contact study medical staff 24 hours a day and medical facilities that can give safe blood transfusions. Participants who require blood transfusions will be hospitalised at the district hospital in Ouelessebougou or referred to the University hospitals in Bamako for proper management. Additional safety precautions due to the small risk of transient haemolysis in this population will include the performance of clinical examinations and monitoring for adverse events (AE) at all sampling time points, care free of charge to all participants throughout the duration of follow-up.

- ***Adverse events***

  Adverse events are defined as any undesirable experience occurring to a subject during the study, whether or not considered related to trial. An AE can therefore be any unfavourable and unintended sign (including an abnormal laboratory finding), symptom, or disease (new or exacerbated) temporally associated with a study

intervention. AEs may include events that occur as a result of protocol-mandated procedures (i.e. invasive procedures). All AEs reported spontaneously by the subject or observed by the trial clinicians or their staff will be recorded. Abnormal laboratory findings or other abnormal assessments that are judged by the trial clinicians to be clinically significant will be recorded as AEs or serious adverse events (SAEs) if they meet the definition. The investigators and trial clinicians will exercise their medical and scientific judgement in deciding whether an abnormal laboratory finding or other abnormal assessment is clinically significant.

- ***Serious adverse events***

  A serious adverse event is any untoward medical occurrence or effect that at any dose:

  - Results in death
  - Is life threatening (at the time of the event)
  - Requires hospitalisation
  - Results in persistent or significant disability or incapacity
  - Congenital anomaly/birth defect
  - Medically important event

  All SAEs will be reported by the principal investigators to the Ethics Committees and DSMB within 48 hours of awareness of the PI.

- ***Unexpected Adverse Event***

  An AE is considered unexpected if it is not listed in the Investigator's Brochure (IB) or Package Insert (for marketed products) or is not listed at the specificity or severity that has been observed.

- ***Serious and Unexpected Suspected Adverse Reaction (SUSAR)***

  A SUSAR is a SAR that is both serious and unexpected.

- ***Unanticipated Problem (UP*)**

  An UP is any event, incident, experience, or outcome that is

  1. unexpected in terms of nature, severity, or frequency in relation to
     a. the research risks that are described in the approved research protocol and informed consent document, IB, or other study documents; and
     b. the characteristics of the subject population being studied; and
  2. possibly, probably, or definitely related to participation in the research; and
  3. places subjects or others at a greater risk of harm (including physical, psychological, economic, or social harm) than was previously known or recognized.

- ***Serious Unanticipated Problem (UP)***

A UP that meets the definition of a Serious Adverse Event or compromises the safety, welfare or rights of subjects or others.

- ***Unanticipated Problem that is not an Adverse Event (UPnonAE)***

An UPnonAE is an UP that does not fit the definition of an adverse event, but which may, in the opinion of the investigator, involve risk to the subject, affect others in the research study, or significantly impact the integrity of research data. Such events would be considered a non-serious UP. For example, we will report occurrences of breaches of confidentiality, accidental destruction of study records, or unaccounted-for study drug.

All UPs that are also adverse events will be reported to the sponsor no later than 7 calendar days of site awareness of the event. UPs that are not AEs will also be reported to the sponsor.

Adverse Event Data Collection

Safety assessments will be performed and recorded by the trial clinicians. All AEs/reactions, observed by the trial clinicians or by the subject, will be accurately documented in the case report form. For each event/reaction the following details will be recorded:

1. Description of the event(s)/reaction(s)
2. Date and time of occurrence
3. Duration
4. Intensity
5. Relationship with the intervention
6. Action taken, including treatment
7. Outcome

In addition, symptoms will be ranked as (1) mild, (2) moderate, or (3) severe, depending on their intensity. All AEs except fever will be judged for their intensity according to the following scale:

- Mild (grade 1): awareness of symptoms that are easily tolerated and do not interfere with usual daily activity
- Moderate (grade 2): discomfort that interferes with or limits usual daily activity
- Severe (grade 3): disabling, with subsequent inability to perform usual daily activity, resulting in absence or required bed rest

For fever, the following scale will be used:

- Mild (grade 1): 37.5 - 38.0°C
- Moderate (grade 2): > 38.0 to 39.0°C
- Severe (grade 3): > 39.0°C

When an AE/SAE occurs, it is the responsibility of the principal investigators and trial clinicians to review all documentation (e.g. hospital progress notes, laboratory, and diagnostics reports) related to the event. The principal investigators and trial clinicians will then record all relevant information regarding an AE/SAE on the CRF or SAE Report Form, respectively. Furthermore, the trial clinicians will attempt to establish a diagnosis of the event based on signs, symptoms, and/or other clinical information. In such cases, the diagnosis should be documented as the AE/SAE and not the individual signs/symptoms.

Haematological severe AE

A haematological severe AE is defined as a drop in Hb density of >40% from baseline. These will be reported separately in the data safety and monitoring reports and in any resulting publications, due to their potential linkage with PQ or TQ treatment.

Assessment of causality

The principal investigators and trial clinicians are obligated to assess the relationship between study procedures and the occurrence of each AE/SAE. The clinicians will use clinical judgment to determine the relationship. Alternative causes, such as natural history of the underlying diseases, concomitant therapy, other risk factors and the temporal relationship of the event to the study procedures will be considered and investigated. The relationship of the AE with the study procedures will be assessed considering the factors listed under the following categories:

Table 7. Causality

**Definitely Related**	• reasonable temporal relationship<br>• follows a known response pattern<br>• clear evidence to suggest a causal relationship<br>• there is no alternative aetiology
**Probably Related**	• reasonable temporal relationship<br>• follows a suspected response pattern (based on similar agents)<br>• no evidence of a more likely alternative aetiology
**Possibly Related**	• reasonable temporal relationship<br>• little evidence for a more likely alternative aetiology
**Unlikely Related**	• does not have a reasonable temporal relationship<br>OR<br>• good evidence for a more likely alternative aetiology
**Not Related**	• does not have a temporal relationship<br>OR<br>• definitely due to an alternative aetiology

Note: Other factors will also be considered for each causality category when appropriate. Causality assessment is based on available information at the time of the assessment of the AE. The investigator may revise the causality assessment as additional information becomes available.

Assessment of outcome

Assessment of each AE must be assessed according to the following classification:

Table 8: Outcome

**Completely recovered**	The subject has fully recovered with no observable residual effects
**Not yet completely recovered**	The subject condition has improved, but still has some residual effects
**Deterioration**	The subject's overall condition has worsened
**Permanent damage**	The AE has resulted in a permanent impairment

**Death**	The subject died due to the AE
**Ongoing**	The AE remains the same as at onset
**Unknown**	The outcome of the AE is not known because of lost to follow-up

All adverse events will be recorded on individual CRF with the following information: the severity grade (mild, moderate, severe), its relationship to the study drug(s) (related/not related), its duration (start and end dates or if continuing at final exam), actions taken, outcome, whether it constitutes a serious adverse event (SAE).

Data and Safety Monitoring Committee (DSMC)

A Data and Safety Monitoring Committee (DSMC) will be established and convened before the onset of the trial; an independent local safety monitor (LSM) will be appointed in Mali and form part of the DSMC. The DSMC will convene meetings in person or by telephone conference prior to the study initiation, after the first 40 subjects have been enrolled and upon completion of the study. In addition to these three scheduled meetings, *ad hoc* meetings may be organised based on monthly progress reports that are provided to the DSMC. These monthly reports include an update on the number of participants who were enrolled, who completed the study and who discontinued the study (with reason provided). In addition, key safety data are provided: AEs and up to day 14 haemoglobin concentrations. Any SAEs or Suspected Unexpected Serious Adverse Reactions (SUSARs) are first reported and discussed among the research team and LSM and subsequently reported within 48 hours to the complete DSMC. If an SAE or SUSAR is deemed possibly, probably or definitely related to the study medication by the DSMB, the trial will be stopped until the DSMB has decided how to proceed or to stop the study.

Treatment failure criteria

In case a patient develops symptomatic malaria (denoted as fever and the presence of asexual parasitemia denoted by microscopy) at any time during followup, he will be treated with a full course of artemether-lumefantrine (Coartem, Novartis), the first-line antimalarial in Mali. Dosing will be as per the manufacturers guidelines as indicated in table 2.

Laboratory procedures

Blood film

Thick and/or thin blood films for parasite counts will be obtained and examined at screening to confirm *P. falciparum* monoinfection. Giemsa-stained thick and/or thin blood films will be examined at a magnification of 100×. The blood smear will be considered negative if no parasites are seen after examining 100 high powered fields.

Gametocyte and asexual stage density measurement

Blood slides stained with Giemsa will be double read over 500 fields for quantification of gametocytes and asexual stages. EDTA samples of blood (200 µL) will be tested for molecular quantification of gametocytes and asexual parasites. Parasite DNA and RNA will be extracted from whole blood samples in EDTA tubes and tested using quantitative real-time polymerase chain reaction (qPCR) with a detection limit of 0.02-0.1 parasites/µL of blood and highly precise parasite quantification (41). Quantification of gametocytes will be based on sex specific female (Pfs25/CCP4) and male (PfMGET) mRNA detection and quantification by reverse

transcriptase-PCR (11). Markers of asexual stage parasites will include 18s Ribosmal and SBP1 mRNA. Markers of sexual commitment will also be assessed (e.g. Ap2-g).

Further molecular analysis of samples from individuals who have undergone feeding assays (either from whole blood samples or from mosquito midguts) will include parasite genotyping (e.g. to assess clonal complexity or determine the presence of specific deletions e.g. HRP2) and human genotyping for specific genes (HBB, CYP2D6, G6PD).

Serology and biomarkers

Serological analysis of these samples will include assessment of antibody responses to gametocyte and asexual stage proteins in the microarray platform (38). Non antibody protein biomarker assessment will be conducted using bead based assays to assess for correlates of infection and infectivity (i.e. proteins associated with inflammation), or parasite density (HRP2). If serum remains after mosquito feeding, some may be stored and used in standard membrane feeding assays in Radboud University, Nijmegen, the Netherlands, for the assessment of functional transmission reducing immunity (42).

Mosquito infectivity assay

For each assessment of infectivity 2 ml of heparinized blood will be drawn from the study participant and stored at 37°C and transported to the insectary. At the insectary, using standard procedures, ~70 *A. gambiae* will be fed on the subjects' blood for 15-20 minutes (this figure is based on a previous study where 61 fed mosquitoes allowed for an average of 50 mosquitoes surviving until day 7 after the feeding experiment and being available for dissection and examination for oocysts). All of these mosquitoes will be dissected on the 7th day after the feeding assay for prevalence of mosquitoes with oocysts and quantification of oocysts. Infected guts will be stored for later PCR confirmation of oocysts.

Haemoglobin concentrations

Haemoglobin concentrations will be also measured regularly throughout the study using the HemoCue system (Hemocue AB, Angelholm, Sweden).

Methaemoglobin concentrations

MetHb will be measured non-invasively using a Masimo Rainbow Pulse CO-Oximeter (Masimo, Neuchâtel Switzerland).

Complete blood count

Using standard techniques, the clinical laboratory will perform the complete blood count (CBC) and platelet count. The following CBC parameters will be evaluated for exclusion criteria at baseline and for safety assessment during the study on days 2, 7 and 14: white blood cells, haemoglobin and platelets.

Biochemistry

Blood chemistries values will be also measured throughout the study. The following parameters will be evaluated for exclusion criteria at baseline and for safety assessment during the study on days 2, 7 and 14: serum creatinine, or ALT or AST.

Collection of data on the environmental footprint of activities

A formal Life Cycle Assessment (LCA) will be performed. This includes compiling inventories of the environmentally relevant flows (i.e., emissions, natural resources, material and energy, waste) related to all processes involved in the production of consumables, data collection,

sample storage and sample processing. LCA will quantitatively assess environmental impacts of goods and processes from ‘cradle to grave’. The goal of this analysis is to determine key drivers of environmental impact to identify what drivers for impact can be identified with the largest reduction potential (30). The LCA will include, for example, fuel consumption of project vehicles, electricity consumption of freezers and (international) travel of project staff and collaborators. All data will use CO_2equivalent (CO_2e) emissions as unit, translating other emissions (e.g. CH_4) and processes into CO_2e. For data processing and virtual meetings, we will assume CO_2e emission intensity for electricity generation (240 gCO_2e kWh^{-1})(31). We will calculate the contribution of network-related emissions, laptop emissions and server emissions:

i) Network-related emissions: the number hours online × 1.2 Mbps × 3,600 s h^{-1} × 1/8 byte bit^{-1} × 1/1,024 GB MB^{-1} × 0.06 kWh GB^{-1} × 0.24 kg kWh^{-1} = outcome in kgCO_2e
ii) Laptop-related emissions: the number of hours online × 30 W × 1/1,000 kW W^{-1} × 0.24 kg kWh^{-1} = outcome in kgCO_2e
iii) Server related emissions: the number of meeting hours × 300 W × 1/1,000 kW W^{-1} × 0.24 kg kWh^{-1} = outcome in kgCO_2e

Similar calculations will be used for electricity consumption of, for example, laboratory equipment and air-conditioning. International travel and shipment of goods will use an approach where the distance of each flight leg was translated to CO_2 equivalent emissions (CO_2e) using mean emissions and emission factors per km and per passenger as described before (32) using the mean of three independent estimates and taking into account i) direct emission of radiatively active substances (e.g. CO_2); ii) emission of chemical species that alter radiatively active substances; iii) emission of substances that trigger generation of aerosol particles or change natural clouds (33).

Lab consumables will similarly be included in the LCA, using resources of the Laboratory Efficiency Assessment Framework that allow quantification of CO_2e emissions of laboratory processes and reagents (34).

Data management and analysis

All data will be stored on secure password protected databases. Data that are collected on paper forms will be double entered. Data collected through handheld devices or directly produced by laboratory equipment will be examined for quality assurance and fed into the database using double data entry. Handheld devices used for data input and temporary storage will be password protected and data will be encrypted.

Analysis plan

Mosquito infectivity will be assessed at three levels: the mean number of oocysts in a sample of mosquitoes (i.e. oocyst intensity), the proportion of mosquitoes infected with any number of oocysts (i.e. mosquito infection rate), and the infectivity of the study participant to any number of mosquitoes (i.e. infectivity to mosquitoes). The primary outcome measure will be the percent change in mosquito infection rate within each arm by day 2 (AL & AL-PQ arms) or day 7 (SPAQ & SPAQ-TQ arms) compared to baseline, with other time-points of infectivity assessments as secondary outcomes. Percent change will be reported as percent reduction (with 100% as total reduction of transmission, and negative values as enhanced transmission). Other secondary transmission outcomes are as described in the ‘Objectives’ section above. Some exploratory objectives may be published separately from the main primary/secondary outcomes.

Statistical analysis will be conducted using the most recent STATA and SAS versions (16.0 and SAS version 9.4 at time of writing). Mosquito infection data will be analysed at time-points after baseline only for those individuals who are infectious at baseline, although the data and analyses from all enrolled individuals will be published in the appendix for completeness. Individuals will be classified as infectious to mosquitoes if they infect at least 1 mosquito, with any number of oocysts. Infectivity to mosquitoes and parasite/gametocyte prevalence will be compared within and between treatment arms using generalised linear models (family: binomial, z-score, co-efficient with 95% CI) or fishers exact tests. Absolute haemoglobin density and percent change in haemoglobin density (relative to baseline) will be compared using paired t-tests (t-score for difference compared to day 0) and two-way t-tests (t-score for difference between ACT matched treatment groups at each time-point). The proportion of gametocytes that are male will be calculated for all values with total gametocyte densities of 0.2/µL and over (9). Gametocyte circulation time will be calculated to determine the average period (in days) that a mature gametocyte circulates in the blood prior to clearance, using a deterministic compartmental model that assumes a constant rate of clearance and has a random effect to account for repeated measures on individuals, as described previously (35); circulation time will be analysed using t-tests (t score for difference between ACT matched treatment groups) or linear regression analyses. Area under the curve (AUC) of gametocyte density per participant over time will be calculated using the linear trapezoid method (36) using the first 28 days of observation only, and analysed by fitting linear regression models to the log10 adjusted AUC values, with and without adjustment for baseline gametocyte density (t-score, coefficient with 95% CI). All other analyses of quantitative data will be performed using Wilcoxon sign rank tests (z-score for difference compared to matched values at day 0) and Wilcoxon rank-sum tests (z-score for difference between ACT matched treatment arms at each time-point). For all analyses, the threshold for statistical significance will be set at $p<0.05$.

Publication/Dissemination

Findings will be analysed and presented to local researchers and stakeholders in Mali and at other research meetings and symposium. Data will be submitted for publication in peer reviewed journals in due course. Anonymized data may be shared in public repositories upon publication.

Ethical considerations

This research will be conducted in compliance with the protocol, Good Clinical Practice (GCP) guidelines, and all applicable regulatory requirements. A copy of the protocol, informed consent forms, and any other documents given to study participants will be submitted to the ethics committees (EC) of all the institutions involved. Written approval will be obtained for all subsequent amendments to the protocol, informed consent documents, and other study documentation referenced above. The investigator will notify the ECs and DSMC of violations of the protocol and serious adverse events. Ethical approval will be sought from the Ethics Committee of the Faculty of Medicine, Pharmacy, and Dentistry of the University of Science, Techniques, and Technologies of Bamako (Bamako, Mali) and the Research Ethics Committee of the London School of Hygiene & Tropical Medicine (London, UK). No activities will start before approval has been received by both committees.

Subject information and consent

Consenting procedures will vary based on age of the potential participant. Participants aged 18 years and above will provide informed consent. For participants under 18 years of age we will seek parental consent. In addition to parental consent, assent will be sought for children aged 10-17 years. The Ethics committee in Mali does not require that participants under the age of 12 provide assent.

The informed consent document will be used to explain the risks and benefits of study participation to the participant and in the case of participants < 18 years, the participant's parent in simple terms before the subject is enrolled in the study. The informed consent document contains a statement that the consent is freely given, that the subject is aware of the risks and benefits of entering the study, and that the subject is free to withdraw from the study at any time. Written consent must be given by the participant or the participant's parent, after the receipt of detailed information on the study. The informed consent form will be signed and personally dated by the participant or the participant's parent and the person who conducts the informed consent discussion. The original signed informed consent form will be retained in the participant's chart and another copy will be provided to him. A participant who is unable to read or write will place an imprint of his finger in place of a signature; in addition, an independent witness will sign the consent form to attest that the information in the consent form was orally conveyed to the participant. Specific consent will be provided for sharing samples and anonymized data to collaborating institutes outside Mali.

Confidentiality

The investigator will ensure that the subject's anonymity is maintained. Participants will not be identified in any publicly released reports of this study. All records will be kept confidential to the extent provided by laws and regulations. The study monitors and other authorized representatives of the regulatory authorities may inspect all documents and records required to be maintained by the Investigator.

All laboratory specimens, evaluation forms, reports, and other records that leave the site will be identified only by a coded number to maintain subject confidentiality.

Fully anonymized data may be uploaded to publicly available data repositories (e.g. https://datadryad.org/, https://www.biorxiv.org/) if requested as a pre-requisite for publication of this research. This will be explained in the participant information and during the consenting procedure. The upload of anonymized data will only be permitted if the participants/participants parents provide explicit approval during consent (a tick box in addition to the signature required for trial participation).

Risks

This study does not involve the use of antimalarial drugs at doses equal or lower than the recommended dosing and schedules. The dose of tafenoquine proposed in this study is far below the recommended dose for treatment and prevention of the malaria.

Tafenoquine can cause haemolytic anemia especially in individuals with G6PD deficiency and is not recommended during pregnancy because of the risk of the haemolytic anemia in a fetus who is G6PD deficient. A breastfed infant with G6PD deficiency is also at risk for hemolytic anemia from exposure to tafenoquine. For these reasons, pregnant or breastfeeding women will not be illegible for this study. The most common adverse reactions of tafenoquine include:

dizziness, nausea, vomiting, headache, and changes in laboratory tests for hemoglobin. This side effect of the treatment, which has no clinical consequences, will be explained in detail. Tafenoquine can also cause other side effects. Venipuncture are associated with pain and bruising at the site of the prick, and rarely infection.

Risks will be minimized by the study sites by ensuring adequate training of staff in all procedures and supply of consumables. The study site adheres to the standards of GCP and will be monitored.

Benefits

The benefits of the participation include free treatment of malaria and other acute diseases that may occur during the follow-up period. Participants who are in the groups that receive tafenoquine may be less infectious to mosquitoes and reduce the chance of having mosquitoes in their environment becoming infectious. Findings will help in the treatment of future patients and communities with malaria and further optimize the design of community intervention programmes.

Compensation

Participants will receive compensation for the time and travel for protocol specified visits. This compensation will be carefully evaluated and provided upon agreement of the local ethics committee. In Mali, the estimated cost of time and travel expense per visit in Ouelessebougou for residents of Ouelessebougou is estimated to be 1500 CFA (~3 US $). The compensation for enrolment visit will be 3000 CFA (~6.0 US $) and 1500 CFA (~3 US $) for each additional visit.

Use and storage of study samples

Samples collected will be stored at MRTC in Bamako, Mali and some will also be shipped to collaborating research centres such as the London School of Hygiene & Tropical Medicine and/or Radboud University medical center to perform specific tests as described above. Samples may be kept for a maximum of 10 years.

Sponsor information

Indemnity

London School of Hygiene & Tropical Medicine holds Public Liability ("negligent harm") and Clinical Trial ("non-negligent harm") insurance policies which apply to this trial.

Sponsor

London School of Hygiene & Tropical Medicine will act as the main sponsor for this study. Delegated responsibilities will be assigned locally.

Audits and Inspections

The study may be subject audit by the London School of Hygiene & Tropical Medicine under their remit as sponsor, the Study Coordination Centre and other regulatory bodies to ensure adherence to GCP.

References

1. World Health Organisation. World Malaria Report. Geneva, Switzerland; 2018.
2. Das S, Saha B, Hati AK, Roy S. Evidence of Artemisinin-Resistant Plasmodium falciparum Malaria in Eastern India. New England Journal of Medicine. 2018;379(20):1962-4.
3. Sawa P, Shekalaghe SA, Drakeley CJ, Sutherland CJ, Mweresa CK, Baidjoe AY, et al. Malaria transmission after artemether-lumefantrine and dihydroartemisinin-piperaquine: a randomized trial. J Infect Dis. 2013;207(11):1637-45.
4. Schneider P, Bousema T, Omar S, Gouagna L, Sawa P, Schallig H, et al. (Sub)microscopic Plasmodium falciparum gametocytaemia in Kenyan children after treatment with sulphadoxine-pyrimethamine monotherapy or in combination with artesunate. Int J Parasitol. 2006;36(4):403-8.
5. Bradley J, Soumaré HM, Mahamar A, Diawara H, Roh M, Delves M, et al. Transmission-blocking Effects of Primaquine and Methylene Blue Suggest Plasmodium falciparum Gametocyte Sterilization Rather Than Effects on Sex Ratio. Clin Infect Dis. 2019;69(8):1436-9.
6. Dicko A, Roh ME, Diawara H, Mahamar A, Soumare HM, Lanke K, et al. Efficacy and safety of primaquine and methylene blue for prevention of Plasmodium falciparum transmission in Mali: a phase 2, single-blind, randomised controlled trial. Lancet Infect Dis. 2018;18(6):627-39.
7. Dicko A, Brown JM, Diawara H, Baber I, Mahamar A, Soumare HM, et al. Primaquine to reduce transmission of Plasmodium falciparum malaria in Mali: a single-blind, dose-ranging, adaptive randomised phase 2 trial. Lancet Infect Dis. 2016.
8. Stone WJR, Mahamar, A., Bousema, T., Dicko, A., Drakeley, C., . Pyronaridine-artesunate or dihydroartemisinin-piperquine combined with low dose primaquine for preventing P. falciparum malaria transmission: A single-blind randomized clinical trial in Ouelessebougou, Mali. Lancet Microbe in revision. 2021.
9. Bousema JT, Schneider P, Gouagna LC, Drakeley CJ, Tostmann A, Houben R, et al. Moderate Effect of Artemisinin-Based Combination Therapy on Transmission of Plasmodium falciparum. J Infect Dis. 2006;193(8):1151-9.
10. Sutherland CJ, Ord R, Dunyo S, Jawara M, Drakeley CJ, Alexander N, et al. Reduction of Malaria Transmission to *Anopheles* Mosquitoes with a Six-Dose Regimen of Co-Artemether. PLoS Med. 2005;2(4):e92.
11. World Health Organisation. Global Malaria Programme: WHO policy brief on single-dose primaquine as gametocytocide in *Plasmodium falciparum* malaria. 2015.
12. Chen I, Diawara H, Mahamar A, Sanogo K, Keita S, Kone D, et al. Safety of Single-Dose Primaquine in G6PD-Deficient and G6PD-Normal Males in Mali Without Malaria: An Open-Label, Phase 1, Dose-Adjustment Trial. The Journal of Infectious Diseases. 2018:jiy014-jiy.
13. Bastiaens GJH, Tiono AB, Okebe J, Pett HE, Coulibaly SA, Gonçalves BP, et al. Safety of single low-dose primaquine in glucose-6-phosphate dehydrogenase deficient falciparum-infected African males: Two open-label, randomized, safety trials. PLOS ONE. 2018;13(1):e0190272.
14. Raman J, Allen E, Workman L, Mabuza A, Swanepoel H, Malatje G, et al. Safety and tolerability of single low-dose primaquine in a low-intensity transmission area in South Africa: an open-label, randomized controlled trial. Malar J. 2019;18(1):209.
15. Eziefula AC, Bousema T, Yeung S, Kamya M, Owaraganise A, Gabagaya G, et al. Single dose primaquine for clearance of Plasmodium falciparum gametocytes in children with uncomplicated malaria in Uganda: a randomised, controlled, double-blind, dose-ranging trial. Lancet Infect Dis. 2014;14(2):130-9.
16. Hounkpatin AB, Kreidenweiss A, Held J. Clinical utility of tafenoquine in the prevention of relapse of Plasmodium vivax malaria: a review on the mode of action and emerging trial data. Infect Drug Resist. 2019;12:553-70.
17. Crockett M, Kain KC. Tafenoquine: a promising new antimalarial agent. Expert Opinion on Investigational Drugs. 2007;16(5):705-15.

18. Lacerda MVG, Llanos-Cuentas A, Krudsood S, Lon C, Saunders DL, Mohammed R, et al. Single-Dose Tafenoquine to Prevent Relapse of Plasmodium vivax Malaria. New England Journal of Medicine. 2019;380(3):215-28.
19. Llanos-Cuentas A, Lacerda MV, Rueangweerayut R, Krudsood S, Gupta SK, Kochar SK, et al. Tafenoquine plus chloroquine for the treatment and relapse prevention of Plasmodium vivax malaria (DETECTIVE): a multicentre, double-blind, randomised, phase 2b dose-selection study. The Lancet. 2014;383(9922):1049-58.
20. Llanos-Cuentas A, Lacerda MVG, Hien TT, Vélez ID, Namaik-larp C, Chu CS, et al. Tafenoquine versus Primaquine to Prevent Relapse of Plasmodium vivax Malaria. New England Journal of Medicine. 2019;380(3):229-41.
21. Baird JK SI, Soebandrio A. Evaluation of the efficacy and safety of tafenoquine co-administered with dihydroartemisinin-piperaquine for the radical cure (anti-relapse) of Plasmodium vivax malaria in Indonesia - INSPECTOR study. Am Soc Trop Med Hyg Virtual2020.
22. CDC GH, Division of Parasitic Diseases and Malaria. Change in Krintafel (tafenoquine) Label 2020 [updated 24/02/2020. Available from: https://www.cdc.gov/malaria/new_info/2020/tafenoquine_2020.html.
23. World Health Organisation. WHO policy recommendation: Seasonal malaria chemoprevention (SMC) for Plasmodium falciparum malaria control in highly seasonal transmission areas of the Sahel sub-region in Africa. Geneva, Switzerland; 2012.
24. Cappellini MD, Fiorelli G. Glucose-6-phosphate dehydrogenase deficiency. Lancet. 2008;371(9606):64-74.
25. White NJ, Qiao LG, Qi G, Luzzatto L. Rationale for recommending a lower dose of primaquine as a Plasmodium falciparum gametocytocide in populations where G6PD deficiency is common. Malar J. 2012;11:418.
26. World Health Organisation. Updated WHO policy recommendation October 2012: single dose primaquine as a gametocytocide in Plasmodium falciparum malaria. Geneva: World Health Organization; 2012.
27. Chen IT, Gosling RD. Targeting Plasmodium falciparum transmission with primaquine: same efficacy, improved safety with a lower dose? Expert Rev Clin Pharmacol. 2014;7(6):681-6.
28. Eurartesim dosing guidelines [Available from: http://www.ema.europa.eu/docs/en_GB/document_library/EPAR_-_Product_Information/human/001199/WC500118113.pdf.
29. Rueangweerayut R, Bancone G, Harrell EJ, Beelen AP, Kongpatanakul S, Möhrle JJ, et al. Hemolytic Potential of Tafenoquine in Female Volunteers Heterozygous for Glucose-6-Phosphate Dehydrogenase (G6PD) Deficiency (G6PD Mahidol Variant) versus G6PD-Normal Volunteers. Am J Trop Med Hyg. 2017;97(3):702-11.
30. Hellweg S, Canals LM. Emerging approaches, challenges and opportunities in life cycle assessment. Science. 2014;344:1109-13.
31. Burtscher L, Barrett D, Borkar AP, Grinberg V, KJahnke K, Kendrew S, et al. The carbon footprint of large astronomy meetings. Nature Astronomy. 2020;4:823-5.
32. Bousema T, Selvaraj P, Djimde AA, Yakar D, Hagedorn B, Pratt A, et al. Reducing the Carbon Footprint of Academic Conferences: The Example of the American Society of Tropical Medicine and Hygiene. Am J Trop Med Hyg. 2020;103(5):1758-61.
33. Barret D. Estimating, monitoring and minimizing the travel footprint associated with the development of the Athena X-ray Integral Field Unit. Experimental Astronomy 2020;49:183–216.
34. https://www.sustainabilityexchange.ac.uk/leaf_a_new_approach_to_achieving_laboratory_sus [
35. Bousema T, Okell L, Shekalaghe S, Griffin J, Omar S, Sawa P, et al. Revisiting the circulation time of *Plasmodium falciparum* gametocytes: molecular detection methods to estimate the duration of gametocyte carriage and the effect of gametocytocidal drugs. Malar J. 2010;9(1):136.

36. Méndez F, Muñoz A, Plowe CV. Use of area under the curve to characterize transmission potential after antimalarial treatment. Am J Trop Med Hyg. 2006;75(4):640-4.

Appendix

SUMMARY OF TAFENOQUINE SAFETY DATA

for

NECTAR 3: A four-arm trial comparing artemether-lumefantrine with or without single-dose primaquine and sulphadoxine-pyrimethamine/amodiaquine with or without single-dose tafenoquine to reduce P. falciparum transmission in Mali

Date:	14 May 2021
Edition No.	1.0
Authors:	Merel Smit (RUMC)

Safety in humans

Introduction

After being under development since 1980, tafenoquine (TQ) has received approval by the Food and Drug Administration (FDA) in 2018 as an anti-relapse drug for *P. vivax* malaria in patients aged 16 years and older and for prophylaxis of malaria caused by any *Plasmodium* species in adults.

TQ belongs to the same drug family (8-aminoquinolines) as primaquine (PQ), but has a longer duration of activity and far greater stability in the blood compared to PQ. Its ability to block *P. falciparum* transmission after artemisinin combination therapy (ACT) treatment has only recently been assessed and indicates potent but delayed activity. There are indications from unpublished trials that TQ may be better administered with non-ACTs or on its own, due to metabolic inhibition by artemisinin derivatives.

Combination of TQ with non-ACTs for a *P. falciparum* gametocytocidal indication has not been tested. Sulfadoxine-pyrimethamine (SP) with amodiaquine (AQ) is a non artemisinin based combined anti-malarial treatment (SPAQ) that is highly effective against *P. falciparum*. Currently SPAQ is used in seasonal malaria chemoprevention (SMC) throughout the Sahel region. SPAQ with single low-dose of primaquine (SLD PQ) has been tested, but SPAQ with TQ has not [Dicko et al. 2018]. In the NECTAR 3 trial, the gametocytocidal and transmission blocking efficacy of single weight based dose of TQ (1.66mg/kg) in combination with SPAQ will be tested in G6PD normal Malian children and adults (10-50 years).

Tafenoquine dosing will be weight based (1.66mg/kg) in order to standardise efficacy and risk variance. The TQ dose of 1.66mg/kg is equivalent to a 100 mg single dose in a 60kg adult. The maximum weight of included individuals is 80kg. Tafenoquine will be dosed in 5kg bands, the maximum TQ dose given in this trial will therefore be 128.66 mg.

Safety data from across the whole TQ development program includes mostly studies wherein participants were exposed to (much) higher TQ doses than the doses that will be used in the NECTAR 3 trial. Of the 4129 subjects exposed to TQ, less than 10% (n=392) received a dose <300 mg [Krintafel FDA-2018].

Much of the safety data from these studies was summarized in two briefing documents submitted to the U.S. FDA in the process of obtaining regulatory approval for TQ-containing products [Krintafel FDA-2018, Arakoda ADAC-2018], as well as in a recent review [Hounkpatin-2019].

This document gives an overview of the known adverse events (AEs), serious adverse events (SAEs), groups at risk, precautions and our recent findings from the NECTAR 2 trial (study arms: DP (n=20), DP+TQ 0.415mg/kg (n=20), DP+TQ 0.83mg/kg (n=20), DP+TQ 1.66mg/kg (n=20)) in the proposed study population.

1. Summary of Safety Data

The most common AEs in participants who received a single dose ≤300mg tafenoquine are: headache, dizziness, chills, asthenia, nausea, vomiting, diarrhea, abdominal pain, increased alanine aminotransferase (ALT), hemoglobin (Hb) decrease, insomnia, and arthralgia. These adverse events were generally mild to moderate in severity. Overall numbers of AEs were generally no greater at these doses than with placebo. The only reported SAEs related to TQ dose ≤300mg are hematological SAEs.

Of the AEs considered to be of special interest in TQ trials due to its similarity to other drugs with well-known AE profiles, there appears to be no elevated risk of clinically significant methaemoglobinemia, cardiac, neurological/psychiatric or ophthalmologic AEs in the general population at the doses that will be administered in the NECTAR 3 trial. The risk of anemia, particularly in Glucose-6-Phosphate Dehydrogenase (G6PD) deficient subjects, though still expected to be low at these doses, remains of theoretical concern. TQ has not been associated with other clinically significant (specifically hepatobiliary or renal) laboratory abnormalities at these doses.

Precautions

- *G6PD deficiency:* G6PD testing must be performed before administration of tafenoquine and G6PD-deficient subjects should be excluded due to the risk of hemolytic anemia.
- *Pregnancy or Lactation:* tafenoquine may cause fetal harm when administered to a pregnant woman with a G6PD-deficient fetus. Pregnant women should therefore be excluded and all sexually-active women of child-bearing potential should use effective contraception throughout the study until 3 months after tafenoquine intake. A G6PD-deficient infant may also be at risk for hemolytic anemia from exposure to tafenoquine through breast milk. Before breastfeeding, check infant's G6PD status. No breastfeeding is advised for 3 months after the last dose of tafenoquine.
- *History of psychiatric disorders:* serious psychiatric adverse events have been observed in patients with a psychiatric history and high cumulative doses of tafenoquine. Subjects with such a history should therefore be excluded.
- *Hypersensitivity reactions:* patients with known hypersensitivity reactions to (any component of) tafenoquine or other 8-aminoquinolines should be excluded.
- *Methaemoglobinemia:* since asymptomatic elevations in blood methaemoglobin have been observed following tafenoquine intake, this should be monitored.
- *Delayed Adverse Reactions:* due to the long half-life ($t_{1/2}$) of tafenoquine (average $t_{1/2}$ of 16.5 days), hemolytic anemia, methaemoglobinemia, hypersensitivity reactions, and psychiatric effects may be delayed in onset and/or duration.
- *Drugs with a narrow therapeutic index that are substrates of the renal transporters OCT2 and MATE:* tafenoquine is an inhibitor of human transporters organic cation transport 2 (OCT2) and multidrug and toxin extrusion transporter (MATE) in vitro, and this may result in increased exposure to their substrates. Co-administration with drugs that are substrates of organic cation transporter-2 (OCT2) or multidrug and toxin extrusion (MATE) transporters should be avoided.
- *Metformin:* tafenoquine should not be used in subjects with elevated creatinine levels concomitantly taking metformin, due to the small risk of lactic acidosis.

2. Adverse Events

General

The most common AEs in participants who received a single dose ≤300mg tafenoquine are: headache, dizziness, chills, asthenia, nausea, vomiting, diarrhea, abdominal pain, increased alanine aminotransferase (ALT), hemoglobin decrease, insomnia, and arthralgia [Llanos-

Cuentas-2014; Lacerda-2019; Llanos-Cuentas-2019]. These AEs were generally mild to moderate in severity.

In a randomized, double-blind, placebo-controlled chemoprophylaxis trial participants received a loading dose of TQ (25mg (n=93), 50mg (n=93), 100mg (n=94) or 200mg (n=93)) for three days, followed by a weekly dose for 12 weeks [Hale-2003]. The TQ groups all demonstrated a comparable overall number of AEs in comparison to the placebo group (n=94).

Given the known safety profile of TQ-related drugs, clinical trials of TQ have focused on several AEs of special interest. These include: hematologic, cardiac, neurologic, psychiatric and ophthalmic side effects, as these are known to be prevalent at various degrees for related drugs. These potential safety issues are further discussed below.

In our recent NECTAR 2 trial, the most common possibly related adverse events that occurred were: nausea, fever, headache, emesis, dizziness and abdominal pain. The overall numbers of adverse events were similar between the study arms.

Serious Adverse Events

No SAEs related to TQ dosage ≤300mg are reported except for hematological SAEs [Hale-2003; Llanos-Cuentas-2014; Lacerda-2019; Llanos-Cuentas-2019]. See 'Hematologic Safety' and 'G6PD-deficiency', as discussed below, for these hematological serious adverse events related to TQ administration.

In the NECTAR 2 trial, no (related) SAEs following administration of up to 1.66mg/kg (equivalent to 100mg in adults) as a single dose in Malian males and non-pregnant females over 12 years of age (Mahamar, Smit et al. 2021 *In preparation*).

Adverse Events of Special Interest

Hematologic Safety

As an analogue of PQ, TQ might be expected to share the same profile for hematological AEs as PQ. These hematological AEs include: anemia, methaemoglobinemia, leukopenia, and hemolytic anemia in individuals with G6PD deficiency [Sanofi-Aventis-2016].

In a dose-ranging phase 2b study three hematological SAEs occurred in subjects receiving a single dose of TQ plus 3-day chloroquine (CQ) [Llanos-Cuentas-2014]. Subjects in the study received a single dose of either 50mg (n=55), 100mg (n=57), 300mg (n=57) or 600mg (n=56) tafenoquine. Two of the SAEs were defined as anemia and occurred in subjects in the 100 and 300mg TQ dose group. The other SAE was defined as hemoglobin decline and occurred in a subject in the 600mg TQ dose group. These hematological events did not fulfill the criteria for 'serious', such as life-threatening or requiring hospitalization. In all three subjects, treatment was continued and hemoglobin recovered to normal concentrations with supportive care, no blood transfusions were needed. Investigation of these cases showed no evidence of drug-induced hemolysis. The reduction in hemoglobin occurred shortly after initiation of antimalarial therapy and was attributed to rehydration. Genotyping showed that none of these subjects had G6PD variants associated with enzyme deficiency.

In a phase 3 study a hemoglobin decline of >30g/L or ≥30% of the baseline level was shown in 5.4% (14 of 260 patients) of the group receiving TQ single doses of 300mg [Lacerda-2019]. These adverse events were 'treatment stopping criteria' but were not considered serious adverse events. The nadir was a hemoglobin level of 8.8g/dl. None of these patients experienced symptoms of anemia, and the hemoglobin levels subsequently returned to the normal range without clinical intervention. In this trial there were no serious adverse (hematological) events attributed to TQ.

In these studies the single doses of TQ given is higher (300 – 600mg) or slightly less (100mg) than the maximum dose (128.66mg) that will be administered in the NECTAR 3 trial.

We observed no clinically significant haemoglobin decline following administration of 1.66mg/kg TQ (equivalent to 100mg in adults) as a single dose in Malian males and non-pregnant females over 12 years of age in the NECTAR 2 trial (Mahamar, Smit et al. 2021 *In preparation*). An overview of the hemoglobin levels in the different study arms up to day 28 after treatment is presented below in figure 1.

To ensure hematological safety and prevent clinically significant drug-induced anemia or hemolysis, G6PD deficiency and hemoglobin <10g/dl are part of the exclusion criteria of the NECTAR 3 trial.

Methaemoglobinemia

As with PQ, an increase in methaemoglobin levels may occur with TQ administration. The physiological concentrations of methaemoglobin is normally maintained at approximately 1-2% by enzymes inside RBCs. Methaemoglobin levels of 1-3% are usually asymptomatic. Higher levels of 3-15% methaemoglobin may also still be asymptomatic. However, cyanosis may occur at levels above 15% and at levels of 20-50% patients often show headache, fatigue, weakness, dizziness, dyspnea and syncope [Hunter-2011].

Among the subjects (n=77) in a phase 3 trial who received a loading dose regimen of 200mg TQ daily for three consecutive days followed by a weekly maintenance dose of 200mg TQ over approximately 6 months, mean methaemoglobin levels increased by 1.8%. By week 12 of follow-up, the increase in methaemoglobin had resolved [Nasveld-2010].

In another randomized clinical trial, subjects were assigned to 1 of 4 drug regimens during 12 weeks: placebo throughout; 3 days of 400mg of TQ per day, followed by placebo weekly (n=60); 3 days of 200mg of TQ per day, followed by 200mg per week (n=55); and 3 days of 400mg of TQ per day, followed by 400mg per week (n=59) [Shanks-2001]. Among the subjects who received 200mg weekly dose TQ, mean plateau concentrations of methaemoglobin (±SD) were 2.5% ± 1.6%. For the subjects who received 400mg per week, mean plateau concentrations of methaemoglobin (±SD) were 4.5% ± 2.5%.

In a phase 3 trial, maximum methaemoglobin levels were 13.0% in the TQ group (n=260) who received a single dose of 300mg TQ [Lacerda-2019]. In 1.2% of the volunteers in the TQ group the methaemoglobin levels were at least 10%. However, no clinical signs of methaemoglobinemia were observed.

This indicates that the methaemoglobin levels may exceed the physiological norm when administering ≥ 300mg TQ.

The doses used in the other trials (300mg single dose or cumulative doses of 1200mg – 5600mg) are at least 2 times the highest dose that will be used in the NECTAR 3 trial (128.66mg). We therefore assess the risk of clinically significant elevations of methaemoglobin to be low to negligible in the NECTAR 3 trial. To ensure a safely conducted trial, methaemoglobin measurements will be performed in the follow-up visits of the trial, as has been performed in the NECTAR 2 trial. An overview of these methaemoglobin levels during follow-up are presented in the figure below.

Figure 1. The haemoglobin and methaemoglobin levels up to day 28 after start treatment for each of the four arms in the NECTAR 2 trial. Overall, there were no significant differences in hemoglobin decline between study arms. Methaemoglobin levels stayed below 3.5% and none of the patients were symptomatic. *Of note, one individual in the 1.66mg/kg TQ arm was lost to follow up after their visit at day 7. At day 7 this individual reported no AE's and recorded an Hb density of 10.1g/dL.

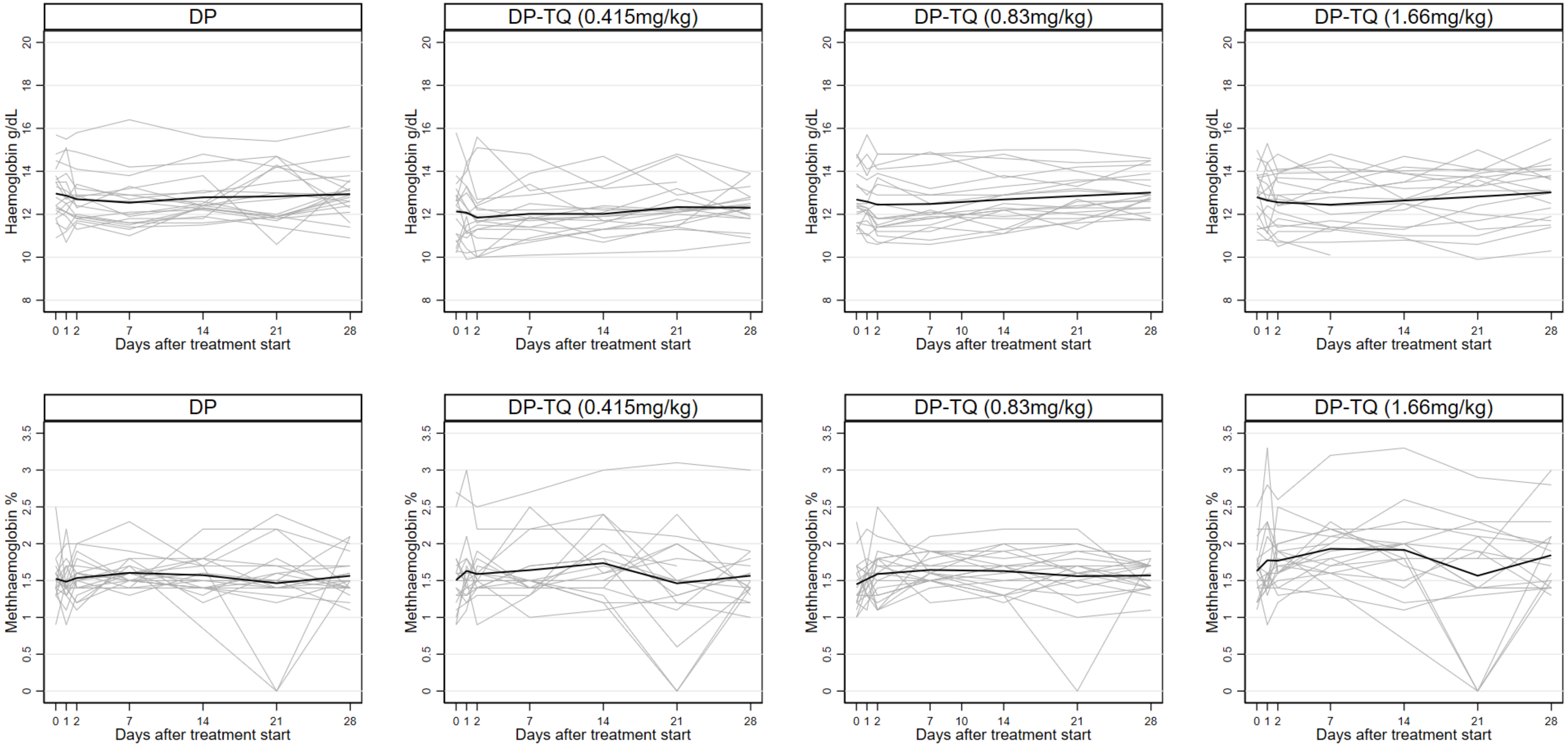

G6PD deficiency

The longer half-life of TQ also has its disadvantage: G6PD-deficient individuals are at a potential greater risk of hemolysis, as active concentrations of TQ will remain in the body for several weeks.

TQ, as an 8-aminoquinoline, can cause hemolysis in individuals with a deficiency in G6PD enzyme activity, a hereditary X-linked condition [Cappellini-2008]. The key factors determining the severity of drug-induced hemolysis are dose and the degree of G6PD enzyme activity.

In a phase 1 open-label, dose-escalation study of the hemolytic potential of TQ, an evident dose response for hemolysis was noted for G6PD-deficient females [Rueangweerayut-2017]. Females with G6PD deficiency enzyme activity of 40-60% received a single dose of 100mg (n=6), 200mg (n=6) of 300mg (n=3) TQ. Compared with G6PD-normal subjects, the maximum decrease in hemoglobin in G6PD-heterozygous subjects was slightly greater for TQ 100mg, but this difference became more pronounced at TQ 200mg and was evidently greater with the 300mg dose, with dose-limiting toxicity (hemoglobin decline of ≥ 2,5g/dL and/or hematocrit decrease of ≥7,5% from pretreatment) occurring in 3/3 subjects.

Although this is a phase 2 study with small sample size, based on this data, it might be possible to administer a low single dose of TQ administration (≤100mg) in G6PD deficient people with an enzyme activity of 40-60%. However, more studies will have to follow to ensure the safety of administering a low single dose of TQ in G6PD-deficient people.

Although all the other tafenoquine studies have excluded subjects with G6PD deficiency, in some clinical trials subjects with G6PD deficiency were inadvertently recruited and received TQ regimens. In most cases, this inadvertent recruitment was due to the inherent limitations of G6PD phenotyping tests or to human error. 8 cases described inadvertently recruited G6PD deficient subjects, all receiving TQ doses ≥300mg, and Hb decreases of 1,9-5,4g/dL [Shanks-2001, Miller-2013, Green-2014, Llanos-Cuentas-2014, NCT02488980]. Most subjects did not show any clinical symptoms, 2 out of the 8 cases were symptomatic and required treatment [Shanks-2001, NCT02488980]. These subjects had received cumulative TQ doses of 600 and 1200mg.

The regional prevalence of G6PD deficiency in Africa ranges from 15 to 26% [Howes-2012]. In Kambila (25 km from Bamako, Mali) a prevalence of G6PD deficiency as high as 17% has been documented [Crompton-2008]. The frequency of G6PD deficiency is 10-15% in the region where the NECTAR 3 trial will be conducted.

TQ is co-administered with SPAQ in this trial. Sulphadoxine and pyrimethamine target enzymes involved in folate synthesis. Sulphadoxine can precipitate hemolysis in patients with glucose-6-phosphate dehydrogenase (G6PD) deficiency.

Since the safety of TQ doses ≥100mg in G6PD-deficient persons remains of concern and the safety of doses <100mg has been insufficiently studied, all volunteers willing to participate in the NECTAR 3 trial will be screened for G6PD deficiency. Subjects with G6PD deficiency and/or hemoglobin < 10 g/dL will be excluded from the trial.

Cardiac Safety

As an analogue of PQ, there is the possibility that TQ could share PQ's risk of cardiac side effects, including cardiac arrhythmia and prolongation of the QT interval on ECG [Sanofi-Aventis, 2016]. In addition, amodiaquine is known to have a moderate risk of prolonging the QTc interval and bradycardia [Adjei-2021; Ngouesse-2001]. The risk or severity of corrected QT interval (QTc) can be increased when amodiaquine is combined with primaquine, an

analogue of TQ. However, in a cardiac safety study comparing doses of TQ (300mg and 600mg) with placebo, there was no indication of a QT prolongation effect [Green-2014]. The maximum effect on QTcF at the supratherapeutic dose of 1200mg compared to placebo was <10msec and within the FDA E14 guidelines for lack of effect. This study concluded that TQ did not have a clinically meaningful effect on cardiac repolarization. Furthermore, there was no dose-related increase in mean QTc change from baseline for TQ single dosages of 50mg to 600mg [Brueckner-1998, Llanos-Cuentas-2014].

Notably, most of these TQ doses are higher than the TQ doses that will be used in the NECTAR 3 trial. TQ shows no clinically relevant additional cardiac side effect. No extra measures have to be taken in this trial beyond the normal precautions for amodiaquine administration.

Neurologic/Psychiatric Events

Neurologic and psychiatric events are known side effects for some anti-malarials, including CQ, quinine, and mefloquine. These AEs are typically reversible and rarely persist long term.

The most common neurologic/psychiatric AEs that occurred in five phase 1-3 trials were: insomnia, anxiety, depression, and mainly headache and dizziness [Brueckner-1998, Lell-2000, Hale-2003, Nasveld-2010, Llanos-Cuentas-2014]. The TQ dose in these studies varied from 4 to 600mg single dose or a weekly dose of 200mg during 6 months. All the neurologic and psychiatric AEs were mild-to-moderate, transient and reversible.

A limited number of moderate and serious psychiatric adverse reactions, such as depression, suicidal behavior, and psychosis, have been reported. These events were mainly in relation with multiples doses and in individuals with a prior history of psychiatric disorders [Nasveld-2010, Shanks-2001]. The TQ dose regimen consisted of 200mg TQ base daily for three consecutive days, followed by an oral weekly maintenance dose of 200mg TQ over approximately 6 months.

Many participants of these trials were military personal that were in a “psychologically hostile environment” [Waller-2012]. In studies of prophylactic antimalarial drugs in military populations, there is evidence that the incidence of neuropsychiatric AEs (eg, adjustment disorder, insomnia, anxiety disorder) is higher in deployed versus non-deployed populations, especially when deployment occurs under combat conditions [Eick-Cost-2017, Kitchener-2005].

A review of psychiatric data from phase 3 studies revealed that the military subjects in those studies had a unique psychiatric AE profile compared to subjects in other TQ studies with the same dose regimen due to the combat environment to which these soldiers were exposed [Charles-2007, Nasveld-2002a, Nasveld-2010]. Despite the stressful environment to which these subjects were exposed, the incidence of psychiatric AEs was only 5.1%, with the majority of psychiatric AEs assessed as mild (84.4%) and considered not related or unlikely related to the study drug (52.0%).

The cumulative doses given in these trials area much higher than the single doses given in the NECTAR 3 trial. The study population of the NECTAR 3 trial consists of Malian civilians and not military population with concomitant stress factors.

There is no additional risk of central nervous system (CNS) effects to be expected during this trial in subjects without a history of serious psychiatric disorders.

Ophthalmic Safety

In common with other cationic amphiphilic drugs, TQ has the potential to cause phospholipid accumulation in the cornea, which manifests as vortex keratopathy. Vortex keratopathy was

reported in TQ studies which used >200mg TQ for longer durations [Halliwell-1997, Hollander-2004].

To provide further evidence of the ophthalmic safety of TQ at the dose used for antimalarial prophylaxis, a study was conducted in healthy volunteers to compare TQ 200mg weekly for 6 months versus placebo [Leary-2009]. In this study there was no evidence that exposure to TQ had any adverse effect on the retina. This study confirmed that administration of the TQ 200mg weekly for 6 months did not cause retinal toxicity in healthy subjects.

A specific phase 1 placebo-controlled ophthalmic safety study was conducted in healthy US volunteers using highly sensitive ophthalmic techniques to establish whether there is a risk of retinopathy with 300mg single dose of TQ. This study did not identify any signal for retinal toxicity with use of a single 300mg dose of TQ [Ackert, 2019].

Since the single dose TQ given in the NECTAR 3 trial will be lower than the (cumulative) TQ doses of the studies described above, the risks for retinal toxicity are considered minimal.

3. Laboratory Abnormalities

Hematological

See 'Adverse events - Hematologic safety' for hematological laboratory abnormalities and 'Adverse events - Methaemoglobinemia' for methaemoglobin laboratory abnormalities.

Hepatobiliary

Pooled results of four phase 2/3 clinical trials and one phase 1 clinical trial (n=825) showed no clinically significant hepatobiliary effects [Arakoda ADACM-2018]. These subjects received a loading dose of 200mg TQ per day for 3 days followed by a maintenance dose of 200mg weekly.

Transient, asymptomatic dose-related elevation in liver transaminases (ALT and AST) were observed in healthy volunteers who received TQ single doses of 300mg, 600mg and 1200mg in placebo-controlled phase 1 studies [Krintafel FDA-2018]. At the 300mg dose, elevations were mild-to-moderate. None of the elevations were severe or considered to be clinically significant.

Since the doses of TQ used in these trials are more than 2 times the maximum dose used in the NECTAR 3 trial, the estimated risk of clinically significant hepatobiliary effects is considered minimal.

In a randomized, double-blind, placebo-controlled chemoprophylaxis trial in Ghana, abnormally elevated ALT levels (61.9-193U/L) were observed in all study arms [Hale-2003]. Participants received either a loading dose of TQ for 3 days followed by a weekly dose of TQ (25mg, 50mg, 100mg or 200mg), 250mg mefloquine per week (positive reference) or placebo (negative reference). The abnormal ALT levels were mostly detected in week 4 during routine blood test. Nearly every subject, irrespective of study arm, experienced a notable, predominantly mild elevation in ALT level from baseline to week 4. There were no dose-related differences between TQ groups in either the frequency or magnitude of elevated ALT values. Abnormally elevated ALT levels improved or normalized after cessation of TQ therapy.

After 4 weeks, the cumulative doses of the TQ groups were 150mg, 300mg, 600mg and 1200mg TQ. These doses are more than the maximum dose given in the NECTAR 3 trial. Based on these data, no clinically significant abnormal hepatobiliary levels are to be expected in the NECTAR 3 trial.

No clinically significant abnormal ALT or AST levels were observed during follow-up of the NECTAR 2 trial.

Renal

Safety findings from nonclinical studies of TQ suggest that the drug might have renal effects (tubular nephropathy, necrosis, and dilatation). Pooled results of four phase 2/3 clinical trials and one phase 1 clinical trial (n=825) showed no clinically significant renal effects [Arakoda ADACM-2018]. The study objective of the phase 1 study was renal safety, and showed no mean change from baseline Glomerular Filtration Rate (GFR) at 24 weeks for TQ versus placebo [Leary-2009].

Single dose of 300mg TQ was associated with small reversible increases in creatinine, which were consistent with the known renal transporter inhibition effect (OCT2 and MATEs).

During the NECTAR 2 trial, no abnormal clinically significant creatinine levels were observed.

Based on this data, no clinically significant renal adverse effects are to be expected in the NECTAR 3 trial.

4. Drug interactions

Anti-malarial drugs

In the NECTAR 3 trial TQ will be co-administered with SPAQ. TQ can be administered without dose adjustment with other commonly prescribed anti-malarial drugs such as CQ, DHA/PPQ or AL [Green-2016]. However, recent studies indicate that ACT may inhibit TQ activity. To date, no drug interactions have been reported between TQ and SPAQ.Organic Cation Transporter-2 (OCT2) and Multidrug and Toxin Extrusion (MATE) Substrates

TQ is an inhibitor of human transporters organic cation transporter 2 (OCT2) and multidrug and toxin extrusion transporter (MATE) in vitro and this may result in increased exposure to their substrates. There is a small risk of lactic acidosis due to increased metformin exposure secondary to the blockade of these transporters. Patients with creatinine above the normal range should therefore not be co-dosed with metformin, because of the increased risk of lactic acidosis. Drugs with a narrow therapeutic index that are substrates of the renal transporters OCT2 and MATE (e.g. phenformin, buformin, dofetilide, procainamide, and pilsicainide) should not be co-administered with TQ.

Individuals using metformin or drugs with a narrow therapeutic index that are substrates of the renal transporters OCT2 and MATE are excluded from the NECTAR 3 trial.

5. Groups at risk

G6PD deficiency

See ‘Adverse events – G6PD deficiency’.

Pregnancy and lactation

Pregnant women have been routinely excluded in clinical trials of TQ, hence there is limited data on the safety of TQ in pregnancy. Studies in animals have shown no adverse effects of TQ on embryofetal development at concentrations higher compared to those that will be achieved in the NECTAR 3 trial. However, TQ must not be used in pregnancy because of a risk of hemolysis in G6PD deficiency and the danger of acute hemolytic anemia in the fetus. Even if

a pregnant woman is not G6PD deficient, the fetus may be G6PD deficient. Therefore TQ should not be given to pregnant or lactating women [Krintafel FDA-2018]. The screening for the NECTAR 3 trial includes a pregnancy test for females. Pregnant women are excluded from the trial.

Consistent with the long half-life of TQ, effective contraception should be continued for 5 half-lives (3 months) after the end of treatment [Arakoda ADACM-2018].

Whether TQ is excreted in human milk is not known. TQ should therefore not be used during breastfeeding as drug-induced hemolytic anemia may occur when the infant has G6PD deficiency or the G6PD status is unknown.

Children

Safety and effectiveness of TQ in children <16 years have not been published. Studies regarding the safety of TQ administration were conducted in adults. A phase 2 study in Gabon studied various TQ loading doses for prophylaxis in semi-immune African children and young adults aged 12–20 years [Lell, 2000]. Participants received 250mg halofantrine daily for 3 days as initial curative treatment and a week later were assigned to 25mg (n=79), 50mg (n=86), 100mg (n=79), 200mg (n=84) TQ or placebo daily for 3 days. The number of adverse events did not differ significantly between the treatment groups. TQ was well tolerated in subjects aged 12-20 years. The AEs were generally mild and self-limiting and none were considered serious. No AEs were associated with TQ. Hemoglobin concentrations were slightly (0.4 g/dL) but significantly lower than at screening in the group receiving 200mg TQ.

A phase 2 multicenter study assessed the pharmacokinetics, safety and efficacy of tafenoquine in pediatric subjects with *P. vivax* aged 6 months to <16 years of age. All subjects received treatment with chloroquine (CQ) and were assigned to a single, weight based, dose of 100mg (n=14), 150mg (n=5), 200mg (n=22) or 300mg (n=19) TQ. The most common adverse event was emesis. A hemoglobin decline of >20-≤30g/L was shown in one patient who had received the 200mg TQ dose. All the other participants had a haemoglobin decline of less than 20g/L. In this trial no serious adverse (hematological) events were attributed to TQ [NCT02563496, not published]. These findings provide some reassurance concerning TQ administration in children.

In the abovementioned trials, TQ was safely administered in children. The TQ doses given in the trial in Gabon are in the same range as those given in the NECTAR 3 trial, except for the duration. The majority of the doses given in the NECTAR 3 trial are less than the (cumulative) doses in the trials above.

In the NECTAR 3 trial individuals between 10 and 50 years are included.

6. References

- Ackert J, Mohamed K, Slakter JS, et al. Randomized Placebo-Controlled Trial Evaluating the Ophthalmic Safety of Single-Dose Tafenoquine in Healthy Volunteers. *Drug Saf*. 2019;42(9):1103–1114. doi:10.1007/s40264-019-00839-w
- Adjei GO, Oduro-Boatey C, Rodrigues OP, Hoegberg LC, Alifrangis M, Kurtzhals JA, et al. Electrocardiographic study in Ghanaian children with uncomplicated malaria, treated with artesunate-amodiaquine or artemether-lumefantrine. Malar J. 2012;11:420.
- Arakoda (Tafenoquine succinate) tablets for the prevention of malaria in adults; NDA 210607; Briefing Document for the Antimicrobial Drugs Advisory Committee; July 26, 2018; https://www.fda.gov/media/114755/download

- Brueckner RP1, Lasseter KC, Lin ET, Schuster BG. First-time-in-humans safety and pharmacokinetics of WR 238605, a new antimalarial. Am J Trop Med Hyg. 1998a;58:645 9.
- Cappellini MD, Fiorelli G. Glucose-6-phosphate dehydrogenase deficiency. Lancet. 2008;371(9606):64-74. doi: 10.1016/S0140-6736(08)60073-2.
- Charles BG, Miller AK, Nasveld PE, Reid MG, Harris IE, Edstein MD. Population pharmacokinetics of tafenoquine during malaria prophylaxis in healthy subjects. Antimicrob Agents Chemo. 2007;51:2709-17.
- Crompton PD, Traore B, Kayentao K, Doumbo S, Ongoiba A, Diakite SA, Krause MA, Doumtabe D, Kone Y, Weiss G, Huang CY, Doumbia S, Guindo A, Fairhurst RM, Miller LH, Pierce SK, Doumbo OK. Sickle cell trait is associated with a delayed onset of malaria: implications for time-to-event analysis in clinical studies of malaria. J Infect Dis. 2008;198:1265–1275.
- DF Clyde, et al. Clinical problems associated with the use of primaquine as a tissue schizontocidal and gametocytocidal drug. Bull World Health Organ, 59 (1981), pp. 391-395.
- Dicko, A. ; Roh, M.E. ; Diawara, H. ; Mahamar, A. ; Soumare, H.M. ; Lanke, K. ; Bradley, J. ; Sanogo, K. ; Kone, D.T. ; Diarra, K. ; Keita, S. ; Issiaka, D. ; Traore, S.F. ; McCulloch, C. ; Stone, W. ; Hwang, J. ; Müller, O. ; Brown, J.M. ; Srinivasan, V. ; Drakeley, C. ; Gosling, R. ; Chen, I. ; Bousema, T. (2018) Efficacy and safety of primaquine and methylene blue for prevention of Plasmodium falciparum transmission in Mali: a phase 2, single-blind, randomised controlled trial. Lancet Infect Dis; DOI: 10.1016/S1473-3099(18)30044-6; PMID: 29422384
- Eick-Cost AA, Hu Z, Rohrbeck P, Clark LL. Neuropsychiatric outcomes after mefloquine exposure among US military service members. Am J Trop Med Hyg. 2017;96:159-66.
- Green JA, Mohamed K, Goyal N, Bouhired S, Hussaini A, Jones SW, et al. Pharmacokinetic interactions between tafenoquine and dihydroartemisininpiperaquine or artemether-lumefantrine in healthy adult subjects. Antimicrob Agents Chemother. 2016;60:7321–32.
- Green JA, Patel AK, Patel BR, Hussaini A, Harrell EJ, McDonald MJ, et al. Tafenoquine at therapeutic concentrations does not prolong Fridericia-corrected QT interval in healthy subjects. J Clin Pharmacol. 2014;54(9):995-1005.
- Haeusler, I.L., Chan, X.H.S., Guérin, P.J. et al. The arrhythmogenic cardiotoxicity of the quinoline and structurally related antimalarial drugs: a systematic review. BMC Med 16, 200 (2018). https://doi.org/10.1186/s12916-018-1188-2
- Hale BR, Owusu-Agyei S, Fryauff DJ, Koram KA, Adjuik M, Oduro AR, Prescott WR, Baird JK, Nkrumah F, Ritchie TL, Franke ED, Binka FN, Horton J, Hoffman SL. A randomized, double-blind, placebo-controlled, dose-ranging trial of tafenoquine for weekly prophylaxis against *Plasmodium falciparum*. Clin Infect Dis. 2003;36:541-549.
- Halliwell WH. Cationic amphiphilic drug-induced phospholipidosis. Toxicol Pathol. 1997;25:53-60.
- Hollander DA and Aldave AJ. Drug-induced corneal complications. Curr Opin Ophthalmol. 2004;15:541-8.
- Hounkpatin AB, Kreidenweiss A, Held J.; Clinical utility of tafenoquine in the prevention of relapse of *Plasmodium vivax* malaria: a review on the mode of action and emerging trial data. Infection and drug resistance. 2019;12:553-70.
- Howes RE, Piel FB, Patil AP, Nyangiri OA, Gething PW, Dewi M, Hogg MM, Battle KE, Padilla CD, Baird JK, et al. G6PD deficiency prevalence and estimates of affected populations in malaria endemic countries: a geostatistical model-based map. PLoS Med. 2012;9(11):e1001339. doi: 10.1371/journal.pmed.1001339.

- Hunter L, Gordge L, Dargan P I, Wood D M. Methaemoglobinaemia associated with the use of cocaine and volatile nitrites as recreational drugs: a review. British J Clin Pharm. 2011;72:18–26. http://doi.org/10.1111/j.1365-2125.2011.03950.x
- Karbwang J, Back DJ, Bunnag D, Breckenridge AM. 1990. Pharmacokinetics of mefloquine in combination with sulfadoxine-pyrimethamine and primaquine in male Thai patients with falciparum malaria. Bull World Health Organ 68:633–638.
- Kitchener SJ, Nasveld PE, Gregory RM, Edstein MD. Mefloquine and doxycycline malaria prophylaxis in Australian soldiers in East Timor. Med J Aust. 2005;182(4):168-71.
- Krintafel (tafenoquine succinate tablets) FDA (Food and Drug Administration) Advisory Committee Briefing Document; July 2018. https://www.fda.gov/media/114270/download
- Lacerda MVG, Llanos-Cuentas A, Krudsood S, Lon C, Saunders DL, Mohammed R, et al. Single-dose tafenoquine to prevent relapse of *Plasmodium vivax* malaria (DETECTIVE part 2). N Engl J Med. 2019;380:215–28.
- Leary KJ, Riel MA, Roy MJ, Cantilena LR, Bi D, Brater DC, van de Pol C, Pruett K, Kerr C, Veazey JM, Jr., Beboso R, Ohrt C. A randomized, double-blind, safety and tolerability study to assess the ophthalmic and renal effects of tafenoquine 200 mg weekly versus placebo for 6 months in healthy volunteers. Am J Trop Med Hyg. 2009;81:356-62.
- Lell B, Faucher J-F, Missinou MA, et al. Malaria chemoprophylaxis with tafenoquine: a randomised study. Lancet. 2000;355(9220):2041–2045.
- Llanos-Cuentas A, Lacerda MV, Rueangweerayut R, Krudsood S, Gupta SK, Kochar SK, et al. Tafenoquine plus chloroquine for the treatment and relapse prevention of *Plasmodium vivax* malaria (DETECTIVE part 1): a multicentre, doubleblind, randomised, phase 2b dose-selection study. Lancet (London, England). 2014;383(9922):1049-58.
- Llanos-Cuentas A, Lacerda MVG, Hien TT, et al. Tafenoquine versus primaquine to prevent relapse of *Plasmodium vivax* malaria (GATHER). N Engl J Med 2019;380:229-241.
- Miller AK, Harrell E, Ye L, Baptiste-Brown S, Kleim JP, Ohrt C, Duparc S, Mohrle JJ, Webster A, Stinnett S, Hughes A, Griffith S, Beelen AP. Pharmacokinetic interactions and safety evaluations of coadministered tafenoquine and chloroquine in healthy subjects. Br J Clin Pharmacol. 2013;76:858-867.
- Nasveld P, Brennan L, Edstein M, Kitchener S, Leggat P, Rieckmann K. A randomised, double blind comparative study to evaluate the safety, tolerability and effectiveness of tafenoquine and mefloquine for the prophylaxix of malaria in non-immune Australian soldiers. Abstract 326. Am J Trop Med Hyg. 2002a;67:255-256.
- Nasveld PE, Edstein MD, Reid M, et al. Randomized, double-blind study of the safety, tolerability, and efficacy of tafenoquine versus mefloquine for malaria prophylaxis in nonimmune subjects. Antimicrob Agents Chemother. 2010;54(2):792–798.
- NCT02488980: US Army Unpublished Clinical Study Report. A randomized, double blind, placebo controlled evaluation of weekly tafenoquine (WR 238605/SB252263) compared to mefloquine for chemosuppression of Plasmodium falciparum in Western Kenya (SB Document Number: SB-252263/RSD-101KZH/1), 2003.
- NCT02563496 ongoing trial: An Open Label, Non-comparative, Multicenter Study to Assess the Pharmacokinetics, Safety and Efficacy of Tafenoquine (SB-252263, WR238605) in the Treatment of Pediatric Subjects With Plasmodium Vivax Malaria.
- Ngouesse B, Basco LK, Ringwald P, Keundjian A, Blackett KN. Cardiac effects of amodiaquine and sulfadoxine-pyrimethamine in malaria-infected African patients. Am J Trop Med Hyg, 65 (2001), pp. 711-716

- Rueangweerayut R, Bancone G, Harrell EJ, et al. Hemolytic potential of tafenoquine in female volunteers heterozygous for glucose-6-phosphate dehydrogenase (G6PD) deficiency (*G6PD Mahidol* variant) versus G6PD-normal volunteers. Am J Trop Med Hyg 2017; 97: 702-11.
- Sanofi-Aventis U.S. LLC. Primaquine Phosphate (tablet, film coated) Drug Label Information. Revised: August 2016. Available from: https://dailymed.nlm.nih.gov/dailymed/drugInfo.cfm?setid=1BFBF4AE-81B8-4160-A00D-6322AADD4B59. Accessed: October 31, 2016.
- Shanks GD, Oloo AJ, Aleman GM, Ohrt C, Klotz FW, Braitman D, Horton J, Brueckner R. A new primaquine analogue, tafenoquine (WR 238605), for prophylaxis against *Plasmodium falciparum* malaria. Clin Infect Dis. 2001;33:1968-74.
- Waller M, Treloar SA, Sim MR, McFarlane AC, McGuire AC, Bleier J, Dobson AJ. Traumatic events, other operational stressors and physical and mental health reported by Australian Defence Force personnel following peacekeeping and war-like deployments. BMC psychiatry. 2012;12:1.
- Warrasak S, Euswas A, Fukuda MM, et al. Comparative ophthalmic assessment of patients receiving tafenoquine or chloroquine/primaquine in a randomized clinical trial for *Plasmodium vivax* malaria radical cure. *Int Ophthalmol*. 2019;39(8):1767–1782. doi:10.1007/s10792-018-1003-2
